# Cadmium Exposure Alters Rhizospheric Microbial Community and Transcriptional Expression of Vetiver Grass

**DOI:** 10.3389/fpls.2022.808844

**Published:** 2022-02-25

**Authors:** Bin Wu, Jia Li, Dinghua Peng, Ziru Wang, Heng Xu

**Affiliations:** ^1^College of Ecology and Environment, Chengdu University of Technology, Chengdu, China; ^2^State Key Laboratory of Geohazard Prevention and Geoenvironment Protection, Chengdu University of Technology, Chengdu, China; ^3^Key Laboratory of Bio-Resource and Eco-Environment of Ministry of Education, College of Life Sciences, Sichuan University, Chengdu, China

**Keywords:** vetiver grass, microbial community, whole transcriptome, cadmium, soil

## Abstract

Vetiver grass (*Chrysopogon zizanioides* L.) has been used to remediate cadmium (Cd)-contaminated soil, while there have been few studies on the influence of Cd exposure on the rhizospheric microbial community and transcriptional expression of *C. zizanioides*. In this study, we investigated the response of the rhizospheric microbial community and transcriptional expression of *C. zizanioides* in 20 mg/kg Cd-contaminated soil. The results showed that Cd levels in the roots and shoots of *C. zizanioides* reached 250.80 and 73.40 mg/kg, respectively. The Cd exposure changed the rhizospheric bacterial community, resulting in the significant enrichment of *Sphingomonas*, *Lysobacter*, and *Gemmatimonadetes* in Cd-contaminated soil. In addition, 880 and 3,419 differentially expressed genes were identified in the plant roots and shoots, respectively, in response to Cd stress. Among these, the overexpressed genes associated with redox homeostasis, glutathione (GSH) metabolism, cell wall biosynthesis, and transmembrane transport pathways were found to participate in Cd detoxification in *C. zizanioides.* These findings could be useful for understanding the selective variation of the rhizospheric microbial community and the detoxification mechanisms of *C. zizanioides* in Cd phytoremediation.

## Introduction

Long-term mining activity is one of the major sources of heavy metal contamination of soils (Zhou et al., 2018). Cadmium (Cd) is one of the most concerning elements in soil because it is highly toxic to plants and humans (O’Connor et al., 2018; Zhang Y. et al., 2021). Although Cd is a non-essential element for plants, it can enter plant tissues *via* the transporters of essential elements (e.g., calcium, zinc, and iron) and disturb plant growth by affecting the photosynthetic apparatus, carbohydrate metabolism, and nitrate absorption (Khan et al., 2017). The major concern regarding Cd pollution is the high risk of cancer, including lung, bladder, renal, prostate, and breast cancer, as it can affect cell proliferation, differentiation, DNA replication, and protein synthesis (Imseng et al., 2018). Therefore, there is a strong need for the development of low-cost remediation strategies for Cd-contaminated soil (Liu et al., 2018).

Phytoremediation has been widely examined as an effective, environment-friendly, and low-cost technology for the remediation of mine-contaminated soil (Sarwar et al., 2017). Plants can absorb heavy metals from the surrounding soil through their root system (Cui et al., 2020). Vetiver grass (*Chrysopogon zizanioides* L.) is a common herb that can effectively immobilize Cd. It has the advantages of being tall, fast-growing, and easily cultivable and has a vast root system (1–2 m) (Wu et al., 2020). Aibibu et al. (2010) found that Cd absorption by the roots of *C. zizanioides* can reach 2,200 mg/kg at a concentration of 30 mg/L Cd in water. Our previous study also found that *C. zizanioides* could effectively absorb Cd in soil (Wu et al., 2020). In addition, *C. zizanioides* can be grown in saline, lime, peat, low fertile soil conditions, and even in extreme temperatures (Attinti et al., 2017). Hence, vetiver grass has great potential for use in the bioremediation of Cd-contaminated soil.

The Cd toxicity is unfavorable for biological growth. In the soil-plant system, rhizospheric microorganisms and plants are the two main biological species. The rhizosphere is an important plant habitat, where metabolism, energy exchange, and signal transduction are much higher than that in other regions (Hu et al., 2018). The microbial community and diversity are closely related to soil contamination, especially by heavy metals (Ruvindy et al., 2016). Previous studies have found that excess concentrations of Cd can affect soil microbial diversity and might screen out some metal-tolerant bacterial species (e.g., *Pseudomonas*, *Acinetobacter*, and *Serratia*) (Jiang et al., 2019; Liu et al., 2020). Soil microorganisms are important participants in Cd mobility and plant detoxification (Sharma, 2021). Hence, it is important to investigate the changes in the rhizospheric microbial community under Cd stress. The excellent Cd tolerance of *C. zizanioides* is the basis of phytoremediation. In a previous study, we investigated the morphological and physiological characteristics of *C. zizanioides* in the presence of different Cd levels (Wu et al., 2020). However, the potential mechanisms, especially the molecular mechanisms, underlying the response of *C. zizanioides* to Cd stress, are still unknown. With the development of next-generation sequencing (NGS) technologies, whole transcriptome sequencing provides comprehensive gene expression profiles that can reveal the molecular mechanisms underlying the response of organisms to biotic and abiotic stresses (Hemme et al., 2010). Recently, changes in the whole transcriptomes of many species (e.g., *Hibiscus cannabinus* L., *Solanum lycopersicum* L., and *Sedum alfredii*) in response to Cd stress have been deeply investigated, revealing key insights into the Cd-tolerant strategies (Chen et al., 2020; Zhang et al., 2020). Cd detoxification pathways in plants were significantly different among different cultivars. For example, Pak Choi transcriptome analysis indicated that the differentially expressed genes (DEGs) in response to Cd stress were mainly involved in cell wall biosynthesis, glutathione (GSH) metabolism, and abscisic acid signal transduction pathways (Zhou et al., 2016). The DEGs in *S. lycopersicum* L. under Cd stress were mainly involved in plant hormone signal transduction, antioxidant enzymes, cell wall biosynthesis, and metal transportation (Su et al., 2021). However, the detoxification mechanisms of *C. zizanioides* under Cd stress are still unknown.

The main objectives of this study were to (1) investigate the growth response and Cd immobilization of vetiver grass in heavily Cd-contaminated soil; (2) reveal the changes of the rhizospheric microbial community under Cd stress; and (3) identify DEGs and elucidate the detoxification mechanisms used by vetiver grass in response to Cd stress.

## Materials and Methods

### Soil Preparation

Clean soil was collected from a farmland in Chengdu Plain, China. The soil was air-dried and passed through a 2-mm sieve. The physicochemical properties of the soil were as follows: pH 6.9, organic matter 42.46 g/kg, total N 2.68 g/kg, total P 0.78 g/kg, total K 16.97 g/kg, cation exchange capacity 19.70 cmol/kg, total Cd 0.6 mg/kg, total Cu 27.35 mg/kg, total Ni 18.34 mg/kg, and total Zn 33.18 mg/kg. Our previous study indicated that the morphological and physiological characteristics of *C. zizanioides* were significantly altered under the stress of 20 mg/kg Cd (Wu et al., 2020). In addition, Cd concentrations over 20 mg/kg were universally found in the abandoned mining areas in China, including Dexing Cu, Yangjiazhangzhi Mo-Cu, Hongqiling Ni, and Baiyin polymetallic ore deposits (Zhou et al., 2018). Hence, the test soil sample was carefully mixed with the quantitative CdCl_2_ solution, and the Cd concentration in the soil was set to 20 mg/kg. Subsequently, the soil samples were packaged into pots (height 13 cm and diameter 18 cm) at 2 kg/pot and long-aged for 8 months.

### Pot Experiment

A pot experiment was conducted in a greenhouse at Sichuan University. Before cultivation, *C. zizanioides* seeds (Pengyuan Seed Industry Co., Ltd., Guangdong, China) were cultured for 3 days for germination in darkness. Then, twenty healthy and uniform *C. zizanioides* sprouts were cultivated in clean soil (–Cd) and Cd-contaminated soil (+ Cd) in three replicates. During cultivation, the growth conditions were maintained at 14/10 h for day/night duration and 60% soil field water holding capacity. After 60 days of cultivation, the rhizosphere soil and fresh *C. zizanioides* were carefully collected. The rhizosphere soil, which is free of roots, was gently shaken off from *C. zizanioides* roots, frozen with liquid nitrogen, and stored at –80°C for microbiome analysis. Meanwhile, fresh *C. zizanioides* was chipped, frozen, and stored at –80°C for transcriptome analysis.

### Analysis of Cadmium Levels in *Chrysopogon zizanioides*

The plant roots and shoots were dried at 60°C and then ground into power. Plant samples (0.1 g) were digested by microwave digestion with 5:4:3 (v/v) of HNO_3_:HClO_4_:HF, and the Cd content was measured by atomic absorption spectroscopy (AAS; VARIAN, SpecterAA-220Fs).

### Analysis of Rhizosphere Microbiome

The genomic DNA of rhizosphere microbes was extracted using the E.Z.N.A.^®^ Soil DNA Kit (Omega Bio-Tek, Norcross, United States), according to the instructions of the manufacturer. The DNA extract was run on 1% agarose gel, and DNA concentration and purity were determined with NanoDrop 2000 UV-Vis spectrophotometer (Thermo Scientific, Wilmington, United States). The quality of DNA extract is shown in Supplementary Table 1. The 16S rRNA gene was amplified with primer pairs 338F (5′-ACTCCTACGGGAGGCAGCAG-3′) and 806R (5′-GGACTACHVGGGTWTCTAAT-3′) and purified using the AxyPrep DNA Gel Extraction Kit (Axygen Biosciences, Union City, CA, United States). Purified amplicons were pooled in equimolar amounts and paired-end sequenced (2 × 300) on an Illumina MiSeq platform (Illumina, San Diego, United States) by Majorbio Bio-Pharm Technology Co., Ltd. (Shanghai, China). The raw 16S rRNA gene sequencing reads were demultiplexed, quality-filtered by fastp version 0.20.0, and merged using FLASH version 1.2.7 (Chen et al., 2018). Operational taxonomic units (OTUs) with 97% similarity cutoff were clustered using UPARSE version 7.1, and chimeric sequences were identified and removed (Edgar, 2013). The taxonomy of each OTU representative sequence was analyzed using RDP Classifier version 2.2 against the 16S rRNA database (Silva v138), using a confidence threshold of 0.7. The Student’s *t*-test was carried out to test the normal distribution of the data. When data did not meet the normal distribution criteria, they were transformed using Box-Cox or Johnson’s function and were analyzed using a non-parametric rank-sum test (Kang et al., 2021). The non-metric multidimensional scaling (NMDS) using a weighted UniFrac distance matrix was carried out using R software (Kang et al., 2021). The linear discriminant analysis coupled with effect size analysis (LEfSe) was employed to explore statistically different biomarkers between the treated groups (Zhang X. et al., 2021).

### Analysis of the Plant Transcriptome

Total RNA was extracted from plant roots and shoots using TRIzol^®^ Reagent (Invitrogen, Carlsbad, CA, United States), and DNA was removed using DNase I (TaKaRa). Then, the total RNA content was determined by 2100 Bioanalyzer (Agilent Technologies, Santa Clara CA, United States). Subsequently, RNA purification, reverse transcription, library construction, and sequencing were performed at Shanghai Majorbio Bio-Pharm Biotechnology Co., Ltd. (Shanghai, China). The raw data were submitted to the NCBI SRA^1^ with ID number PRJNA772523. To validate the expression data obtained from RNA sequencing (RNA-Seq), eight genes with different expression levels were randomly selected and analyzed by quantitative PCR (qPCR). The primer sequences used in this study are listed in Supplementary Table 2. Three replicates were performed for three separate RNA extracts from three samples, and the results were calculated using 2^–ΔΔCT^.

The raw reads were trimmed and quality controlled, and the clean data thus obtained were used for *de novo* assembly with Trinity (Grabherr et al., 2011). All transcripts were searched against NR, String, and Kyoto Encyclopedia of Genes and Genomes (KEGG) databases using BLASTX (cutoff *E*-values < 1.0 × 10^–5^). BLAST2 Gene Ontology (GO) program was used to obtain GO annotations (Conesa et al., 2005). Meanwhile, the metabolic pathway analysis was performed using the KEGG (Ogata et al., 1999). To identify DEGs between −Cd and + Cd samples, the expression level of each transcript was calculated using the fragments per kilobase of exon per million mapped reads (FRKM) method. RNA-seq by expectation-maximization (RSEM)^2^ was used to quantify gene and isoform abundances (Li and Dewey, 2011). R statistical package software was used for differential expression analysis (Robinson et al., 2010). Furthermore, gene ontology (GO) and KEGG were used to identify DEGs that were significantly enriched in GO terms and metabolic pathways (corrected *p*-value ≤ 0.05, compared with control).

### Statistical Analysis

Data are presented as mean ± SD from three replicates. Statistical significance was analyzed using SPSS 18.0 package, and mean values were considered to be different when *p* < 0.05 using the least significant difference (LSD). All statistical analyses were performed using Origin 2020 (United States). The bioaccumulation factor (BCF) of Cd in plants was calculated using the following formula:


$$\mathrm{BCF}=\frac{\text{Cd}\text{accumulation}\text{content}\mathrm{in}\mathrm{plant}}{\text{Cd}\text{content}\text{in}\text{soil}}$$

## Results and Discussion

### Biomass and Cadmium Content of *Chrysopogon zizanioides*

**FIGURE 1:**
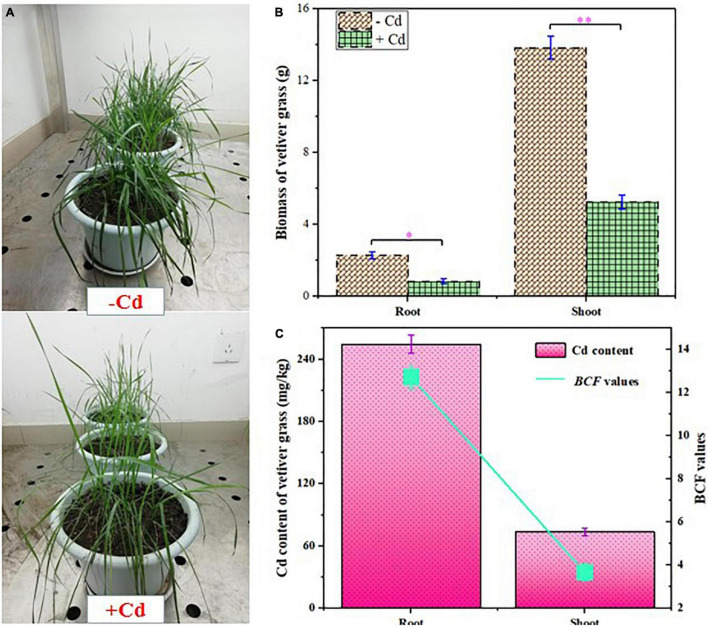
Growth response **(A)**, biomass **(B)**, and cadmium (Cd) content and bioaccumulation factor (BCF) values **(C)** of *Chrysopogon zizanioides*. Error bars represent *SD* values (*n* = 3). * and ** represent *p* < 0.05 and *p* < 0.01, respectively.

The growth and Cd content of *C. zizanioides* are presented in Figure 1. It was observed that Cd exposure significantly inhibited the growth of *C. zizanioides*. In the + Cd group, the biomass of root and shoot decreased from 13.66 and 2.09 mg/kg to 5.11 and 0.85 mg/kg, respectively. Although accumulators possess metal tolerance, their growth can be inhibited when the levels of heavy metals exceed their tolerance limit (Gao et al., 2010). In this study, the soil was heavily contaminated with 20 mg/kg Cd concentration, which was chosen to cause obviously adverse effects on plant growth (Wu et al., 2019). In contrast, the Cd concentrations in plant roots and shoots reached 250.80 and 73.45 mg/kg, respectively. The BCF values in plant roots and shoots also reached 12.54 and 3.67, respectively. Moreover, Cd accumulation in *C. zizanioides* tissues was significantly higher than other accumulators (Supplementary Table 3), which indicated that *C. zizanioides* had excellent potential for Cd phytoremediation, thus decreasing Cd mobility and bioavailability.

### Response of Rhizospheric Microbial Community

To reveal the changes of the microbial community in the rhizosphere in response to Cd toxicity, the rhizosphere microbiome of vetiver grass in + Cd and –Cd samples was analyzed by 16S rRNA sequencing. A total of 1,016,862 high-quality 16S rRNA gene reads were obtained and clustered into 2,317 OTUs. Of these, 106 and 120 unique OTUs were observed in –Cd and + Cd samples, respectively (Supplementary Figure 1A). Rarefaction and Shannon curves tended to be plain (Supplementary Figures 1B,C), which indicated that the samples were suitable for further analysis (Wu et al., 2018). Alpha-diversity indexes were widely used to evaluate microbial diversity (Xiao et al., 2017). It was observed that the alpha-diversity indexes, including Sobs, Shannon, Ace, and Chao, did not show a significant difference (*p* < 0.05) between –Cd and + Cd samples (Supplementary Table 4), indicating that Cd toxicity did not significantly affect the rhizospheric microbial diversity.

**FIGURE 2:**
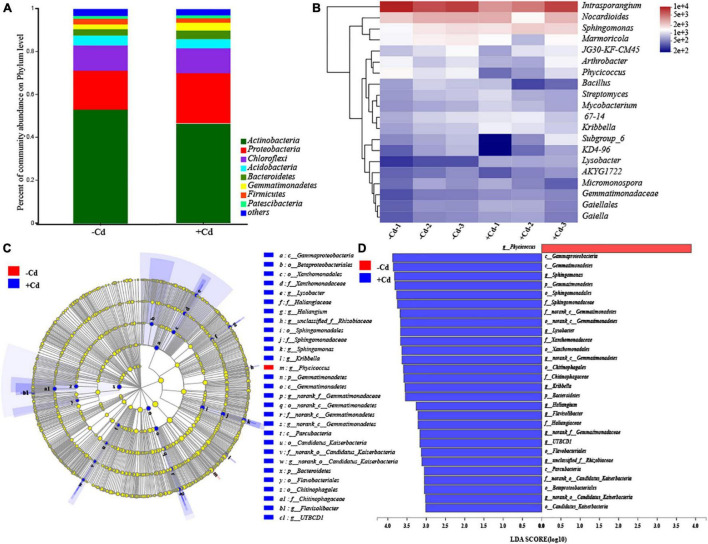
Comparison of bacterial communities in –Cd and + Cd samples. Changes in microbial community on phylum level **(A)**. Heatmap showing changes in microbial community on genus level **(B)**. Cladogram showing the phylogenetic distribution of the bacterial lineages in + Cd and –Cd samples **(C)**. Indicator bacteria with the linear discriminant analysis scores of 3 or greater in bacterial communities in + Cd and –Cd samples **(D)**. Different-colored regions represent different treatments. Circles indicate phylogenetic levels from phylum to genus. The diameter of each circle is proportional to the abundance of the group. p, phylum; o, order; c, class; f, family; g, genus.

The bacterial community structure in rhizosphere soil was analyzed, and the results are shown in Figure 2. The bacteria in rhizosphere soil were classified into 31 phyla (Figure 2A). The three dominant phyla across the samples were *Actinobacteria*, *Proteobacteria*, and *Chloroflexi*. The abundance of *Actinobacteria* was significantly higher than that of others, despite its percentage decreasing by 11.89% in the + Cd sample. In contrast, the proportion of *Proteobacteria* increased by 28.41% in the + Cd sample, compared to that in the –Cd sample. At the genus level (Figure 2B), there were 13 genera (apart from *norank*) with relative abundances higher than 1%, and the three most abundant genera were *Intrasporangium*, *Nocardioides*, and *Sphingomonas*. Compared to the –Cd sample, the abundance of *Intrasporangium* decreased by 33.66%, while that of *Sphingomonas* increased by 29.59% in the + Cd sample. Furthermore, the LEfSe tool was used to search biomarkers from phylum to genus level. Cladograms (Figure 2C) showed the different treatments, and the linear discriminant analysis scores of 3 or more were confirmed by LEfSe (Figure 2D). It was observed that a greater number of species were enriched in the + Cd sample compared to that in the –Cd sample. In the + Cd sample, when compared to the –Cd sample, it was observed that the abundances of *Sphingomonas*, *Lysobacter*, and *Gemmatimonadetes* significantly increased at the genus level by 29.73, 131.21, and 166.44%, respectively.

In the Cd-contaminated soil, the toxicity of Cd led to the selection of microorganisms that are tolerant or resistant to Cd exposure (Valls and de Lorenzo, 2002). The dominant phyla in our study were *Actinobacteria*, *Proteobacteria*, and *Chloroflexi*, which were widely identified previously in southwestern China (Wu et al., 2018). Among the most relevant alteration in the bacterial community, in response to Cd stress, was a decrease in the abundance of *Actinobacteria* (Ai et al., 2018; Sarria Carabalí et al., 2020). Previous studies also found that *Actinobacteria* was the most dominant phylum in environments such as plant rhizosphere, sewage sludges, hot springs, and freshwater habitats (Alvarez et al., 2017; Xiao et al., 2017). The reduction in the abundance of *Actinobacteria* might be related to the elimination of Cd-sensitive bacteria belonging to the phylum *Actinobacteria* due to high Cd toxicity (Sarria Carabalí et al., 2020). The positive response of *Proteobacteria* against heavy metals has also been observed in various metal-contaminated environments, such as metal-polluted soils, metal mine sediments, and river sediments (Lorenz et al., 2006; Zhang et al., 2014). The increase in *Proteobacteria* abundance might be attributed to the complex ecology of its lower taxonomic groups, which can adapt to environmental changes more readily than other phyla (Sandaa et al., 2001). Besides, members of *Proteobacteria* are well known for their metal tolerance (Rajeev et al., 2021). Previous studies found that *Proteobacteria* abundance was positively correlated with Cd concentration in soils (Muehe et al., 2015; Xiao et al., 2017). In this study, interestingly, some bacterial genera such as *Sphingomonas*, *Lysobacter*, and *Gemmatimonadetes* were significantly enriched in the + Cd sample, and these genera are already known for their metal tolerance (Pan et al., 2016; Wang et al., 2020). This study, therefore, indicates that Cd exposure in the heavily contaminated soil can alter the rhizospheric microbial community at the phylum and genus levels.

### Response of Plant Transcriptome

#### Transcriptome Assembly and Quantitative Real-Time PCR Validation

To obtain a comparative overview of the transcriptome of plant roots and shoots, cDNA libraries were constructed from –Cd to + Cd samples and sequenced using the Illumina HiSeqTM 2000 platform. After the removal of low-quality reads, 0.287 billion raw reads from roots, 0.301 billion raw reads from shoots, 0.278 billion clean reads from roots, and 0.292 billion clean reads from shoots were obtained in this study (Supplementary Table 5). A total of 219,363 unigenes corresponding to 521,416 transcripts, varying from 201 to 16,062 bp, were obtained with an average size of 634.98 bp (Supplementary Table 6). In addition, the N50 and E90N50 of unigenes of assembled genes were 933 and 2,101, respectively, and the GC percentage was 50.37%.

**FIGURE 3:**
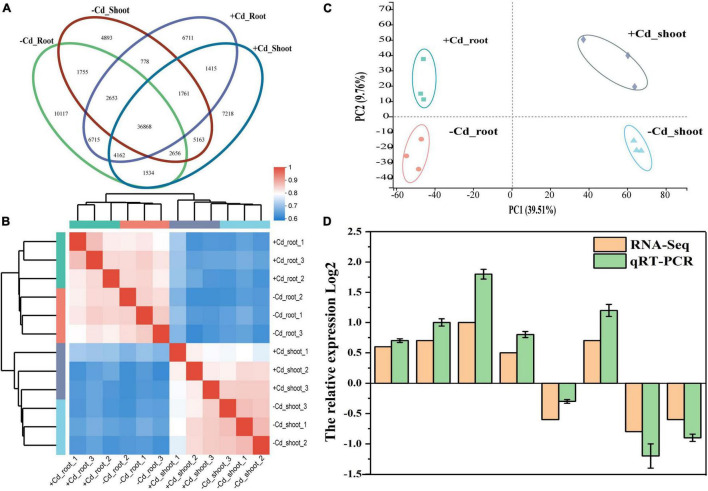
Venn diagram **(A)**, heatmap tree of groups **(B)**, principal component analysis (PCA) **(C)**, and quantitative real-time PCR (qRT-PCR) validation **(D)** of plant transcriptome.

Venn diagram showed that a total of 36,868 unigenes were shared among groups (Figure 3A). A total of 10,117 and 4,893 unigenes from roots and shoots, respectively, were specifically expressed in the –Cd sample, and a total of 6,711 and 7,218 unigenes from roots and shoots, respectively, were specifically expressed in the + Cd sample. Heatmap (Figure 3B) showed a high similarity coefficient among plant root and shoot samples. Principal component analysis (PCA) (Figure 3C) showed that the first principal component (PC1) reached 39.51%, which indicated a significantly different expression in shoots and roots. The second principal component (PC2) showed a significant difference in the unigene expression between the –Cd and + Cd samples. In addition, the quantitative real-time PCR (qRT-PCR) transcriptome data from eight randomly selected genes suggested that RNA-Seq data obtained from the samples were reliable (Figure 3D).

#### Identification and Functional Annotation of Differentially Expressed Genes in Response to Cadmium Stress

The DESeq2 analysis was performed with a *p*-adjusted value < 0.05 and a fold-change cutoff > 2, in order to identify the DEGs between –Cd and + Cd samples (Supplementary Figure 2). In roots, a total of 880 DEGs, including 471 upregulated DEGs and 409 downregulated DEGs, were identified. Meanwhile, the number of DEGs in the shoots was higher than that in the roots, including 2,054 upregulated DEGs and 1,365 downregulated DEGs.

**FIGURE 4:**
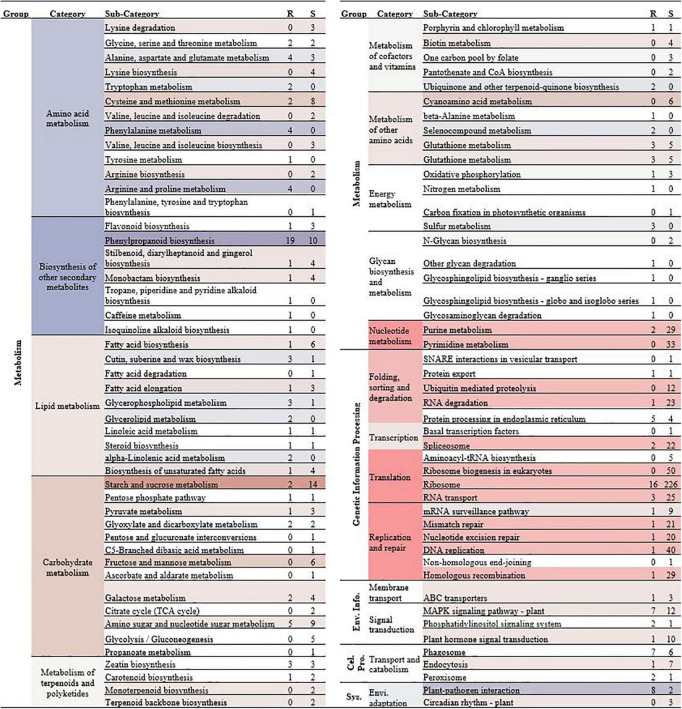
Normalized transcript expression comparison of root (R) and shoot (S) by + Cd vs. –Cd binned into the Kyoto Encyclopedia of Genes and Genomes (KEGG) categories. Genes only represented when DESeq2 *p*-adjusted value < 0.05 and the fold ratio > 2. Numbers in the category column represent the number of genes showing higher expression in the R or S, and the color shading is based on the difference between the expression in R and S (overrepresented in S, red; overrepresented in R, blue).

In this study, 161 upregulated genes from the roots and 755 overexpressed genes from the shoots fell within standard KEGG categories (Figure 4). Numerous overexpressed genes from shoots were associated with “translation” (40.53%) and “replication and repair” (15.89%). Various biological processes can be affected by Cd toxicity in plants, including genotoxicity and cytotoxicity. Genome stability is vital for DNA replication, gene expression, and protein synthesis in plant cells (Huang et al., 2019). Other studies have found that Cd appears to cause DNA damage mainly *via* the production of reactive oxygen species (ROS) and the inhibition of some DNA replication or repair enzymes, which could destabilize the genome and disturb the DNA replication system, e.g., by affecting mismatch repair (MMR), nucleotide excision repair (NER), and base excision repair (BER) (Gao et al., 2013; Tan et al., 2017). These genes involved in “translation” and “replication and repair” pathways were upregulated by Cd stress, possibly as a part of tolerance mechanisms to address Cd genotoxicity, in *C. zizanioides.* In particular, numerous overexpressed genes in shoots and roots were associated with “ribosome,” which might play an essential role in the Cd detoxification in *C. zizanioides*, because the ribosome plays a central role in protein synthesis (Xu et al., 2015). Apart from shoots, numerous overrepresented genes in roots were involved in “Phenylpropanoid biosynthesis,” “Plant-pathogen interaction,” “MAPK signaling pathway-plant,” and “Phagosome” pathways (Figure 4). These genes mainly coded regulatory and functional proteins, participating in signal transduction and functional regulation in plants under Cd stress (Li et al., 2018). Furthermore, GO enrichment showed that the upregulated DEGs in roots and shoots were both enriched in the –Cd vs. + Cd group and were involved in catalytic activity, transferase activity, metal ion binding, and antioxidant activity (Supplementary Tables 7, 8). These results further reveal the gene functional response of *C. zizanioides* under Cd stress.

#### Differentially Expressed Genes Analysis in Relation to Cadmium Detoxification in *Chrysopogon zizanioides*

**FIGURE 5:**
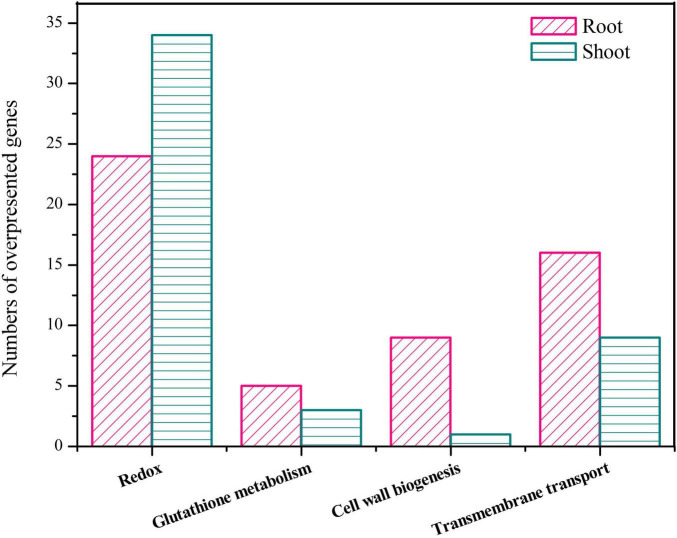
Number of overexpressed genes participating in redox homeostasis, glutathione metabolism, cell wall biosynthesis, and transmembrane transport.

Transcriptomic analysis showed that the upregulated DEGs were mainly involved in redox homeostasis, GSH metabolism, cell wall biogenesis, and transmembrane transport pathways (Figure 5).

In this study, 24 genes from roots and 34 genes from shoots associated with redox homeostasis were upregulated under Cd exposure, and most of these encode oxidases and reductases, such as peroxidase, monooxygenase, and cytochrome P450 (Supplementary Table 9). A total of 11 and 6 DEGs associated with peroxidase were identified in roots and shoots, and their maximum Log_2_FC values reached 5.78 and 9.85, respectively. However, DEGs between roots and shoots involved in redox homeostasis also showed a significant difference. Most DEGs in roots were associated with oxidase regulation and cytochrome P450, and the DEGs associated with peroxidase were especially enhanced. These results were consistent with our previous study, which demonstrated that antioxidase activities played an important role in Cd resistance in *C. zizanioides* (Wu et al., 2020). The intracellular redox homeostasis in plants is easily influenced by environmental stress (Hassan et al., 2005). Exposure to Cd can increase oxidative stress in plants by increasing the production of ROS, which can cause severe damage to major cellular macromolecules such as proteins, lipids, and DNA (Eckardt, 2008). The signal transduction pathways triggered in response to ROS stress can modulate the expression of specific downstream genes involved in plant detoxification. In addition, the antioxidant enzymes, including peroxidase, thioredoxin, oxidoreductase, GSH transferase, and catalase, are produced to reduce ROS-induced damage in plants (Mittler et al., 2004). Studies have also found that cytochrome P450 ameliorates Cd toxicity in plants by participating in fatty acyl metabolism, plant hormone synthesis, and secondary metabolite synthesis, thus enhancing stress response (Baker et al., 2001; Reynders et al., 2006). Moreover, our study found that numerous DEGs in shoots were connected to reductases, including ribonucleoside diphosphate reductase and 3-oxoacyl-CoA reductase, which are known to participate in DNA synthesis and energy regulation in plants (Coppey, 1977; Wengenmayer et al., 2010).

Glutathione metabolism might be a critical pathway for plant survival because GSH plays an essential role in the GSH-ascorbate redox system, which can effectively ameliorate hydrogen peroxide toxicity (Foyer and Noctor, 2011). Moreover, GSH is the precursor of plant peptides and GSH oligomers that can chelate heavy metals and transfer them into the vacuole, thus ameliorating metal toxicity (Dubey et al., 2016). In this study, 5 genes encoding GSH-*S*-transferase (GST) were overexpressed in response to Cd stress, and the maximum Log_2_FC reached 8.80 (Supplementary Table 10). GSTs are a superfamily of multifunctional enzymes that play crucial roles in the metabolism and intracellular homeostasis of GSH, such as catalyzing the binding of sulfhydryl groups and heavy metals or metalloids, for detoxification (Nianiou-Obeidat et al., 2017). Therefore, the increased expression of GSTs may help *C. zizanioides* in the process of Cd accumulation.

Roots are the first plant tissue to sense soil stress and also the first barrier preventing non-essential elements from entering the plant (Claire-Lise et al., 2015). Therefore, roots play a key role in helping plants reduce the toxicity of heavy metals and maintain their homeostasis (Lux et al., 2011). When heavy metals enter roots and shoots, most of them can be chelated and coprecipitated by polysaccharides such as lignin, cellulose, and hemicellulose, in the cell wall. During this process, some enzymes such as cellulase and glucanase can participate in cell wall biogenesis, thus contributing to heavy metal resistance (Huang et al., 2019). In this study, four overexpressed genes associated with xylanase inhibitor N-terminal, three overexpressed genes associated with glycosyl hydrolases, and one overexpressed gene associated with cellulose synthase were identified in the roots; but few genes involved in cell wall biosynthesis were overexpressed in shoots (Supplementary Table 11). In roots, most of the genes involved in cell wall biosynthesis were highly upregulated, and the maximum Log_2_FC reached 7.84. These results were positively correlated with Cd accumulation in plant roots (Figure 1), indicating that cell wall biogenesis contributes to higher Cd accumulation in *C. zizanioides* roots than that in shoots.

Furthermore, transmembrane transporters have been reported to play a crucial role in the uptake and transport of metal ions in plants (Aibara and Miwa, 2014). In response to Cd stress, 16 and 8 genes encoding transmembrane transporters were upregulated in roots and shoots, respectively, including inorganic phosphate transporters, ABC transporters, and zinc transporters (Supplementary Table 12). In addition, the expression of upregulated genes in roots (Log_2_FC 2.15–9.20) was significantly higher than that in shoots (Log_2_FC 2.21–3.95), which might be caused by the extent of Cd accumulation. Studies have suggested that ABC transporters such as OsHMA3 (Takahashi et al., 2012), OsABCC (Song et al., 2014), AtABCC1 (Patrizia et al., 2015), and AtABCC2 (Huang et al., 2019) are mainly located in the vacuolar membrane and could transport Cd into the vacuole, thus reducing Cd toxicity. Regional isolation of Cd may therefore be an important mechanism for plant detoxification.

### Possible Interaction Between Rhizospheric Microbial Communities and Plant Transcriptome

Previous studies found that the alteration of microbial communities under Cd stress increased the secretion of organic acids such as tartaric acid, malic acid, oxalic acid, and succinic acid, by microorganisms (Wang et al., 2021). These organic acids are known to affect the levels of minerals and metals in plants (Yuan et al., 2007). For example, oxalic acid and tartaric acid play a role in the rhizosphere by altering the pH of rhizospheric soil from 7.00 to 2.65 and by increasing the bioavailability of heavy metals from 8 to 96% (Li et al., 2010). In this study, *Sphingomonas*, *Lysobacter*, and *Gemmatimonadetes* were significantly enriched in the + Cd samples (Figure 2C). *Sphingomonas* has been previously found to be abundant in Cd-contaminated rhizospheric soil (Zhao et al., 2017). Most *Sphingomonas* are metal-activated strains, which can enhance Cd bioavailability by secreting organic acids (Wang et al., 2019). Chen et al. (2014) found that the incubation with *Sphingomonas* significantly enhanced Cd uptake by *S. alfredii*. In addition, *Sphingomonas* was frequently associated with dynamic biogeochemical processes such as iron or sulfate cycling, resulting in the release of dissolved Cd by sulfur oxidation (Wang et al., 2021). Consistent with other studies, Cd stress appeared to increase the relative abundance of *Lysobacter* in rhizospheric soil in this study (Li et al., 2022). *Lysobacter* are plant growth-promoting bacteria that can resist various pathogens and produce phytohormones, which could increase the uptake of heavy metals by accumulators (Laborda et al., 2020). Plant transcriptome factors control the entry of Cd from the soil into plant tissues (Su et al., 2021). The alteration of rhizospheric microbiology, due to the factors such as Cd stress, directly influences the plant transcriptome (Gu et al., 2019). The enrichment of *Sphingomonas* and *Lysobacter* in rhizospheric soil could increase Cd bioavailability and plant tolerance, which might be another reason why the genes associated with Cd tolerance and uptake, including those responsible for redox homeostasis, GSH metabolism, cell wall biogenesis, and transmembrane transport, are upregulated under Cd stress.

## Conclusion

This study revealed the impact of Cd exposure on the rhizospheric microbial community and transcriptome of *C. zizanioides*. *C. zizanioides* showed a great capacity for Cd accumulation, and its BCF value in roots reached 12.54, indicating a high capability to immobilize Cd in soil. On Cd exposure, the rhizospheric microbial community in *C. zizanioides* was significantly altered, resulting in a significant enrichment of *Sphingomonas*, *Lysobacter*, and *Gemmatimonadetes*. Meanwhile, Cd exposure significantly changed the transcriptome of *C. zizanioides* roots and shoots, and the overexpressed genes associated with redox homeostasis, GSH metabolism, cell wall biogenesis, and transmembrane transport had important effects on the Cd detoxification process in *C. zizanioides*.

## Data Availability Statement

The datasets presented in this study can be found in online repositories. The names of the repository/repositories and accession number(s) can be found below: https://www.ncbi.nlm.nih.gov/, PRJNA772523.

## Author Contributions

BW designed the project and performed the experiments. JL performed the statistical analysis and edited the manuscript. DP, ZW, and HX participated in the research and analyzed the data. All authors read and approved the final manuscript.

## Conflict of Interest

The authors declare that the research was conducted in the absence of any commercial or financial relationships that could be construed as a potential conflict of interest.

## Publisher’s Note

All claims expressed in this article are solely those of the authors and do not necessarily represent those of their affiliated organizations, or those of the publisher, the editors and the reviewers. Any product that may be evaluated in this article, or claim that may be made by its manufacturer, is not guaranteed or endorsed by the publisher.

## Footnotes

1
http://www.ncbi.nlm.nih.gov/sra

2
http://deweylab.biostat.wisc.edu/rsem/
