# Supplementary material for: Cadmium Exposure Alters Rhizospheric Microbial Community and Transcriptional Expression of Vetiver Grass
Source: Front Plant Sci. 2022 Feb 25;13:808844. doi: 10.3389/fpls.2022.808844 (PMC8914199; doi:10.3389/fpls.2022.808844)
Supplement: Supplementary file 1 [file Data_Sheet_1.doc]

**Supporting information**

**Cadmium exposure alters rhizospheric microbial community and transcriptional expression of vetiver grass**

Bin Wu a,b*[[1]](#footnote-2), Jia Li ^a^, Dinghua Peng^c^, Ziru Wang^c^, Heng Xu^c^

^a^ College of Ecology and Environment, Chengdu University of Technology, Chengdu, 610059, Sichuan, PR China

^b^ State Key Laboratory of Geohazard Prevention and Geoenvironment Protection, Chengdu University of Technology, Chengdu, 610059, Sichuan, PR China

^c^ Key Laboratory of Bio-Resource and Eco-Environment of Ministry of Education, College of Life Sciences, Sichuan University, Chengdu, 610065, PR China

**CONTENTS:**

**Table S1** The quality of 16S RNA extracted from soil bacteria.

**Table S2** The primer sequences used qPCR validation for the *C. zizanioides* transcriptome.

**Table S3** Comparison of Cd accumulation in *C. zizanioide*s with other accumulators.

**Table S4** The Alpha-diversity indexes of rhizosphere microbial community in the -Cd and +Cd treatments. Values followed with different lowercase letters indicated significant (*P* < 0.05) difference among different treatments.

**Table S5** Summary of Illumina transcriptome sequencing from plant roots and shoots.

**Table S6** Overview of sequencing assembly of accumulator.

**Table S7** GO enrichment analysis for *C. zizanioide*s roots exposed to Cd stress. The top 20 highly enriched genes with respect to unexposed plants for each treatment and their associated molecular functions are shown.

**Table S8** GO enrichment analysis for *C. zizanioide*s shoots exposed to Cd stress. The top 20 highly enriched genes with respect to unexposed plants for each treatment and their associated molecular functions are shown.

**Table S9** The identified candidate genes involved in redox in the -Cd and +Cd treatments.

**Table S10** The identified candidate genes involved in glutathione metabolism in the -Cd and +Cd treatments.

**Table S11** The identified candidate genes involved in cell wall biogenesis in the -Cd and +Cd treatments**.**

**Table S12** The identified candidate genes involved in transmembrane transport in different treatments.

**Figure S1** Venn diagram(A), Shannon curves (B) and Rarefaction curves (C) based on microbial OTUs levels.

**Figure S2** Volcano map of different expression genes of plant roots (A) and shoots (B) in -Cd and +Cd treatments.

**Schematic S1** Cd exposure altered the rhizospheric microbial community and transcriptional expression of vetiver grass.

**Table S1** The quality of 16S RNA extracted from soil bacteria.

| **Number** | **Concentrations (ng/ul)** | **OD260/280** | **OD260/230** |
| --- | --- | --- | --- |
| -Cd_1 | 13.80 | 1.96 | 0.94 |
| -Cd_2 | 9.90 | 1.88 | 0.76 |
| -Cd_3 | 15.00 | 1.86 | 0.98 |
| +Cd_1 | 19.40 | 1.91 | 1.05 |
| +Cd_2 | 16.20 | 1.82 | 0.81 |
| +Cd_3 | 17.10 | 1.66 | 0.61 |

**Table S2** The primer sequences used qPCR validation for the *C.zizanioide*s transcriptome.

| Gene ID | Primer sequences |
| --- | --- |
| TRINITY_DN82955_c0_g1 | F:AGCGTCGGATGAATACCTC  R:GATGGTCAGGGTTGTTTCC |
| TRINITY_DN79412_c4_g1 | F:ACCTCGGACGATAGATGAAG  R:CGTGTGCTGTGTTACTCAGG |
| TRINITY_DN55310_c0_g1 | F:GCCAATCATTATGCGGTC  R:ACTCAGGTGGGTGAAAGAGG |
| TRINITY_DN82860_c3_g2 | F: CGTTCACCTTAGGCTACTCG  R: GTCTTGTGGATTGACCGTC |
| TRINITY_DN63605_c0_g1 | F: CTATCAGGGTTGCGACCTT  R: TTCTGTTGGAGAGCCTTGC |
| TRINITY_DN56950_c3_g2 | F: CGTAGGCGATGTTCTCCAGG  R: CGCTTCAACCTCAAGTCGCT |
| TRINITY_DN52850_c0_g1 | F: ACGGAACCAGCAAACATAG  R: TCGGCATTCTTTACGACTG |
| TRINITY_DN55658_c2_g1 | F: CCAATGGTGATGTTACTGCG  R: CCTTTGTCCTCAGCAACTTG |
| TRINITY_DN94512_c1_g1 | F: ACCTCCAGAATGTAGCGAGC  R: GGTGCGGATAAATCTTACGG |
| TRINITY_DN58967_c0_g2 | F: TATGAAGTTCCCTGGCAGC  R: ACGATACAAATACGCCCG |

**Table S3 Comparison of Cd accumulation in *C. zizanioide*s with other accumulators.**

| Plants | Cd accumulation  (mg/kg) | Cd accumulated part in plants (mg/kg) | Concentration of Cd (mg/kg) | Medium | References |
| --- | --- | --- | --- | --- | --- |
| *Solanum torvum* L. | 105.43  44.58 | Root  Stem | 18.6 | Soil | (Dai et al., 2021) |
| *Hydrilla verticillata* | 42.65 | Whole plant | 20.0 | Soil | (Yuan et al., 2022) |
| *Elodea canadensis* | 22.61 | Whole plant | 20.0 | Soil | (Yuan et al., 2022) |
| Hybrid *Pennisetum* | 155.34  30.30 | Root  Stem | 20.0 | Soil | (Kamal et al., 2021) |
| *Napier grass* | 165.6 | Root | 50.3 | Soil | (Wiangkham and Prapagdee, 2018) |
| *Medicago sativa* L. | 8.57  9.14 | Root  Shoot | 35.0 | Soil | (Wang et al., 2021) |
| *Nicotiana tabacum* | 40.8  68.1 | Root  Shoot | 5.8 | Soil | (Li et al., 2022) |
| *Chrysopogon zizanioide*s L. | 250.80  73.40 | Root  Shoot | 20.0 | Soil | This study |

**Table S4** The Alpha-diversity indexes of rhizosphere microbial community in the -Cd and +Cd treatments. Values followed with different lowercase letters indicated significant (*P* < 0.05) difference among different treatments.

| Treatments | Sobs | Shannon | Ace | Chao | Coverage |
| --- | --- | --- | --- | --- | --- |
| Cd0 | 1977 ± 78a | 5.34 ± 0.19a | 2443 ± 68a | 2433 ± 63a | 0.99 ± 0.00a |
| Cd20 | 1948 ± 59a | 5.64 ± 0.09a | 2388 ± 34a | 2379 ± 41a | 0.99 ± 0.00a |

**Table S5** Summary of Illumina transcriptome sequencing from plant roots and shoots.

| Samples | Raw reads | Clean reads | Clean reads in raw reads |
| --- | --- | --- | --- |
| -Cd_root1 | 45,730,250 | 44,332,774 | 96.94 |
| -Cd_root2 | 47,123,744 | 45,472,184 | 96.50 |
| -Cd_root3 | 43,956,282 | 42,550,494 | 96.80 |
| +Cd_root1 | 49,623,982 | 48,106,072 | 96.94 |
| +Cd_root2 | 49,187,254 | 47,581,096 | 96.73 |
| +Cd_root3 | 51,625,498 | 49,944,274 | 96.74 |
| Subtotal | 287,247,010 | 277,986,894 | 96.78 |
| -Cd_shoot1 | 46,978,896 | 45,335,528 | 96.50 |
| -Cd_shoot2 | 53,238,904 | 51,543,596 | 96.82 |
| -Cd_shoot3 | 49,266,580 | 47,709,752 | 96.84 |
| +Cd_shoot1 | 51,009,008 | 49,445,684 | 96.94 |
| +Cd_shoot2 | 50,143,228 | 48,617,808 | 96.96 |
| +Cd_shoot3 | 50,363,414 | 48,849,066 | 96.99 |
| Subtotal | 301,000,030 | 291,501,434 | 96.84 |
| Total | 588,247,040 | 569,488,328 | 96.81 |

**Table S6** Overview of sequencing assembly of accumulator.

| Types | Numbers |
| --- | --- |
| Total transcripts | 521,416 |
| Total unigenes | 219,363 |
| Total sequence base (bp) | 139,292,008 |
| Largest isogene (bp) | 16,062 |
| Small isogene (bp) | 201 |
| Average length (bp) | 634.98 |
| N50 | 933 |
| E90N50 | 2101 |
| GC percentage (%) | 50.37 |

**Table S7** GO enrichment analysis for *C. zizanioide*s roots exposed to Cd stress. The top 20 highly enriched genes with respect to unexposed plants for each treatment and their associated molecular functions are shown.

| GO ID | Description | DEGs numbers |
| --- | --- | --- |
| GO:0003824 | Catalytic activity | 215 |
| GO:0043167 | Ion binding | 136 |
| GO:0016740 | Transferase activity | 84 |
| GO:0046872 | Metal ion binding | 70 |
| GO:0043169 | Cation binding | 70 |
| GO:0016491 | Oxidoreductase activity | 57 |
| GO:0020037 | Heme binding | 29 |
| GO:0046906 | Tetrapyrrole binding | 29 |
| GO:0016209 | Antioxidant activity | 20 |
| GO:0004601 | Peroxidase activity | 20 |
| GO:0070011 | Peptidase activity | 18 |
| GO:0004553 | Hydrolase activity | 15 |
| GO:0030410 | Nicotianamine synthase activity | 13 |
| GO:0008324 | Cation transmembrane transporter activity | 12 |
| GO:0008194 | UDP-glycosyltransferase activity | 9 |
| GO:0070008 | Serine-type exopeptidase activity | 7 |
| GO:0004564 | Beta-fructofuranosidase activity | 5 |
| GO:0004185 | Serine-type carboxypeptidase activity | 5 |
| GO:0004180 | Carboxypeptidase activity | 5 |
| GO:0045735 | Nutrient reservoir activity | 4 |
| GO:0030145 | Manganese ion binding | 4 |
| GO:0016884 | Carbon-nitrogen ligase activity | 4 |
| GO:0010181 | FMN binding | 4 |

**Table S8** GO enrichment analysis for *C. zizanioide*s shoots exposed to Cd stress. The top 20 highly enriched genes with respect to unexposed plants for each treatment and their associated molecular functions are shown.

| GO ID | Description | DEGs numbers |
| --- | --- | --- |
| GO:0003824 | Catalytic activity | 163 |
| GO:0046872 | Metal ion binding | 60 |
| GO:0043169 | Cation binding | 60 |
| GO:0016491 | Oxidoreductase activity | 49 |
| GO:0048037 | Cofactor binding | 32 |
| GO:0046906 | Tetrapyrrole binding | 26 |
| GO:0020037 | Heme binding | 26 |
| GO:0005215 | Transporter activity | 24 |
| GO:0022857 | Transmembrane transporter activity | 23 |
| GO:0008233 | Peptidase activity | 19 |
| GO:0004601 | Peroxidase activity | 18 |
| GO:0015075 | Ion transmembrane transporter activity | 14 |
| GO:0016765 | Transferase activity | 13 |
| GO:0008236 | Serine-type peptidase activity | 13 |
| GO:0017171 | Serine hydrolase activity | 13 |
| GO:0016825 | Hydrolase activity | 13 |
| GO:0030410 | Nicotianamine synthase activity | 11 |
| GO:0070008 | Serine-type exopeptidase activity | 9 |
| GO:0004857 | Enzyme inhibitor activity | 9 |
| GO:0008238 | Exopeptidase activity | 9 |
| GO:0030234 | Enzyme regulator activity | 9 |
| GO:0098772 | Molecular function regulator | 9 |
| GO:0005506 | Iron ion binding | 9 |

**Table S9** The identified candidate genes involved in redox in the -Cd and +Cd treatments. The sequences of identified genes were presented at the bottom of this supplementary file.

| Type | Gene ID | Log_2_FC | *p*-value | Gene function |
| --- | --- | --- | --- | --- |
| Root  -Cd vs +Cd20 | DN51954_c0_g1 | 6.27 | 2.39E-05 | Enoyl reductase |
|  | DN69059_c0_g1 | 5.78 | 2.16E-18 | Peroxidase 1 |
|  | DN68939_c1_g4 | 5.64 | 1.01E-04 | Peroxidase 5 |
|  | DN75075_c0_g3 | 5.54 | 2.75E-05 | Bifunctional dihydroflavonol 4-reductase |
|  | DN54394_c0_g1 | 5.51 | 2.47E-04 | Choline monooxygenase |
|  | DN67361_c0_g1 | 4.55 | 3.48E-06 | Peroxidase 2 |
|  | DN99744_c0_g2 | 4.51 | 8.69E-05 | Laccase |
|  | DN93689_c0_g1 | 3.99 | 1.36E-05 | Peroxidase 2 |
|  | DN87778_c0_g2 | 3.45 | 2.32E-05 | Peroxidase 2 |
|  | DN92367_c1_g4 | 3.04 | 5.61E-04 | Flavin-containing monooxygenase |
|  | DN93689_c1_g2 | 2.95 | 2.94E-04 | Peroxidase 2 |
|  | DN63605_c0_g1 | 2.95 | 1.60E-08 | Peroxidase 2 |
|  | DN76046_c0_g2 | 2.86 | 2.04E-04 | Class III peroxidase |
|  | DN81688_c0_g2 | 2.80 | 3.93E-04 | Peroxidase 1 |
|  | DN98601_c1_g1 | 2.78 | 8.73E-07 | Probable cinnamyl alcohol dehydrogenase 6 |
|  | DN84039_c0_g1 | 2.77 | 6.24E-04 | Cinnamoyl-CoA reductase 2 |
|  | DN68939_c0_g1 | 2.77 | 1.64E-04 | Peroxidase 1 |
|  | DN74633_c1_g4 | 2.76 | 1.93E-04 | Peroxidase P7 isoform X1 |
|  | DN71115_c0_g1 | 2.73 | 7.84E-05 | 2'-deoxymugineic-acid 2'-dioxygenase |
|  | DN86095_c0_g1 | 2.41 | 5.05E-04 | Peroxidase 21 |
|  | DN71437_c0_g1 | 2.40 | 1.75E-04 | Peroxidase 2 |
|  | DN88338_c2_g1 | 2.39 | 3.32E-04 | Cytochrome P450 89A2 |
|  | DN92728_c1_g2 | 2.32 | 2.46E-05 | Putative quinone-oxidoreductase homolog |
|  | DN79687_c0_g5 | 2.01 | 5.90E-04 | Cytochrome P450 76M5 |
| Shoot  -Cd0 vs +Cd20 | DN85257_c1_g1 | 9.85 | 7.96E-06 | Peroxidase |
|  | DN100686_c1_g5 | 7.46 | 1.75E-15 | Ribonucleoside-diphosphate reductase |
|  | DN83694_c1_g1 | 7.39 | 1.09E-05 | Peroxidase |
|  | DN90429_c1_g3 | 6.96 | 1.62E-03 | Glutamyl-tRNA reductase |
|  | DN86126_c0_g1 | 6.76 | 1.15E-03 | Salutaridine reductase |
|  | DN76086_c0_g1 | 6.22 | 9.94E-25 | Ribonucleoside-diphosphate reductase |
|  | DN100686_c1_g3 | 6.07 | 1.55E-25 | Ribonucleoside-diphosphate reductase |
|  | DN90778_c0_g1 | 5.83 | 1.98E-14 | Zinc-finger domain of monoamine-oxidase |
|  | DN74659_c2_g1 | 5.63 | 1.42E-05 | Lipoxygenase |
|  | DN77497_c2_g1 | 5.25 | 4.42E-06 | Peroxidase |
|  | DN85886_c0_g6 | 3.71 | 9.29E-04 | 3-oxoacyl-CoA reductase |
|  | DN70474_c0_g3 | 3.68 | 8.36E-06 | 4-hydroxy-tetrahydrodipicolinate synthase |
|  | DN76479_c0_g1 | 3.66 | 3.90E-05 | Zinc finger BED domain-containing protein |
|  | DN86845_c0_g2 | 3.64 | 3.68E-12 | Zinc-finger domain of monoamine-oxidase |
|  | DN60089_c2_g4 | 3.48 | 9.51E-05 | Salutaridine reductase |
|  | DN81556_c0_g2 | 3.32 | 2.49E-05 | Peroxidase |
|  | DN64173_c2_g3 | 3.24 | 1.08E-03 | Peroxidase |
|  | DN83151_c1_g1 | 3.12 | 4.49E-05 | Saccharopine dehydrogenase |
|  | DN98969_c1_g1 | 3.07 | 1.43E-03 | Cellulose synthase |
|  | DN95036_c2_g1 | 3.03 | 3.73E-06 | Ribonucleotide reductase |
|  | DN75230_c0_g1 | 2.94 | 2.50E-04 | 3-oxoacyl-CoA reductase |
|  | DN87390_c0_g3 | 2.88 | 7.21E-06 | Glucomannan 4-beta-mannosyltransferase |
|  | DN64596_c1_g1 | 2.88 | 7.78E-08 | 9-cis-epoxycarotenoid dioxygenase |
|  | DN79085_c0_g1 | 2.75 | 9.14E-04 | Putative bifunctional dihydrofolate reductase |
|  | DN83175_c0_g2 | 2.73 | 7.67E-04 | GMC oxidoreductase |
|  | DN101477_c1_g1 | 2.71 | 3.75E-05 | 9-cis-epoxycarotenoid dioxygenase |
|  | DN98385_c1_g1 | 2.70 | 1.17E-05 | Fatty acid hydroxylase superfamily |
|  | DN85366_c0_g3 | 2.37 | 3.21E-06 | Probable 4-hydroxy-tetrahydrodipicolinate reductase |
|  | DN78281_c0_g1 | 2.32 | 2.77E-04 | Peroxidase |
|  | DN89118_c1_g1 | 2.22 | 2.42E-04 | NAD dependent epimerase/dehydratase |
|  | DN85886_c0_g5 | 2.22 | 2.09E-03 | Dehydrogenase |
|  | DN85366_c0_g1 | 2.21 | 2.37E-06 | Probable 4-hydroxy-tetrahydrodipicolinate reductase |
|  | DN92896_c0_g2 | 2.13 | 1.25E-05 | Enoyl-[acyl-carrier-protein] reductase [NADH] |

**Table S10** The identified candidate genes involved in glutathione metabolism in the -Cd and +Cd treatments. The sequences of identified genes were presented at the bottom of this supplementary file.

| Type | Gene ID | Log_2_FC | *p*-value | Gene function |
| --- | --- | --- | --- | --- |
| Root  -Cd vs +Cd | DN70701_c1_g2 | 8.80 | 5.33E-08 | Glutathione S-transferase U24 |
|  | DN50518_c0_g1 | 4.60 | 1.93E-08 | Glutathione S-transferase GstA |
|  | DN53663_c0_g1 | 4.25 | 2.37E-05 | Pyridoxal-dependent decarboxylase |
|  | DN53607_c0_g1 | 3.35 | 2.64E-04 | Glutathione S-transferase |
|  | DN77185_c2_g1 | 3.01 | 5.35E-04 | Glutathione transferase GST 23 |
| Shoot  -Cd0 vs +Cd | DN62218_c0_g2 | 4.27 | 2.21E-07 | Hypothetical protein |
|  | DN76086_c0_g1 | 6.22 | 9.94E-25 | Ribonucleoside-diphosphate reductase |
|  | DN91181_c0_g1 | 2.41 | 1.70E-03 | Glutathione S-transferase |

**Table S11** The identified candidate genes involved in cell wall biogenesis in the -Cd and +Cd treatments**.** The sequences of identified genes were presented at the bottom of this supplementary file.

| Type | Gene ID | Log_2_FC | *p*-value | Gene function |
| --- | --- | --- | --- | --- |
| Root  -Cd vs +Cd | DN85177_c0_g5 | 7.84 | 1.02E-06 | Xylanase inhibitor N-terminal |
|  | DN77249_c1_g1 | 6.28 | 2.66E-07 | Xylanase inhibitor N-terminal |
|  | DN108310_c0_g1 | 5.50 | 2.91E-05 | Xylanase inhibitor N-terminal |
|  | DN96587_c1_g1 | 5.41 | 2.33E-04 | Glycosyl hydrolases family 18 |
|  | DN76141_c1_g4 | 5.17 | 7.60E-05 | Glycosyl hydrolases family 1 |
|  | DN79714_c0_g2 | 4.72 | 3.81E-05 | Glycosyl hydrolases family 18 |
|  | DN78248_c0_g1 | 3.88 | 8.35E-05 | Glycosyl hydrolases family 1 |
|  | DN73750_c1_g1 | 3.35 | 6.58E-06 | Xyloglucan endotransglucosylase |
|  | DN77114_c0_g2 | 2.06 | 5.35E-04 | Cellulose synthase |
| Shoot  -Cd vs +Cd | DN75875_c0_g1 | 3.74 | 9.93E-05 | Probable xyloglucan endotransglucosylase |

**Table S12** The identified candidate genes involved in transmembrane transport in different treatments. The sequences of identified genes were presented at the bottom of this supplementary file.

| Type | Gene ID | Log_2_FC | *p*-value | Gene function |
| --- | --- | --- | --- | --- |
| Root  -Cd vs +Cd | DN73397_c1_g4 | 9.20 | 1.23E-10 | Inorganic phosphate transporter |
|  | DN87006_c2_g1 | 6.92 | 6.21E-06 | ABC transporter |
|  | DN74986_c1_g1 | 5.51 | 2.93E-04 | Phosphate transporter family |
|  | DN40831_c0_g1 | 5.18 | 5.78E-05 | Putative zinc transporter 10 |
|  | DN55460_c0_g1 | 4.94 | 2.70E-04 | ATP-binding cassette protein |
|  | DN66524_c2_g4 | 4.09 | 1.12E-07 | Protein zinc induced facilitator |
|  | DN76268_c0_g2 | 3.83 | 1.81E-08 | Zinc transporter 1 |
|  | DN76268_c0_g1 | 3.81 | 6.10E-17 | Zinc transporter 1 |
|  | DN81633_c1_g1 | 3.57 | 4.79E-05 | ABC transporter |
|  | DN79236_c0_g1 | 3.35 | 2.36E-04 | ABC transporter |
|  | DN93260_c0_g1 | 3.17 | 3.42E-05 | ABC transporter |
|  | DN57749_c0_g1 | 3.11 | 2.38E-04 | Zinc transporter 9 |
|  | DN93774_c1_g1 | 2.84 | 2.81E-08 | ABC transporter |
|  | DN79723_c2_g3 | 2.78 | 4.49E-04 | ABC transporter |
|  | DN89057_c1_g1 | 2.31 | 4.20E-09 | Zinc transporter |
|  | DN99764_c1_g3 | 2.15 | 2.45E-06 | ABC transporter |
| Shoot  -Cd vs +Cd | DN55310_c0_g1 | 3.95 | 2.59E-03 | ABC transporter |
|  | DN102612_c6_g1 | 3.95 | 4.39E-06 | ABC transporter |
|  | DN56950_c3_g2 | 3.81 | 1.18E-03 | ABC transporter B |
|  | DN62657_c0_g1 | 3.30 | 4.30E-05 | ABC-2 type transporter |
|  | DN87048_c1_g1 | 3.19 | 2.09E-03 | ABC transporter F |
|  | DN53412_c0_g1 | 3.16 | 1.08E-03 | Metal ion transporter ZIP3 |
|  | DN74651_c1_g4 | 3.15 | 6.37E-04 | ABC transporter G |
|  | DN96918_c1_g1 | 3.04 | 8.06E-07 | Triose-phosphate Transporter |
|  | DN81110_c0_g1 | 2.21 | 6.18E-05 | Transmembrane protein 136 |

A


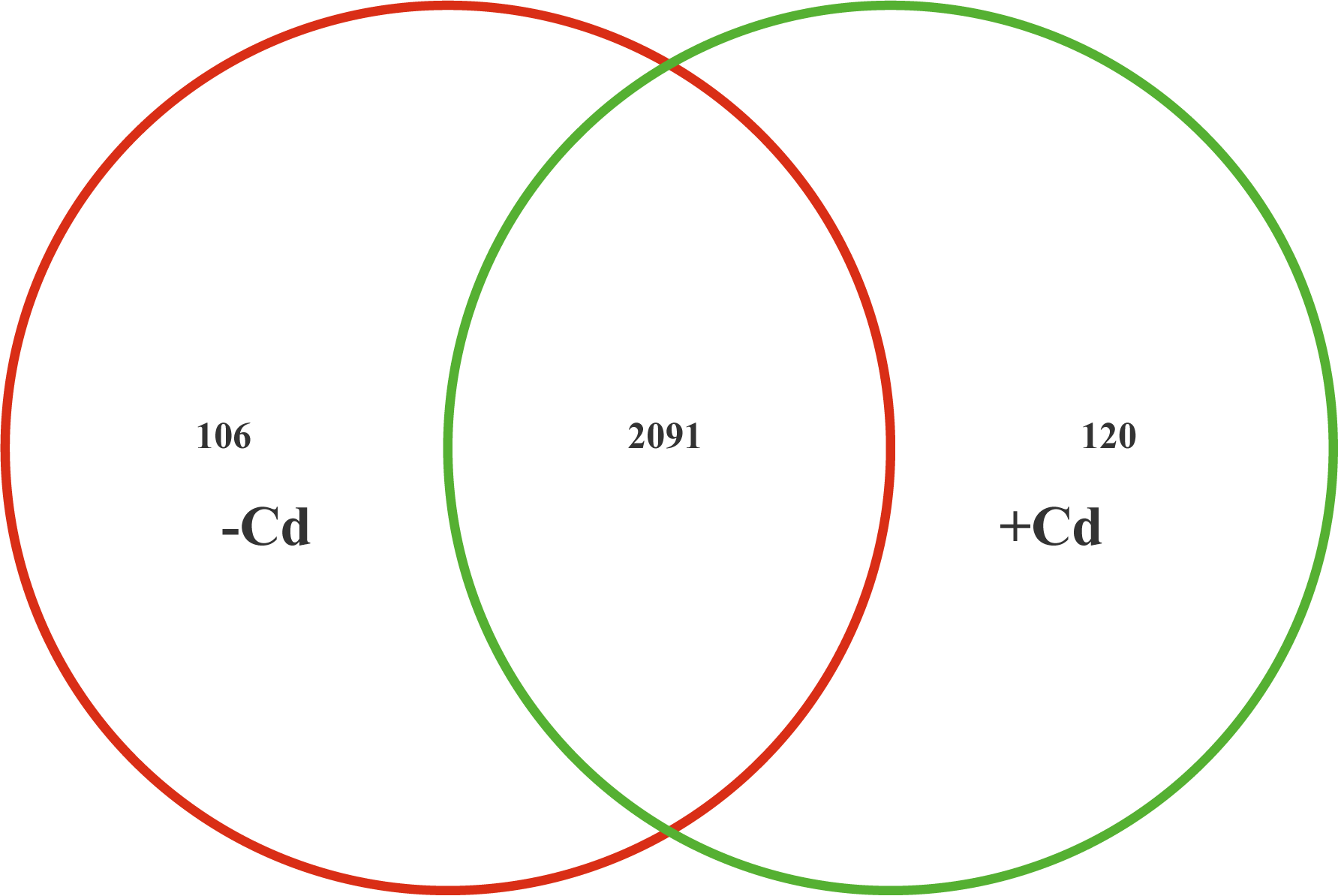


B


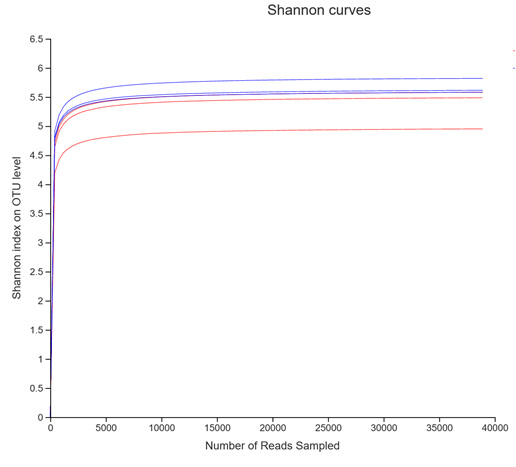


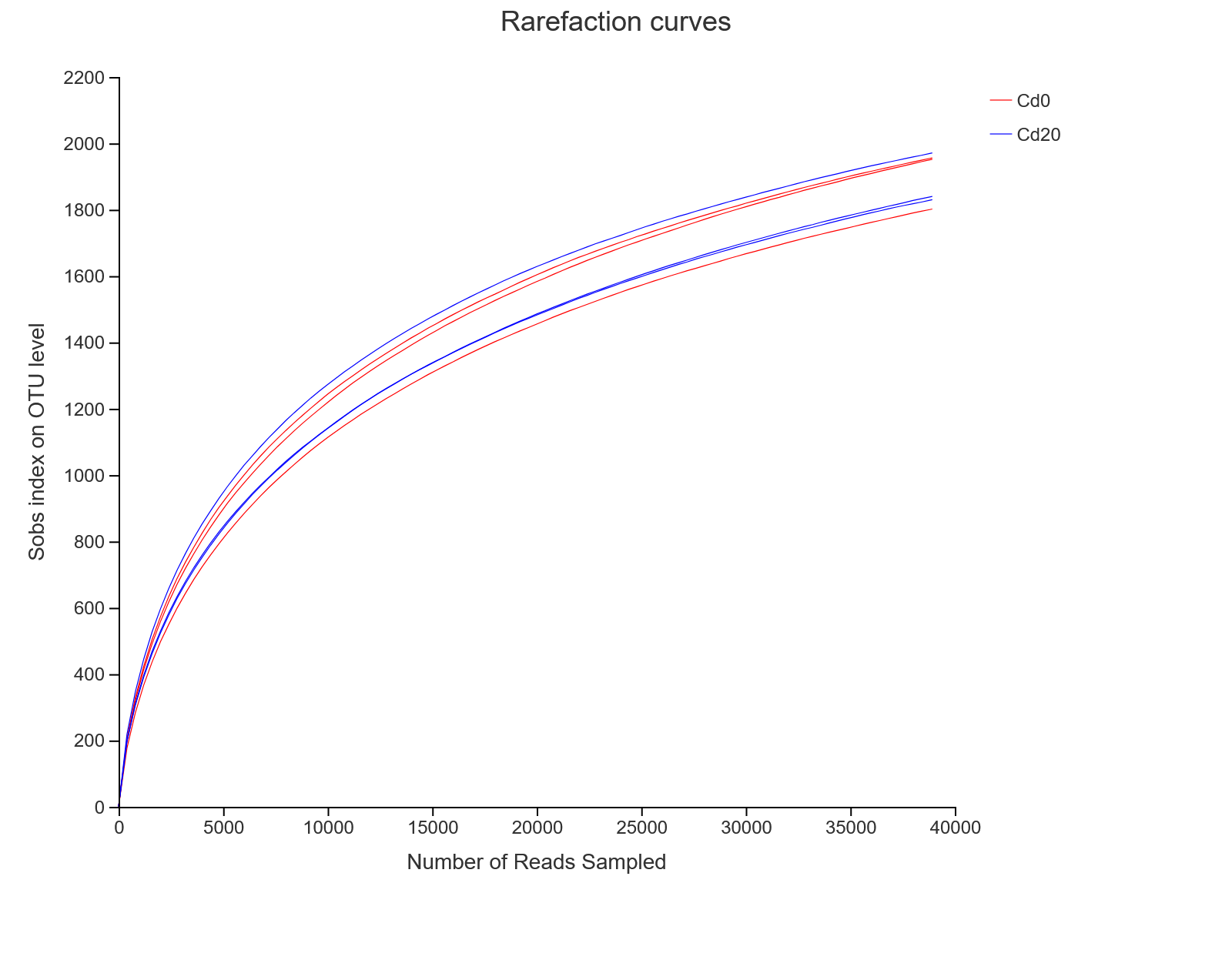


C

**Figure S1** Venn diagram(A), Shannon curves (B) and Rarefaction curves (C) based on microbial OTUs levels.

A

B


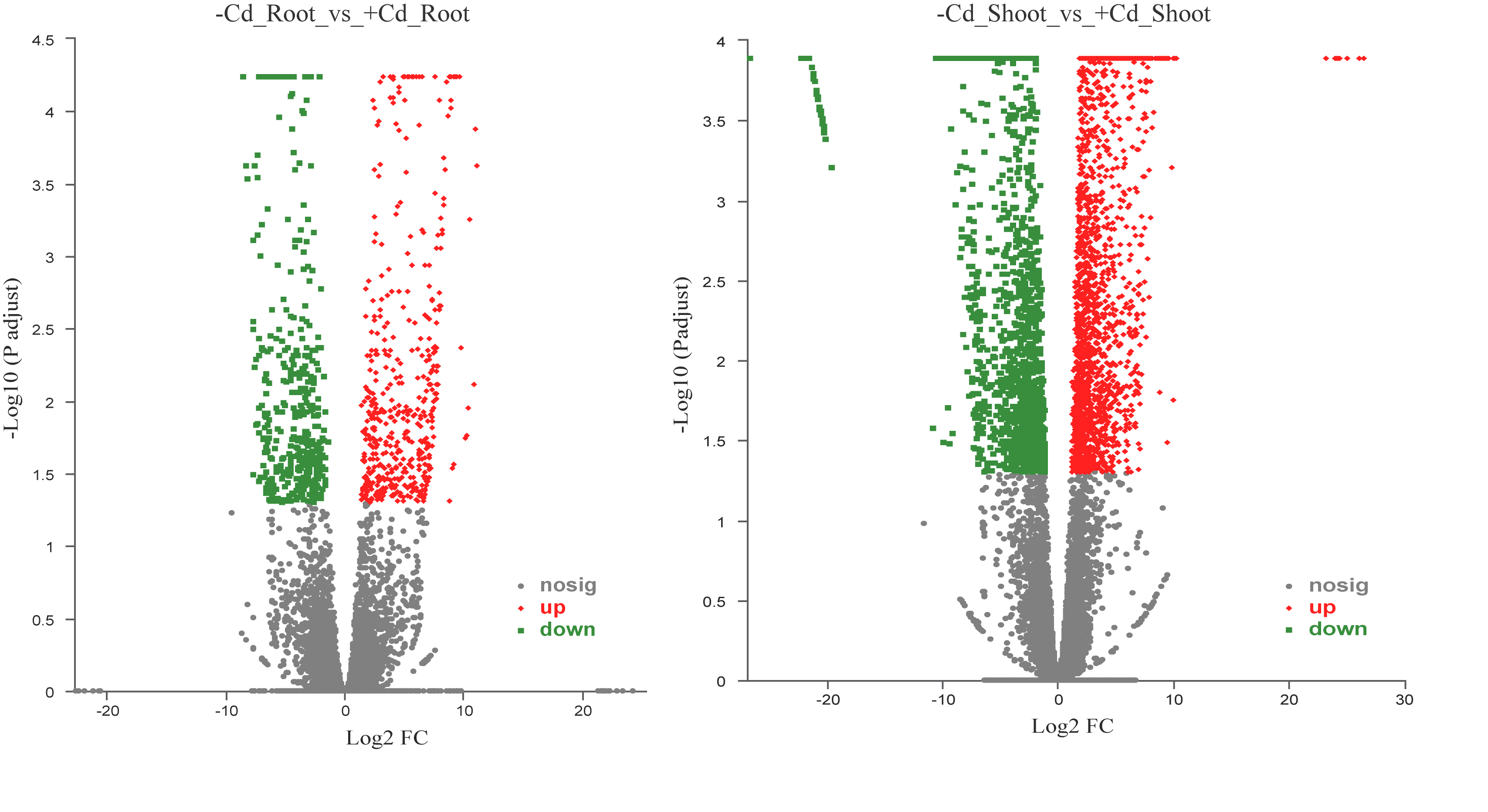


**Figure S2** Volcano map of different expression genes of plant roots (A) and shoots (B) in -Cd and +Cd treatments.


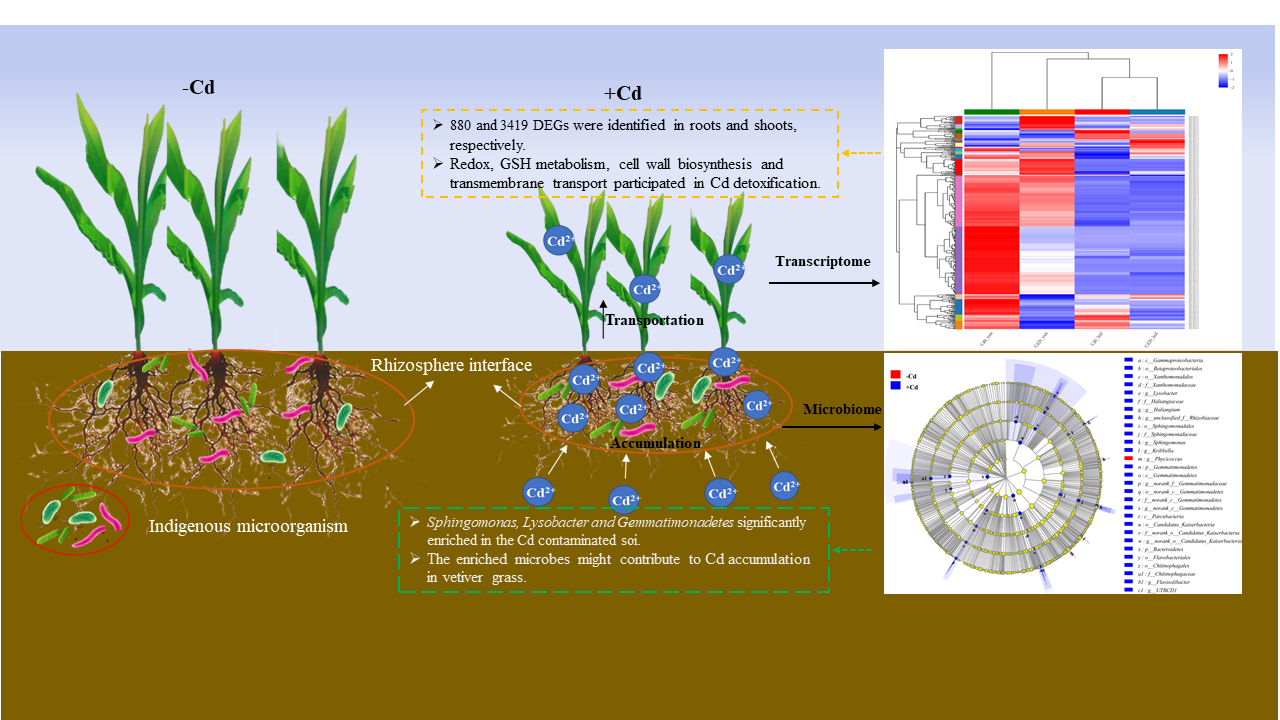


**Schematic S1** Cd exposure altered the rhizospheric microbial community and transcriptional expression of vetiver grass.

**References:**

Dai H, Wei S, Twardowska I, Zhang Q. In search of the exclusion/low-accumulation mechanisms: Cadmium uptake and accumulation from soil by cultivated (Solanum melongena L.) and wild eggplants (Solanum torvum L.). Journal of Cleaner Production 2021; 323: 129141.

Kamal N, Liu Z, Qian C, Wu J, Zhong X. Improving hybrid Pennisetum growth and cadmium phytoremediation potential by using Bacillus megaterium BM18-2 spores as biofertilizer. Microbiological Research 2021; 242: 126594.

Li X, Li Y, Zhu X, Gui X, Ma C, Peng W, et al. Evaluation of the cadmium phytoextraction potential of tobacco (Nicotiana tabacum) and rhizosphere micro-characteristics under different cadmium levels. Chemosphere 2022; 286: 131714.

Wang Y, Yang R, Hao J, Sun M, Wang H, Ren H. The impact of Pseudomonas monteilii PN1 on enhancing the alfalfa phytoextraction and responses of rhizosphere soil bacterial communities in cadmium-contaminated soil. Journal of Environmental Chemical Engineering 2021; 9: 106533.

Wiangkham N, Prapagdee B. Potential of Napier grass with cadmium-resistant bacterial inoculation on cadmium phytoremediation and its possibility to use as biomass fuel. Chemosphere 2018; 201: 511-518.

Yuan Q, Wang P, Wang X, Hu B, Tao L. Phytoremediation of cadmium-contaminated sediment using Hydrilla verticillata and Elodea canadensis harbor two same keystone rhizobacteria Pedosphaeraceae and Parasegetibacter. Chemosphere 2022; 286: 131648.

**List of sequences of identified genes in Table S9.**

1. Gene ID: DN51954_c0_g1

GCTCTATCCTTTCTCCACGCTTCGAGGCAGGTAAGATCAGGAGCGCATCGATCACCTGGTCTCTCTAATGGCATCGCTTCAAGTCACGCAGGAGCAGGAGCGCATCACCTCGCCTCCAATGGCGTCGCTGCTGTGCCCACCCTACAAGGCTGTCATGTACGACCAATACGGCGAGCCGGACAAGGTTCTGAGGTTGGCGGATGTTGGGCCAGTGAAGCTTGGGGAGAGAGACGTGTGCGTGAAGATGCTGGCCGCCCCCATCAACCCCTCCGACATCAACAGCATCGAGGGCGTCTACCCCAATAAGCCTACCGTTCCCGGCGCCGTCGGCGGAATCGAGGGCGTCGGACAGGTCCTAGCCGTCGGCCCGGCCGTCACCGCCTCTCTCTCTCCCGGCGACTGGGTCATCGCTTTTCCGCACACCGCCATGGGAACCTGGCAAACCTACGCCGTGAAACCGCAAGAGTTGTGGCACAAGGTCCGCAACGATGTGCCCATGGAATACGCCGCCACCGTCACAGTCAACCCCTTGTCAGCACTTGTGCTGCTGCAATGCTTCACCAAACTGAATCCTGGTGATACCATTGTCCAGAACGGTGCCACCAGCATTGTTGGTCAGTGCGTTATTCAGCTCGCTAAGGCGCAGGGAATTCGCACCATCAACATCATCAGGGACAGGCCTGGGTCCGAACAAGCCAAACACAGACTTACACAGCTTGGTGCACATCTGGTGTTCACCGAAAGCCAGCTACGTGTAAAGAATGTCAAGATTTTACTGGGTACATTGCCAGAGCCAGCATTGGGGTTTGACTGTGTAGGAGGAAACGCCGCATCTTTGACAATGAGGATCTTAAGGAAAGGAGGTACCATGGTGACGTATGGTGGGATGTCCAAGGAACATGTGAATGTTTCCACTTCATCTTTCATTTTTAAGGATCTATCCTTCAAAGGGTTCTGGCTATGGAATTGGGTGAATTCAGACAAGGTTGAAGATTGCAAGAAAATGATAGACTACCTCTTGGACCTGGTGAAGGAAGGCAAGCTAACATATCAGACGGAGTTGTCTCCCTTCCGTGATTTCGGATTGGCGCTCGATAAGGCACAAGGTAAACTGGGAGCCCAACCAAAGCAGGTTGTCAGTTTTTGGGACAAGGCTAAGTTGTAATACATCAAATATTCTGCACGTGCGAAAGGGAACGAATTATAGG

1. Gene ID: DN69059_c0_g1

GGTAGCAAGCGGCGGGCAATTCAGCAATCGGAGATGGCGTCGAGATCATCGGCTCTTGCTGCCCTCAATGTCGTTGCGCTCCTCGTCCTGTGCGGCCTTCCGGCGACGACGGAGGCCCACACGCAGTTCGGGGCGTACAACAAGACGTGCCCCCAGGCGGAGGAGATCGTCTTCAAGGAGATGACTGCCATCCTGGCAAAGTCGCCGGACCTCGCCGGACCAGTTCTCCGGCTCTTCTCCGTCGACTGCTTGCTCGGAGGCTGCGAGGGGTCGATTCTTCTGAACTCGACTGCCAACAACACGGCGGAGAAGGACGCGCCGCTCAACAAGGGCCTCCGTGGCTACGACGTCGTCGACTCTATCAAGGCCAAGCTCGAGGTCGCCTGCCCCGGCGTCGTCTCCTGCTCAGACGTCCTCGCGCTCGCTGCGCGCGACTCCGTCCGCATCACCGAAGGGCCGTACATCCCGATTCCGACGGGGAGGCAGGACGGCAACAGGTCCTCCGCCGCCGACGTGGCCCCCAACTCTCCTAAGCCGGGCGCCACCGTCACCGACCTCATCGCCCTCTTCGCCAGGTTCAACCTCACCGCCAAGGACCTCGCGGTCCTCTCCGGCGCGCACACCATCGGCAGGGCGCACTGCTCCGCCTTCTCGCCCCGCCTCTACAACTTCAACGGCAGCAGCAACGCCTCCTCCGATCCCACGCTCGACGCCAACTACACGGCGACTCTCCGCGGCCAGTGCAAGGCCGGCGACCTCGCCACCCTCGTCGACCTCGACCCCAGCAGCGGCACAACCTTCGACCTCGGATACTACAGGGACGTCGCGGCCAGCAAGGGCCTCCTCTCCACCGACGCCGCGCTGCTCCTAGACGCCGACACCAGGGCCTACGTGCTGCGACAGGCCAACGCCACCGCCAACCAAGAGTTCTTCGCCGACTTCGCCGTGTCCTTCGTCAACATGAGCAAGATCGGCGTGCTCACGCACCACAAGGGAGAGATTAGGAGGCAGTGCTCCGTCGTCAACCCGCCGTCGGCCCATTCCTCGGCGCCAATGACGACCATGAGCGCGCACGCCGTGCTCCTCGCGACCTCGCTGATTTCCGTGCTTGCTCTCGCCCTTGTGCTCTAGAAACTCTGCCGTGCATGCGTGGCACCGCAGCAACTTGTGCGATTCAGGATTTTGGTTTTGGTTTGTTTTTGGCATAATCGCTTAATGCCTTCAGTTTCCGACTACTTTGCTTGCAACTTTCCATTGATTTGTGATTGTAAGTACCTGATCCATGCATTCGTGTGCATCAGGGCATCATGTCATTTCTTGTGGGCATGCCCTGCCCTGAATAAACGAACCG

1. Gene ID: DN68939_c1_g4

CCTGGGTTGCGGGCGAGCGCGCGGCTGACGGCATCGCGCACGATGTCCTCCGCCTGCGGGCAGGTGTGCTTATAGAACCCAACCTTCAGAGACGACGACGGCGGGTACGACGCCACCACCACTGTTGAAGAGGCGGCCATGGCGAGCAGCACCGCCGCACAAAAGGCCAACCGTGGCGGCGGCATCGCGGTCTAAAGCTCGATTAATTACTAGCTTATTATTCGGTTGGGTCGGTGAAGTGCTAGCTAGCAAGCTCGATGTCGACCGGCTAATTGATGATGCTCGATAAGAAATTGATCGAATGGTGATGGAATTTGTTATGTGTGG

1. Gene ID: DN75075_c0_g3

CTTGTTGATGGTGTAGCTTTCGACCTCCGCTGCGCATTCCTGCTACGACCGCTCTAGCTAGCAACTTACATACGAAGAGTCGCGCTGTGTGTCGAGAAGGCCATGGCTGGAGCTGCAGGGGAGACGGTGTGCGTGACCGGCGCCGGAGGGTACATCGCGTCGTGGCTGGTGAAGCTCCTCCTCTCCCGCGGCTACACCGTCCACGGCACCGTCCGCGACCTCGGTGAGAAGAAGACCGGCCATCTGAGATCGTTAGAGAACGCTTCTGAAAATCTCAAGCTTTTCAAGGCCGATCTCCTTGATTATGAGGAGATGAAAGCTGCCATAGCAGGATGCCAGGGGGTTTTCCATGTGGCCTCCCCGGTTCCTTCAGCCAATCTGACGGATCCAGAGCTAGAAGTTGTGCGTCCTGCTGTCGATGGTACCAAAAATGTACTGGAGGCTGCATCTGCCGCCAAGGTTCGCCGAGTGGTTGTCGTGTCATCCATTGTTGCCGTGGATATCAACCCCAGAGATTGGCCCACAGACAAGATCAAAGATGAGAACAGCTGGTCAGACGTAGAATTTTGCAGGAATAATGAGGACTGGTATTCAGTTTCCAAGATCACAGCAGAAAAGGCGGCGCATGAATACGGAGCGAAGACCGGCTTGGATGTGGTCATTCTCAACCCAGCGGTGGTGTTTGGTCCCCTGCTGCAGCCAACGGTGAATGCCAGCAGCCAGTTCCTGATCTACTTCCTGAAAGGAGGACCTGACCAGATGAGGGACAAGCTCTGGCACATCGTCGACGTGCGAGACACTGCCGATGCGCTGCTTCTGCTCTACGAGGCGCCCGAGGCCTCTGGCAGACACATCTGCGCGCCACACATCATCACCGCCCGGAACCTGAGGGAGCTGCTGAAGAGGAAGTACCCTCAATATCCTCACATTAGCGAGGACAACATCAATGACATGGAGCACCCGGCGCCGATGACCTCCGACAAGCTGAAGAAGCTGGGGTGGGAGTTCCGATCGCTGGAGCAGACCATCGACGACACCGTCGAGTTCTGCCGTCAAGCCGGCGTTCTGGACGATGTGGCGGGGGCGGCGCCATGGCGTTTCCCTCCTGTTTACAACAAGATTTAGGCTCCGCTGGGCCAGCAATGATCATTCGATTCAATAAGGTGCCGACCACTTCAATTTTAAAACAATTCTCACAGTAGCCCACCGGCCGCTGCCCCTGTCAGTAGCATACATATGGTGTACTTTTGTGTAACGAAGCCATGAAAAAACATCAAAGTCAATAATTTCATTTGCTCCCACACGGTCCCACAAAAACATTCTTGGGCAG

1. Gene ID: DN54394_c0_g1

GTTCGAAACACGATCAAACTTGTGGGGTGTACAGCTTGCACAATCCTCAAAACTAATTCAAGGAAAGCTCAATCATAATAAAGCACGGGTCTAACACGAGATACCAGCTATATTTTTACTAAATGATTTATTCCCCTAATGCCTCCCCACGATAAGGTTCTGATCTGGATGCTTTAATGTATTCATTTGGCCTTCCGCAATAAAAAGGAATGTCTTCATCTCCTAAAGGTATTCTATCTGCCTCTCCACACATATAATCAATAAGTATATTTTGAAAACGATGAATCGTCTCCTCAAAGCGGAATGACATTCTACCTCCTTTGTATGCTTTGCACTTCACACCGTGTTGAACACTTTCACAGATTTCAATATCTTCCAGGTTTGTCATATCGTAAAAAGCCATAGCCGAATCCAGAGCTTTTTCTAACATCTCTCTTTTGTTTGTCTTGTTTGCTTCTTCTAGTAGCCTTGGATGAACCATCAAAGAAGCTTTTTCAATAGTGCGGTTTGGTCCATCAGGTACAAGATATACAACAAATAGCTGATTTGGCCACAGAAAATAAAATACATTTGGGAATAAGTGGTGAAAAACTGCAGTATATCTTTCCTCTTCAGTCAAACCAGGGAATGGTGGAAATATATGAGGATCAATTGGTGTACCTGCAGAAGTCAAAGGTCTGGTGACAAAGCCAATGTATTGGCCATGACCCTGCAAACGATGATGGTCGTCAACTCGTGAAACTTCAGTCAATGCAGGATGAACACTGGGTAGGTGATAGTATTCCATAAAGTTTTCCGCAAGAAGTTTCCAATTGGATTCCGAAGTATAAGTCTTGGAACGTACTGTGATGAGCTCCTCCAAACGATAGTTCTTCAATACATCAACAACATCACCATAAAGAGTTTGTAAAGATGTAGCATTTTTGTCAGCATTCGCAAACACCATGCATCCTAATGTTTCAACTTTCACTTCAAACAAGGAATAATCCTTTTTATCAA

1. Gene ID: DN67361_c0_g1

CCAGCCCTTGCACTTTGTTTAATCCTCATGCATGCAACCATGCATCCGGCCCGACTGAAATTCTAGGTCGCGCTGTTGGTGGCGAAGCAGACCTTCCTGACCTCGCCGGAAGCCCCAGTGAGAACGTCGATGCGGCCCATCTTCAACATGGCGCCCGCGAACCTAGCCATCCACGCGATGGGTCTGGCGGCGTTGTCGGCGACCATAGCCGCCGTGTCGTTGCGGCTCGTCAGCGTCTGGTCTGACGTGAACAACACCTGCCTAGATGCCACGTTCTGGTAGAACGCGTTGCTCAGCTTGTTCGGGTCCGGCGACACGCGGTTGTTGTTCACCAAGATCGATCCCCCGCCTTGCTGCGGGCACACGCTCCTCAGCGCCGTCGCGAAGGTGGCGTTCATGGTGGGGTCGGCGGGGTACAGCCTCGGTTTGACGAAGTTGCAGGTCGTTTGGCCGAAGGAGTGCGCGCCGGAGAGTGCCACGAGGTCGACGGCGTCAAGGCCCTTGGCGTCGAAGCTCTTGAGGAGGTCCTTGAGCTGGGACGACGGTGAAGGCAAGTGTTGGAACACCTCACCGAAGACGGAGACGAGACCGTCGCGGCGGCCGGAGGGCATGCCGAAGGTGAAGCCGCCGGAGGCGGTGGTGGAGTCGCGCGCCGCGAAGGCGAGGACGTCGGCGCAGGAGACGACTCCCGGGCAGATGGACTCTAGGGCGGCCTTGATCTTGTTCACAGCGTCGTATCCGCGCAGAGCGATTGCTTGCTTCTCCGTGTTCGGGTTGCTTGTGCTCGGATCGAGCAGAATGGACGCATCACAACCCCTGACAAAGCAGTCGTGGAAGAAGAGGCGAACGAAGGCGGCGCCCATTGTGGGGTCATTGCTGATCATGCCCTGGACGATGCCCCTCACCGTATCCTCCGCTTTCGGGCACGATGAATTGTAGAACTTGTACTGGAGCCCGCTGGCCATCGATACGGAGCTCAGTGTCGCCGCCATAAACGCCATGAGCACCACAATCTTGGATACTGTCGCACTCGACATGTTCACGTAGGTGCTGTTTGTGTATGCTTAAGAAAAGAGGCTTGAAG

1. Gene ID: DN99744_c0_g2

GCCACCAGATGGTGCCCTCCTCCTCGGACAAAAGAATCTCGTAAGTGAAATTGGTTCCTGGCTGGATGGGACACTGTGTTATGAACTCTGGCCCATCGTACCAAGGGTTCCGGGGTTGGTCCACGCCGTGCCAGTGGATGGTTATGTTCTTATCGCCTTGGTTGTGCACGTTCACGAAGACCCATTCGCCCCTCCGGGCCGTGATGGTCGGGCCGGGGAACTGGCCGTTGACGGTGAGCAAGGTCTTGTTCTTGCAGAGCCTTGAGTAGCTAGTCTCCTTTACGAAGAAATCATAGTAGTTGTGTCCTTGCTTGGCGTCCTGAGCGAGGCCAACTGCTGCCCCAAGCATGAAGACTGCAGCAAGTAACCAAAGCATCGCCTGCATCTTAGCTACACCCAACATTGCCTTGCCGGCCACAAGTTTCGACCGTCAGGTGGCACTAGGGAGCTGGTTTGCTTGGTAACACTTGCTT

1. Gene ID: DN93689_c0_g1

GGGGATCCAGATAGTACTGCGCACGAGAAAGTGCGAGTGTACTATTAAAAAATAATAATAACTCAAGCATAGAAAGCTTCGCCATGAATCTGATCAGCTAACCGATCTAGTTGGTGTTCAAGACACGGCAACTCCTCCTAATCTCGCCGTTAGCAATTGTCTTGACATCAATAGCACCCATCTTCACCATCGCTGCCTTGAACTTCTCTTCCCACTGGTTTGTATCCTTAGCGTTTGCACGCACCGCGTCGCTGGTGTCTGTCGCCGTCAATAGCGTGGCGTCAGAGTCAAAGAGCACCTCATGGCTGAGCACGTTCTTGTAGTACTGGTTGTCCATCTTGTCAGGGGTATTGATGTCCTGTACCACCGTGTTGTCGCTGCCATTGTTTGACCGACATTCCTCTTGCAGAGTGCTTTTCAGATTGGGGTCCATGTCGGAGGAGTTTGAGGTGAGGCGGTTTGAGAAGGATGAGCAATGGGAGATCCCTACGGTGTGCGCACCGGAGAGGGTTACCATCTCGTCAGCGGTGAGGCCTTTGGATGCAAACCCGGCCTTGAGCTGAGCCACGGTGGCGAAGGGAGGGGGGAGGTTGATGAGTGTCTCGTTTATGAGGGACACACGGCCATCATAGCGGCCGGCAGGCATGTCGAAGAAGACCTTGTTGTTGCTGAGGAAGTAGGTGGCGTCACGTCCGGCGAAGGCCACAATGTCGGCACATGACACGACGGTGCCGCACTCCTTCTCGATCTTGGCCTTTGCCGCGTCAATCACTTCAAAGCCACGCAGGCTCAGGTTTGGGATGCCAAACTTCTCCGTCGCACTGTTCGGGTCGGCCGTATCCAGCAACACGGATGCATCGCAACCCCTGACAAAGCAGTCATGAAAGAATAGACGGATGAGTCCCGCCATGATGCCCTTGCTGGCGTTGCGCACGGCTTCTCTCACGATGTCTTCCGCACGGTAGCATGTATTCTGGTAATAGCCAACGGCGAGCCCCGGTGCAGGTGAGCTCGGAGTGGGCGATGGAGGTGTATTCGGTGGTGGAGTAGATGGTGCAATCGGGGAGGGCGGAGCAGGACTAGGTGTAATCGGCGTCGGTGGTGGAGGAGGACTTGGTGTATTCGGGCTGGAAGGCACAGGGTTGGGATACCCGTAGCCTGCTTGGCACGGACGAGCCACGCAGGCGACCAGTGCGAGGACAGTCAGGGCGGCGGCGAGCTTTGCCATTGTCGCTGGGTTGCTCGCGCTGGTTGATCCCGGTGGTGTATCTTG

1. Gene ID: DN87778_c0_g2

ATGATACTATAATTATTTCCAGTAAAAGTGAACAACAACATCACCAATATGCTATACCTTCCCAAAGAATTTGAACTGTTGAAACGAACGAATTACATGCGTGAAATCACTCGGAAGAGACAAGCAGGTGAATAAAGAGCACAAACAAATTGGTTGCCGTGATGATTCATTAGTAGCGGTTGACGACCCTGCAGTTCCTCCTGATTTCCCCTTGGTTTCCGGTCTTGACTCCGATGGCACCCATCTTGACCATCGCCTTCTTGAACTTGTCCTCCCATAGTCCACGGATGTTGGCATTGTCAAGCACCATCTGGGTGGTCGCCGGTGTGGTAAGAAGGGCAGCGTCCGACGTGAACAAGACCTTGTGTGCGAGGACGTTCTTGTAGTACTGGTTGTCGAGCGCGTTGGGGGTTACCACGTCCTGGTGCACCGTGGGGTCGTTTGCTGTGGTCGGGTTGGCGGGGCATTGCCTTCTCAGGACGCCAGCGAGGGCGGTGTTGATGTCGGAGGGGACGGCGAGGCGGTCAGAGACGAAGGATGAGCAATGTGAGACCCCAATCGTATGGGCGCCGGAAAGAACCACCATGTCCTCTGTGTCAAGCCCTTTAGCGGCGAAGCTGCCGATGAGCTGAGTGATGTTAAAGAATGGAGGCGGCAGGTTGGAGAGGGCCTCAGTGAAATTGGAAATGCGCCCATCCAGGCGGCCTGCCGGCATGTTGATCTTGACCGTCATCCTGCTGAGGAAGTAGGCCGCATCACGGCTGGCGAAGGCAACGATGTCAGCGCATGATACCACGCCTGGGCATGCCTTTTCCACGGCGTCTTTGGCCGCGTCGATGGCCTCGAAGCCGCGGAGGCTGGGAAAGTTGGGAGGGCTGAGCTTCTCCGGCTGCGGGTTGGCCGGTGTTGGGTCCAGGAGGACAGATGCATCACATCCCTCGACGAAGCAGTCATGGAAGAACATGCGGATCAGCCCGGCGCCGATGCCGGCGTTCTTGTAGACGAACTTCTTCACCTCATCCCTCACGATGGCCTCCACGCGGGGGCACGACTTCTTGTAGTAGCCCACCTGCAGGCCATGGCACGTCGATGACAAGAGCAACGCGCATGTCAGCGCAATAGAAAGCCTAAGCGAAGCTGAAGCCATTGTCTTCTTCAGCTCTTAACAACAAGGCTTTGTGTACGAGAGAGTGTGGGCTAGAGAGTTATAATGAAGTGATG

1. Gene ID: DN92367_c1_g4

AGAGGGGCACGCCGTGACGGTGATGGAGCAGAGCGGCGACGTCGGCGGGCAGTGGCTGTACGACCCGAGGACCGACGGCGACGACCCGCTCGGCGCCGCGGTGCCGGTGCGCGTGCCCGGTAGCATCTACGCCTGCCTCCGTCTCATCAGTCCGCGAGAGGCTATGGGCTTCTCCGACTTCCAGTTCGTGCCCAGGGACGGGGACGACCGAGACCCGCGACGCTTCCCCGGCCACGGCGAGGTGCACTGCTACCTCCGGGACTTCTGCCACGCGTTCGGGCTCATGGACGCCGTCAGGCTCAACACCCGTGTCCTGCGCGTCGCAGCCGCGGCGCCGACGCCGACATTGACGACGCGTCAGTGGGCGGTGAGGTCGGTGGGCCTCGGTGGCGGCACCGGCACGGACGACGCGCAAGATGAGGTGTTCGACGCCGTGGTGGTGGCCACCGGCCACTACTCGCAGCCGATGCTCCCGCGCATCCAAGGCATGGAGGATTGGAGGCGTAGGCAGCTGCACAGCCACTCGTACCGGACGCCTGAGCCGTTCCAGGGAGAGACCGTGGTGGTGGTCGGGTGCGGTGACAGCGGCAAGGACATTGCGCTGGACCTCTGCAAGGTCGCCAGGGAGGTGCACCTCACCGCCAACTCCTCCTCTGTAGATGAAGCCACCACGCCTGCCATGTCCAGGATGCTGGCGAGCCACGAAGACAAGCTGCGCCTCCACCCGCGGATACGCCGGCTGCACGTCGACGGTCGTGTCGAGTTCGCGGACGGCTCCTCCGTACTCGCCGACACGGTCATCTACTGCACGGGGTACGGCTACTCGTTCTCGTTCCTGGACACGGGCGGGGCGGTGGCGGTCGAGGACGAGGTGGTCGGCCCGCTGTTCGAGCACGTGTTCCCGCCGTCGCTGGCGCCGTCGCTCTCGTTCGTGGGCGTGCCGAGGAAGATCCTGGTGCCGTGGTTCTTCGAGAGGCAAGCGAGGTGGATCGCGCAGGTGCTGTCCGGCCGCCGCGCGCTGCCGCCGGAGGAGGAGATGCTGCGGTCCGTGGAAGAGCACTACCGTGCCAGGGAGGCCGCCGGCGTGCCGAGGAAACTCACGCATAACATCGGCGGCGTCGAACCTCTGAAAATGTACGAGTTCGGGGAGAAGTACTGTGACTTCGCGCCGTTGGAGGAGTGGAAGAAGGAGCTGGTCCTATCCAGCATCTTGAGCATGAAGGAC

1. Gene ID: DN93689_c1_g2

GGAGACACACCACCGGGATCAGCACGAGCTAGGGGGCTACCCAGCGACAATGGCAAAGCTCACCGCCGCCCTGACGGTCCTCGCGCTGGTGGCCTGCGTGGGTCGTCCGTGCCAGGCAAGCTACGGGTATCCCTACCCCATGCCCTCGCCTAGTCCTCCGCCGCCGACTCCTCCACCATACACCCCGACCACACCTAGCCCACCTCCACCTGCCCCGATTACATCATCTAGTCCTCCACCGTACACCCCAACCACACCTAGCCCTCCGCCGCCCGCCCCGGTCACTCCATCCACTCCTCCGCCATACAGCCCAAGCACAGCTAGCCCTCCTCCGCCCACCCCAATTACCCCGTCTAGCCCACCACCGTACACCCCTAGCACACCTAGTCCTCCTCCTCCACCGCCCACTCCTAGCTCACCTGCCCCGAGCCTCACCGTTGGTTACTACCAGAAGACATGCTACCAAGCTGAAGGCATCGTGAGAGATGCGGTGCGCGACGCCAGCAAGGGCATCATGGCGGGGCTCATCCGTCTATTCTTCCATGACTGCTTTGTCAGGGGTTGCGACGCGTCTGTGTTGCTGGATACGGCTGACCCTAACAGCGCAACCGAGAAGTTTGGCATCCCAAACCTGAGTTTGCGCGGCTTTGAAGTGATCGACGCGGCCA

1. Gene ID: DN63605_c0_g1

CACACTGAAGTTTCCACTTTCAGTTTTTCAGTCTCCTGCTTGCCACACGCTCAGATCAAGTGCATCGAGCTCGAAGATGGCTATCAGGGTTGCGACCTTGGTGTCCTGTGCGCTGCTCCTGGCGGCGACATGGCAGGGTGCAGCGGCGGCGCCTCGGCTGAGGGTCGGGTACTACCAAAAGAAGTGCCCGGCGGCGGAGTATATCGTCAAGGGTGTCGTCGGCAAGGCTCTCCAACAGAACCCCGGCCTCGGCGCCGGCATCATCCGCATGGCCTTCCACGACTGCTTCGTCCAGGGCTGTGACGCGTCGGTGCTGCTGGACCCGACGGCGGCGAACCCTCGGCCGGAGAAGCTCGGCGCGCCCAACTTCCCCAGCCTGCGCGGCTTCGAGGTGATCGACGACGCTAAGGCGGCGCTCGAGAGGTTCTGCCCAGGCGTCGTCTCCTGCGCCGACGTCATCGCGTTCGCGGCCCGCGACTCCGCCTACTTCCTCAGCAACTACCGCA

1. Gene ID: DN76046_c0_g2

CAGCCAACCAGAAAAAGCGAGAGAGAAGCCACGGGCACCAACAAACATTTTTTACGCTGAACTTGTTGCCATGGCTTGTGCTTCTTGCCTTAGCTTACTGGTGCTGGTGGCAATGGCCTCGGTCGCGTCGGGGCAGCTGTCGTCAACGTTCTACGACACCTCGTGCCCCAACGCGCTGTCCACTATCAAGAGCGCCGTGACCGCCGCCGTGAGCAGCGAGGCCCGCATGGGGGCATCGCTAGTCCGGCTGCACTTCCACGACTGTTTTGTCGATGGATGTGACGGGTCAGTTCTGTTGGCGGACACGGGGAGCTTCATCGGCGAGCAGGGGGCAGCTCCCAACAATGGTTCCATTCGAGGCATGAATGTCATCGACAACATCAAGACGCAGGTGGAGGCCGTGTGCAACCAGACCGTCTCCTGCGCCGACATCCTCGCCGTCGCCGCCCGTGACTCCGTGGTCGCGCTTGGAGGGCCCTTTTGGACAGTTCCTCTGGGGAGGAGGGACTCCACCACCGCAAGCAAGACAAATGCAGAAAA

1. Gene ID: DN81688_c0_g2

CTTGGTGGAAACTTTACATGGCTGTAACGAAACAATGAATCAATGAAAACGAAACAATGGGGAGAGAAAAAGGAAGTAACCGTCTTACAGTAAAATCTACACAGAAAAAAGATTGTCGCTAATCAAACGCCATGCCATGCCAACCCGCGCTTGTCGGCTAAGTAGCTAGTGGTTCACCACTGAGCACTTCTTCCTGATCTCGCCCTGGCTTCCGGTGAGCACGTTGGCGTTGCCCATCTTTACCATGGAGACGCCGAAGTCGGCGAAGAATTCGTCCTTGAATGCGCCGGTGGCGTGGCGCAAGATGTAGGCGCGGGTGAAGGCGTTGGTGAGCAGCGCGCCGTCGGAGTGGAAGAGGCCCCTACGCTTGCTGACGAGCTTGAAGTAGTCGAGGTCGAAGGTCTTGAAGCTACCAGGGTCCATCTCCACGAGCGTGGTGTTGTCGTTGAGGCTGGCGCACTTGCTCTTGAGCTTCGCCATGTAGAAAGGCTCCAGCGTGGGGTCGATGTCACTGGGGTTGACCCTGCCGGTGAAGTTGTAGAGACGGTCGGAGAAGGAGAAGCAGTGCGACGTCCCGATCGTGTGCGCGGCGGAGAGGACGACGAGGTCCTTGATGTCAAGGTTCACTGCGGCAAAGAGCTGCGTGAGCTCGGTGATGTTGGCGGTTGGAGGGGGCAGCGCGTTGGTGTCGTTGGCGATGGACACGCTGCCGTCTCGCCGGCCGAGGGGAACTTCCCAGAATGGCCCCTTGCTCAGCCAGACTGCATCCCTGGCAATGAGCGCTAGCACGTCGGCGCATGAGACGGTGTCGGGGCAGGCCTTCTCCACGGCGGCCTTCACCCTCTCCACGAAGTCGAAACCACGCAGCGTTATGTTGGGCAGCGCGTCCTTCTCGGCCGTCTTGTTCGCCGAGTCCAGGAGAACGGAGCCGTCACAACCCCTGACGAAGCAGTCGTGGAAATGCATCCTGAGCAGCGGCGCGGCGAGGCTAGGCGCGAGGGAGAGCGCCCTCACCATCTCCTTCCGGACGACGTCCTCCACGCTGGGGCACGTCTCGCTGTAGAACTTCTCCTGCAGCTGCGCTGTCGCGCAGGACGCCGACGCCGCCGCGAGCAGCAGCGCCACCATCCAAGCCCTCGACGCCATCTCCACAAAGAAAGCAAGAAAGCTTTTGCTAGCTTCTTCCTTGGCTGCGCAAGGATGACGATGGCAAATCGGGGGAAGCTTGAACTGCTGCT

1. Gene ID: DN98601_c1_g1

GCAGTGCCAAGTTACAGTTTGCAAGCTTATCATTGATAACACATTTTGCGAAGACACGACGTGTTAAAACACATGGTGAACTGAAATGAAAGGTATCTCCAATCAGATCGAACTAGAGCGTGGAGCCGATATCAACCACGAAACGGTAGCGGACGTCGTTTAGTGCGAGCCTTGCCAGTGCCTCGTTGATCTTGTCAGCTGAGACAAGCTCGATGTCGCAGGTGATGTTGTGTTTCCCGCACAGGTCCATCATTTCCTGCGTCTCCTTCATGCCTCCCGTCAGGCTCCCGCTCACCGTCCTCTTTCCGAAGATAAGCGGTATTGACGGGAGTTCCACGGGCTTGTCAGGGGCACCGACGAGCACCAGCTTGCCGTTCACCTTGAGCAGCTCCAAGAGGGGTCCTAGCGAGTGTTGCGCCGACACCGTGTCGATGATATAGTCAAGGCTCCTCGCCATATCCTGCATCTGTTTCTGGTCGGTGCTGAGGACGAAGTCGTCTGCCCTAAGGCTCTCCCTGGCCTCGCGCTCTTTCGCCGGCGACGTGCTGACTATGGTGACGTGGAGCCCGAAGGCCTTGGCAAACTTGACGGCGACATGGCCGAGGCCACCCAGCCCGACCACCCCGAGCCTCCGTCCCGCCTCCCCCTGCAGCATACCGTGCTGCTTCATCGGGCTGTACACCGTGATCCCTGCGCACAGCAGCGGCGCCGCCGCGTCCAGCGGCAGGCTGTCCGGAATACTGACGATGAACCTCTTGTCCGCCACCAGCATATTGGAGTATCCGCCGTAGGTGATGCTGCCGTCCGAGGAGATGCTGTTGTAGGTGAACGTCATCTTATCGCAGTAGTTCTCCTCGGACCGGTGGCAGTGGTCGCAGTCGAGGCACGATGCAGCAATGCCTCCCACGCCGACGCGGTCGCCGGGATGGAAGCCGGACACGTTGGTGCCGACCTTGGTGACGACGCCGGTGATCTCGTGGCCGGGGACGATGGGGTACATGGTGATGCCCCACGCGTTCTTGATGAAGTGAAGGTCGGTGTGGCACATACCGCAGTACTGCACCTTGATGGTCACGTCGTCGACGCCGGTCTCACGCCGCTTGAAGGAGAAGGGCACGACCTCGCCGGAGGCATCCATGGCCGCCCACCCGCTCACGGCCTGCGTGTGGTTCGGGGTCACCTCCATCACAGCTATTTGGTGAATGTGGAGAGCGCGTGTCTTTGTATGAGATTCTTGCTGCCTTGGGTGCTGGTTGTGACTTG

1. Gene ID: DN84039_c0_g1

CTCACAAAACTCATTATTTCCAAACATATTCAGCAATGTCTCACAAGTATAATGGCGTATCTAGGTTCTAGGAGCACTGTACTACCACTACCGATTCATTCCTCAGCAGCATGAAAAAACTGAAACAGATCAGGAAGGCGGCAAGCTTGTCCCTCTCCCTCGACATCCTGCAGAATTCCTGTCTTCCGGTAGTATTCGACGCTATCCAGAAGCGTCTCCTCCAATGCCCTTGGCTTCCAGCCCTGATTCTTCAGTTTCTCTGACGAAATTGGTGTGATAGGAGAGTTCTGATCCGCATTATCCTTGCACTTCACGTAATTGTAGTTAGGGTACGCCTTCTTTAGCAACTCCAAGATAGCCTTTGTGTTGATGTAATTTGGTGCGGAGATATATCTCCCGGATGATTCTGGTTTCTCATACAGCAGAAGCAAAGCATCGGCCACATCACGCACATCGACTATATGCCACAGCCTGTCATTCATTGCATTGGGACCTCCTTTTATCACATAGACGAGGAGTTCGCTACTGGTATTGACAACAGGTTGCAGTTGTGGGCCAAAAACAATACAAGGACATACTGTAACAACATTAAGCCCATTCTTCTCGGCATATTCCAAGGCTGTCTCTTCAGCAACAGTTTTAGCAACACAATACCAAAGCTCATTTTCGATGCAAACTTTTTTGTCTGACCAGCAACTCTCATCTTTGGGTTTACCCTGAGGCCAGTTCGGGTTAAAGTGAACAGCAGAAGTGGATGACACCACCACAACTTTCTGAACCTTCATTGAGGAACAAACTTCAAGAATATTCAAGGTGCCCTTCACGGCAGGCTCCATTACTTCTGACTGAGGATCGACGATCTTATCGGCGGGCACAGGTGACGCGACATGGAAGACGCCCTCGCAGCCCTTGACGGCGGCGGCCAGCGCGGCGCGGTCGAGCACGTCGGCCTTGAACAGGCGCAGGCCCTCCGCCGCCCCGTCCAGCTGCGTTAGGTGCGCGTTCTTGGGATCGCATGGATCGCGGACGGTTGCGTGGACGGCGTAGCCCCGGGAGAGGAGCAGCTTGACGAGCCACGAGGCGATGTACCCGCCGCCCCCGGTCACGCACACCCGCGGCGGCGTCATCCTCTGCCTCTGCCTCTGCCTCTACCTCTACCAACGGGCTGGGCTCGGGCTGGCTCTCCGATCGTGCTGCGCCGTCGGCCTGGACTGTTCCCCTACGTACTCCTCGGTGACTCCGTCGCCTATATATCTCCGCCACACCTTTGCCTGCCGGCTAGCACCATCGGTTTGGTAGACTCCACGGCGCAAATCGACACGAAAAAATTGTGGCGATCGAGTAATTAAACAAAGACTCCCTCGAGCTCACGGTGTTCGTGCCACTGAACTGCCG

1. Gene ID: DN68939_c0_g1

GTTCAAGCTTCCCTCGATTAGCTACCTTGTCTAGCCACCGTCGTCCTTGCACAGCCAAGGAAGAAGCAAAGCTATTTGGCTTCTAACAATGGCGACGAGGGGTGTGATGGTTGCGCTGCTGCTCGCGGCGGTGGCGGCGTCGTGCGCGACGGCGCAGCTGCAGGAGAAGTTCTACAGTGAGTCGTGCCCCAGCGTGGAGGAGGTCGTCCGGAAGGAGATGGTCAGGGCGCTCTCCCTCGCGCCCAGCCTGGCCGCGCCGCTCCTCAGGATGCATTTCCACGACTGCTTCGTCAGGGGGTGCGACGGCTCGGTTCTTCTGGACTCGGCGAACAAGACGGCGGAGAAGGACGCGCTGCCCAACCAGACACTACGCGGCTTCGACTTCGTGGAGAGGGTGAAGGCCGCCGTGGAGAAGGCATGCCCCGACACCGTCTCCTGCGCAGACG

1. Gene ID: DN74633_c1_g4

GTCGTCCGTGATGGCGAACAGGGTCGGCAGTGGGCGGATGGCGCCCGCGGTGCTCCGCCTCTTCTTCCACGACTGCTTTGTCAACGGATGTGATGCCTCCGTTCTCCTCGACGCGACACCCTTCTCAGAGAGCGAGAAGGATGCCAAGCCAAACGCGTCCCTCACTGGCTTCACTGTGATCGATGAGATCAAAGCCGCACTCGAGAAAGAATGCCCCGCCACCGTCTCATGCGCGGACGTGCTCGCGCTCGCGTCTCGCGACGCGGTCACCCTCCTCGGTGGTCCCACCTGGAACATGCCTCTTGGCCGCAAGGACTCGCGCTTCGCCGCTGACAAGGAGTTCACGACGAAACACCTTCCCAGCCCGAACGACAACCTTGGCGAGCTCATCAAAATGTTCGGGGAGCTCGGCCTCGACGCCCGCGACATGACCGCACTCTCCGGTGCGCACACCGTCGGGATGGCCAACTGCGAGCACTACAGAGAACGCGTCTACGGCACCAGCGACACCGAGTACAACATCGACCCGTCCTTTGCGGAGGCCAGGCGGAAGATGTGCCCACCGCAAGGCTACTCAGGCGACGCAGGCAAGGCACCGTTCGACATTCAAACGCCCAGGAAGTTCGACAATGCTTACTACCGGGACCTTATCGCGCATCAGGGCCTCCTCAACTCCGACCAGGCTCTGTACAGCGGTGGAGGCGTGGACAGCCTCGTGGAGAGATATGGTGCCGACGGCGATGCTTTCGGGAGGGACTTCGCCAAGGCGATGGTAAAGATGGGGAACATTCTTCCGCTGAAGGGATTGCCGACAGAGGTGAGGCTCCACTGCTCCAAGGCAAACTATTGATGGCTCAGTACCAAGTGCTATCGGATTCTTTTATCAAATGTGCAGATTTTAACAAGTAATTAATTTATCTGCATCTTAATTTGTATATGTATTATTGATGTATCGACCAAAGTGTTGCCATAAATGTTCCAATACATGTTTGTAATAAATCGCGTTTTCCAGTTTGTTTTGAAGTGGAAAGAGCAATATTTTGTTTACGCTTTACATACATGGAACCATATGTATCCCATTAAGTATTT

1. Gene ID: DN71115_c0_g1

GCATCCATTATTTGTGGTTTTATTGCATCACACGATCATCACAATACATTTGTATTACATCTAGGAGTCAGATGTAACCGGCATCCACGGCCTTTTACAAACACATGACGGGTGGGAATGGGGATATATCACATCTATTTTTGCGCATCATATTGCTGTCTTGTGGCTGATGGTGAGTACGCTTGACAATCCGTCTTTAACAACTCCATGCATGTCATAGAACTCACGGTACGTGACGGCGTGGTAGCATGCCGGGCTATCCTTGCTAAGGAACTCCTCCGCGGGGCTGATGAGGCAATCCCACGTGGGTGTGATGAACACCCCCACTGATCTCCGGCTCTCCTTGGGGTTGGTCACCACCCTGTGCTCTATGCTCTTGAGCATCCCATTGGTCACAACCTGGAGCGGGAGGCCAAAGTTAACGATGTAGGCATTAGGCATGGTCTCCACGGTGAACCAGCTTCCTTTGTAAGAGAACTGGAGACCGGGAACGGTGCTAGGGATGAGAAGGCTGAGTAGGTTGCGGTCACAGTGCGGCGGTAGGCCAACTCCTTCAGGGTCTCCACACGGTGGGTACTGGTTCAGGGTGAGGTTCATATTGCCGCTGCCGAGATCACCCCCGAAATAGTCAGGCTGTAGCCCCATGCCCTCGCTCAGCAGCCGCAGCAGCTCCTCGCCCATGCGTCGCGTATGCACAGCGAACCTCTCGAAAACCTCCCGGAGCTTCTCGGGCTTCTCGGGCCAATCCTTCTTGCTGTCACCGATGGGAAAAGTCGACGAGAGGCGGAGACAGTCGAACCAGAACTTGGCGCCGCTGTTCTTGTAAGTGGATCCTGTGAAGAACCGGTTGGGTTTCAGATTGTCATCTGAGTGGAAGGACTCCCTGTCCGCGGCCGGCATCGCGAAGAACTCCTCGCACACTGCTTCCATGTCCCGCCTCACCTGCTCTGGGACGCCGTGGTTGATCACCTGGAAAAAACCGATGTCCTTGCCGGCGTCGAGGATGGCGCGGCTAATCTCTTCGCGGCTGAGGGAGAAGTCGACAACAGGCAGGGAGATGAAGCCCTCCGGGAGCGGTGCCTGCAGATTCCCCATGGAACAGTCGGTGAATATGATAGGCCGGGAGAAGAATTGGTAGTGATCGCTCGTTAGCTGTTGTTCTTT

1. Gene ID: DN86095_c0_g1

CGAGCACGGCAACACGGCCGTCTCGTGGCTCAGGGCGCTCTTCCACGACTGCACCGTGAAATCCTGCGACGCGTCGCTCCTCCTCGAGAGCAACGCCGCCACGGGCCTCGTCTCCGAGCAGTCCTCCACGAGGAGCTTCGGCATGCGGAACTTCAAGTACATCGACGACATCAAGTCGGCGCTCGAGAGCGCGTGCCCCGGCACCGTCTCCTGCGCCGACCTGCTCGCCCTCGCCGCCCGCGACGGCGTCGCCATGCTCGGCGGCCCCGCGGACATCCCGATGCGCACGGGGCGGAGGGACGCCACGGTGAGCTACTACGGCGAGGTGGAGCGGTACCTCCCCGTCCACAACGACACCGTGTCGGCGGTGCTGTCCCGGTTCGCGCCCATGGGCGTGGACGCCGAGGGCGTCGTGGCGCTGCTGGGCGCGCACTCCGTGGGACGCGTCCACTGCTTCAACCTCGTCGCCAGGCTCTACCCGGCGGTGGACGGCACCATCGAGCCGGCATACGGCACGTACCTCCGAGAGCGGTGCCCGACGGCCGACGCCGTTGAGGACACCCGCGACGTGGCGTACGCGCGGAACGACAGGGGCACGCCCATGGTGATCGACAACATGTACCACAAGAACCTGCTCAAAGGGAGGGGCCTCCTGCTGGTGGACCAGAGGCTCGCCACCGACCCGCGCACCGCGCCGTACGTGAGGAAGATGGCGGCGGACAACACATACTTTCACGACGTTTTCGCGGCGGCGTTGGTCAAGATGTCGGAGAACGGACCGCTCACCGGCGACAAGGGGGAGGTCAGGAAGGACTGCAAGTTCGTCAACTCTTAGTGAGTTCGTGGATTGGATTTTGGGTATGTGTGTGACTGTGAACTACTAAACGTAATCGTATACTCTACGTAATACGTCTATGTGTAATGCTGGCCGGGGCAATAATTTGGAACAAATGAAAGGATTACTGTATTCGTACGTGTAGGAGGGGCCAAGTGAAGACACTGCCGACATTTTGTCCTTTACTACGTACGAGTACCTGTGGATGAAGTGAAGATAATAATACGTAAGGG

1. Gene ID: DN71437_c0_g1

GCGCGGCTTCGAGGTGATCGACGACGCTAAGGCGGCGCTCGAGAGGTTCTGCCCAGGCGTCGTCTCCTGCGCCGACGTCATCGCGTTCGCGGCCCGCGACTCCGCCTACTTCCTCAGCAACTACCGCATCAACTACCAAATTCCATCCGGACGCTTCGACGGGAGCATCTCGCTCGAGAGCGACACGTTCGCCTTCCTCCCCCCGCCATCCTTCAACCACTCGCAGCTCGTCGACAGCTTCAAGGCCAAGAATATGAACGAGGACGACCTCGTGGTGCTCTCGGGCGCGCACTCCATCGGCCTCTCGCACTGCTCCTCCTTCACCGACCGCCTCCCCCCGAACCCCTCCACCATCAACCCCGCTCTCTCCACCCTGCTGCAGAGCAAGTGCCCGGTGAGCCCAAATTTCACCAATGACCCCACGGTGGATCAGGACATGGTGACACCAAACCTGTTGGACAACCAGTACTACAAGAACATACGGAAACGCAATGTTCTCTTCACCTCTGACGCGGCGCTGGTAACATCGCCCCTGTCGGCGAGGAAGGTGTATCAGAACGCTTTGTTCCCCGAGGTGTGGGAAAAGAAGTTCGAAAGGGCGATGGTCAAGATGTCAGCTATCGAGCTCAAGACCGCTGCCAACGGCGAGATCAGAAAGAATTGCAGGGTCGTCAACAATTAGGTTTGCTGGGTGATCACGAATCTGCAGTTACATTTGAATGTGTGTGCACTCACTGTTTCCTTATAATTCATTCGTTGTATTTAATTTGCTATTAGTTTCCTTGGGGCATGCTGTTTCACTTCTATTGGTGCATTCAGCTGATCATGTTGTAATGCTATATTCATAGAGTTGTGTTTTTGTAATAGGTGACACGTGAC

1. Gene ID: DN88338_c2_g1

ATTCAAAACATGAGTTCTAGCTTCCCCCAAAACCTTCCAATTATGGAGCTTCTTGCGCTCCTTGCCCTTCTCCTCTGCCTAGCAGTCTTCCGGCGTAGCTACAGTAGGCGTGGCCGTACTCCAGCGGCCCTGAAGCAGATCGTCGACCCTACCATCGCCCACCGCGCACTTGTTCAGAATGCCAATGCCTTCTCGACCAAACCGCAAGGCGTCTTCCCCGTAGCTCTGAACATGAGCTCCTTGCCATACGGCCCGAATTGGCGCGCGCTCCGGTGCAACATCACTGCCGAGACCCTTCACCCGTCGCACATTGGCGACCTGGCACCGTTGCAACGGGACGCCATCCAGTCCCTCATAGCTGGTTTATCCGGCCACGCCCCGGGTGAGGTGGTTGTGCGTGATCACCTTCACGCCGCCGTGTTCGGCCTGGTGGCGCGCGTCTGCTTCGGCGAGGGCGTCGATGAAGGCCACGTGAGCGCCATGCTGCGGCTGATGCGGGAATTCGTCATCGCCGCCGGGGAGCTGAACGGCATATTCGTCGGCTCGAGGCTGGCCAGGTTCGTCCACCGGAGGCGTCTGCGGCATATGTTGGCCTTCCGTGGCCGGCAAGCCGAGCTGTACCTCCCTCTCATAGCGGCACGACGGGAGTCTCGGCTTTGCGATGGCGGCGTCCGGCGTCCTTACGTGGACTCGCTCATCGATCTCCGCGTCCCTAATGGCGGCGACACGGGCAACAACGATCACATCAGCGGCCGGCGCGCTCTTAGGGACGACGAGCTGGTGAGCCTCGTGTCGGAGTTCCTGGGAGGAGGCACGGATACGATCGTGGCCATCGTTGAATGGACGCTCGCTCACCTCGTCACCCAGCCGGAGGTTCAGAACAAGCTGCGTCGTGAGGTTATTGACTCCGAGATGGAGGACATTGTCTCGGACAAGATCTTCCGCGATATGCCATACCTGCACGCGGTTGTGCTCGAGAGCCTCCGCATGCACCCATCAGTGCCGTTCATCCCCCGCAGGGTCCGTGCCGAGGACGCGGCGGTGCTGGGTGAAACGACCTTGACTGCGAGCGACTTCAGCGTGCAGTTTGTTGTGGGGGACATCGGGAGGGACAGTAGGACATGGACGAATCCCGACGAGTTCCTTCCTGAGCGGTTTTTGGCCGGAGGCGAGGCAGAGGGCGTAGGACCATCACCGGGTCCGAAACAGATCAGGATGATGCCATTCGGCGCGGGGCATAGGTTCTGCCCTGGCATGGGTGTGGCGATGGTGAACATCAAGTGCATCTTGGCTGCGCTCCTGCGCGAGTTCGAGTGGGAGCTACCGGCGCAGGACACCAGGGGCGTCGACATGACTGACCTCAACGTCTTCTTCAAGGTCATGAAAAAGCCACTGTTTGCGCGTGTCACACGACGCGCAGATACCACCTAGATTCTGATGAAGTTGAAGTTGTGTCTCCCACTCTCGCACATGTTTTCATCAGTTGGGGAGTGTTGACTTGGGG

1. Gene ID: DN92728_c1_g2

CGCCTCTCCGCCAACTACACCCAAAAAGTTGACGACCCACATCTAGCTGAAGATTATGGCCACCGCGGGAATCAAGCCATCCACCATGCGCGCTGTCCAGTACAGCCGCTACGGTGGCGGCGGGGCGTCTCTGAAGGTGACCTTAGTTTGTACGACTAGTTTGTTTCTATCTTATAGCTCTTTCAGAGTGATGATGTTGGTAACCCCTTTTTGTGGATCATCATGCGCAGTTTCTGCACAATTTTTTTTTCCCCCAAATAAGGCCTAAGCATTTCTTGATGCTCATCTATTTTTATCATGTGGAGCCATACGGACATGTATTAATTGTTGGTTTTGCTACGGCGATGAAATTTGCGAATTAATCAGAGATTCAGGTCAGGGTTGTGTTAGATGGTTGCTTCCAAGAAGAAACTGGTCGTGAAAGGGGATGTGGGGTGACAGCCTGGAAATGCAAAGAAAAAAAAAGTTGACTTTCATAATATTATATATGTACAATGTCCATTACGTATTTTTGTGCCTCCAGCTAACTTGTGGAATTTAATAATTTGTAAGGTCCCATTTTGCAACTCTTAGAAGCAATAGTTTTGGTTTAACACAACAATTGGCTATTTTTTTTGCGTTTTGCTGAAACAATTGGATGGAAATTGAGTTAGTTAAAGTGCTATGACTTATGAATACCCATCCTTTTGCTGAAACAAAATTAGGTCAGAAAAATTTAATAAAAAAGTGTATCTTTACTTCAGTTTGTGGAGATCCCCGTGCCGTCACTGAAGAAGGATCAAGTTCTTATAAAAGTAGAAGCGGCAAGCCTGAATCCGGCGGATTCTAAGGTTCAGCGAGGGCTGTTGCGTCCTTTTGTCCCAAGATTTCCATTTATTCCAGGTATTGATTCATTTGAACCACAGACTAGAGTTATTAAACAGAAGCTAATTTACCTGTATACCATGCGTTAGCTCCTGCACAGTAGATGTTTTGAATGCTACAAACAGAATGATGCATGAGTCACTTGAGGAAATGCACAATTTGTTCTTGATTATCAAAATGACTGTGCCTTGTGAAACAGTTACTGATGTAGCTGGAGAGATCGCGGAGGTCGGCTCTGCAGTAAGCGAGTTCAAAGTTGGTGATAAAGTTGTGGCTGCACTGAAATTCTGGGTAACTTCAAACAGGCAGATCTTTATCGTTCATTCAGTTTAAAATGAACATGCTCTGCATCATTCATGAGAGAGGATTTGAAAATTGCAGAAAGCTGGTGGACTCGCCGAGTATGTAGCAGCATCCGTGAGCCTCACCGTGCTACGCCCGGACGGCGTGTCAGCCGCCGAAGCCGCCGGGCTGCCCATGGCCGGTCTCACGGCTCTCCAGGCCCTGAAAGTGATTGGGACACAGTTCAACGGCACGGGCATCGGCAGCAACATCCTGATCACGGCGGCGTCCGGTGGCACCGGCACTTACGCCGTCCAGCTCGCCAAGCTCGGGAACCACCACGTCACGGCCACCTGCGGCGCGCGCAACGTGGAGCTCGTCGCGAGCATCGGCGCTGACGAGGTGCTCGACTACAAGACCCCCGAAGGCGCCGCACTAAAGAACTCCTCCGGCAAGAAGTATGACTACATCGTCAACGGCACGAAAGACGGCAAGTGGTCGGCGTTCAAGCCTAGCCTCAGCAGCCGTGGCAAAGTCGTGGACTTGGCTCCCAACTTTGGAAACGTTATTGCGTGGATTCTGACGAAGTTCACCAAGAAGAAAATGTCCGTTTTCCTGATGTCTCTGACGATGGGGGAACTGAGGTTTCTGCTTGAGCTGGTGAAGGAAGGGAAGCTCAAGACGGTGTTCGACTCACGGCATCCATTCGAGAAGGCGGCGGACGCATGGGACAAGTGCTTGAGCGGCCACGCGACGGGGAAGGTTATAGTTGAGATTTGATCATGTGAGGTGCAATGTTCGTTCCTGCATTTGTCTCCGACATGTGAGTGTCACGGATGTACTAAGTTTATACTTCAGTGTTGATTTCTGTTGTTATGGCTGTCATGTACTTTATTGCTTTTGTACTGTATACGGAAAACAAGGA

1. Gene ID: DN79687_c0_g5

AGTCGCGCGACTACATAAGATCACTACACCCATCGCAGATCATAGGAATCGGAAATGGAAGCTTCAAGTACCATACCATGGCTTCTCTGCATCTCAGTCGCCACTTTCGTCTTCTACAAGGTCTGCTATTCAGGTGAAGACAGATCGCAGGGTAGCTCGACCAACTCGCGGCTGCCGCCGGGACCGACACCCATCCCTCTCCTCGGCAACATCTTCCACCTCTCAGGCGAGCTGCACCACGCACTGGCAAGGCTCGCCGGAGTACACGGCCCCGTCTTCTCATTGAAGCTCGGCGCCACCACCTGCGTCGTCGCCTCCTCCTCCTCGTGCGCCCGCGACGTCCTGCAGAGGCACGACCAGATCCTGTCGGCGCCTTCCGTCACCGACGCCGCGCGCGCGCTCGGCAACCACGAGCACTCCTTCATCTGGCTGCCGTCCACCAGCCCGCTCTGGAGGCGCCTCCGCGCCCTCTCCACCACCCACCTCTTCTCCCCGCACGGCCTCGAGGCCACGCGGGCCGTGCGGGAGGCCAAGGTGAGGGAGCTGGTCGGCTTCCTCCGCGGTCACGCCGGCGAGGCCGTGCACGTCGGCCGCGTCGTGCGGTCCGGGATGCTCAACCTCGTGTCCAACGTGCTGCTCTCCCAGGACGTGGCCGACCTGACCTCGGACGTCGGCCAGGTGCAGGAGCTGGAGACGATGATCAGGGACATCCTTGACGAGCTCACCAAGCCCAACCTGTCGGACCTCTTCCCGGCGCTCGCCTCGCTGGACTTGCAGGGCCGCCGGCGGCGGACAGCCAAGCGTATCACCAGGTTCTTCGACTTCTTTGACCCGATCATCCAACGTCGTCTCAAAGATGGAGGCGAAAGGAAGGAGGATTTCCTAGATGTGCTGCTGCAGCTCCACTCGGCGGATCAGCTCAGCATTGAAACAATCAAGTCCTTTCTTTTGGATCTCTT

1. Gene ID: DN85257_c1_g1

CGTTGAAACCAGAAATTTTATTATGACAAGGGAAGTACCGACAGGAAACAGACATTTGCTGAAAGTTGACAAAAACGAGAGGAAGAAGGCTGCAGTCAGCCCGGATCAACCGAAATGAAGCACACAAAAGCCACAGGATGTCCTAGTCGTAGCTAGCTACTAGCTAAGCTCGCAGGGAAACAAACATGCACGGGCGTGGCGTCATAATCGGTGGGATCTCAAGCAGAGGCGGTGAGCCATCGCTGGTTGGACCAGAAGCAACTGTAGTTGCGGATCTCGCCATACTGTGCGGCTTCACTACTGGGCAAATGCGCCATCTTGCTCATGGAGGTACCGAACTGCGCAAAGAACCACCCTTGGTCCTGCGCAAAGTTGTTGACCCACCAGCGGGTGGTGTGGTTGCGGAGGAGCTGCAGGTCGGAGGTGAGCACCCCCTTCCCGGCCAGCAGGTTGTGGTAGTACTCATTGTCGAACCTGTCCGGGGTGGTCACGTCGAGGTCCTGCAGCCGGTTGGCGTCGCTGCTGCAGTTGGCGCGGAGCCGGCGAGCGAAGCCAGTGCTCTCCTGGAAGCGGTCCTGGAAGCCGCCGCAGCTGGACCTTCCGATGGTGTGCGCCCCAGAGAGCACCACCACGTCGGCGACGTCAAAGCCGCGGGTCGCGAACCTCTGGAGCAGCTCGGCGGCGCTCAAGTCGGGCCCCGGCAACTCCCAGATTCTGGCGAGGTCAGCGGGGGCTAGGCTGTCTTTCAGACCGAGCGGCACAGCGTAGCCTGGCACGTTGAAGTGCATCACGGCCTCGCGGGTGGCGAGGTTCATGATGTCGGTGCAGGAGACGGTGGGACCGCACGCGTCTGGGTTCTGCACCTTGGCGCGGATGGTGTCGATGAGAGCCATGGCGGTTCGCCTTATCCCCTTGTTCTGGGGCCAGCTCGTCTCGCTCCAGTACCTTCCATGGACTAGAAGCGAAGCGTCGCAGCCTTGCGGGAAGCAGTCGTGGAACATGATGCGGAGGAGACCGGCGGCGATGCCGATGTCCTCCTTCAGCGCCTCCTCCACGGAGTCGTGCACCATCTGCCGCAGTTTCGGGCACGACTCATCGTGGAAGTCATAGGACGGGCCGGCGGCTTTAGGCATCGGGAACATCAGCGCCGCAACTAGGCCTATCACCCCCAACATTGCTGCCCTGCTCCACCCCATTGATCCTACCTTGCTTAGCTCTCGTTTCTCTATGGCCAGCCAGCCTACTCTGTGTCGTACTAGATGTCACTCTGTG

1. Gene ID: DN100686_c1_g5

ATGAAGCTCCAGGGTTGGCTGATTCCTGGGGCGAGAAGTTTGAGGACCTGTACAAGAAATATGAGAGAGAAGGCAAGGCAAAGAAAGTTATTCCAGCACAGTCCCTGTGGTTTGACATTTTGAAGGCACAGATAGAGACTGGAACACCTTATATGCTCTATAAGGATACATGCAATAGGAAAAGTAACCAGCAGAATCTGGGCACAATCAAGTCTTCAAACTTGTGCACTGAGATAATTGAGTTCACAAGTCCTGAGGAAACTGCTGTCTGCAACCTAGCGTCTATTGCTTTACCACGTTTTGTAAGGGAAAAGGGTGTCCCTATAGAGTCCCATCCATCTAAGCTTGTTGGTAGCAATGGATCACAAAATAGATACTTTGACTTTGACAAGCTGGGCGAGGTAACTTCAACTATTACTTACAATCTAAACAAGATAATCGATGTGAATTATTATCCTGTTGAAACCGCAAAGAGGTCCAATATGCGACACAGGCCAATTGGCATAGGTGTTCAGGGCTTGGCAGACACTTTCATGTTACTTGGCATGCCATTTGATTCAGCTGAGGCTCAACAGTTGAATAGGGATATTTTTGAAACTATTTACTATCATGCTCTGAAAGCTTCTGCTGAGCTGGCTGCTAAAGAAGGTCCTTATGAAAC

1. Gene ID: DN83694_c1_g1

CGCTCACTCACAAACTGACACAGCCATAGTTGCTCGTAGTAGTAAGCAAAGTAGTAGCAATGGCAGTGTTGAGCAGGGCGGCAATGCTGGCCACGATGGGCCTCGTGGCAGCGCTGCTGTTCCCGGCAGCCATGTCGATGCGGCCGTCCTTGATCTCGATGACCACCGGCGGCAACCGGCAACTCCCCGAGGGCCTGTCCTATGACTTCTACAATGATACGTGCCCCAAACTGGAGATCATGGTGCGCGAGGCCGTCGAGAGGGCGATTGCGAGCGATGTTGGGGTCGTCCCTGGCTTGCTCCGAATCTTCTTCCACGACTGCTTCCCTCAGGGCTGCGATGGGTCGATTCTACTGACCGGAGCAAACAGCGAGCAGAAGATGCCCCAGAACGCGGGGCTGCGCCAGAGCGCGCTGGACCTCATCGAGAGCATCCGCGAAACGGTGCACGAGGAATGCGGGGCGATCGTGTCGTGCGCCGACATCTCCAACCTCGCAACCAAGCACGCCGTCATGCAGTCCGGCGTGCCAGGGAACCTGGTGCCAGGGTACCGACTGCCCCTCGGCCGCAAGGACAGCCTCGGGCCCGCTACAACCCAGCAAGTCAGGACCATTCCCAGCCCCGACCTCGCCGTCAACCAGCTCGTCGAAGCCTTTGCCCTCCGCGGCTTGAATGAGGTCGATCTCGTCGCGCTCTCCGGCGCGCACACCATCGGCAAGGCGAGCTGCCCCAGCTTCATGAACCGCAAAGACGAGAACGACGAGTTCGTGCTCAAACTCAAGAGCAACTGTACTAGGTTCCCTGCCGCCCCACTCCAGGACCTCGACGTTACCACCCCTAACACCTTCGACAACAACTACTACCTCAACCTCCAGAAGAAGATGGGCGTGCTCAACTCCGACATGCAGCTCACCCTCAACGCCACCATAAACCAGTTGGTCGACTTCTTCGCCGCGGACCAGGGGTGGTTCTTTTCGACTTTCAGCACCTCCATGAGCAATCTCGCACATCTGGAAGGCAAACCAGCCATAATAGGGGAGGTCCGCCGCAACTGCTTCAAGGTTAATGGGCTGGAACTCACCGCCCCTAACACCTTCGTCGCCTCTGCTTAAGGCCTCTTATGGCATGCCGCCATGCCATGCATCTCATGAAATAAGAGCTAGCTAGCTATCTACCTAGCCTAATTATCTTTTTCTTCACTCTTATTACAATCCGATCGTATGCCCCACCTAGCCAGTCCTAGAGCCACTCGTCGGGCCTGTACCATTTCCTTTCAATAACTTTGTCAATTTCCTGC

1. Gene ID: DN90429_c1_g3

TGTGTATAGGCCTCTCACAGAGATGTATGAAGCCGCAGCCGATGCTGATGTAGTGTTCACAAGCACCTCATCTGAATCACCATTGTTCATGAAGGAACACGTTGGCACTGGTTTCACTGGTGTTGCGCCGCACTACACTCCTCCACCAACAACCTTCACGTGCTTCTTGGCACCTACTGGTTCGATAACCTTGGTTTCTTTCTGAGGGAAAACTTACTGCTGTGCGCATCATACCTTCCCTTGGGGTTCCCAACGGACGTGTACATCTACGCGCAAGCACCTGGCTGTTTTGGTGACTCTCCCTGGCCTTGTGTGTTGTTACACATGCAGCAGTCCTTGTGTGCTGCCTGTGTGTGTGTGGCTTGGGACTTGGGCTGGAATGGATCAGCTCGGCTGATCCCTGGTCATATCCTCTTCATTTCAATTGTTTGCTAACCTGCCAAGTGGCTTTGCCACTTGGCATAACTATTTCTTTGTTGTGTTTGTGTGCAGGTTTCAAGATGATGATCAAATGAAGATGAAGATCATCAAACTCAAGCTTAAATGAAGATTAGCTTGTAGTTTTAGGATAGATCAATACATTGTACTTTTCTTTTCTTTTCTTATTCTATTTTGCCAATTCTTGTAATGTGTTGTAATATTCCTTTATTTCAATTGTGAATATTGTAAAGACAATGTATA

1. Gene ID: DN86126_c0_g1

GCAGTTCTGATTTCTAAAGTGGATACAGCGTTCAAATGATGTGAGACTGCAGTTGAACTGATACTAACACAGATAACTGCAGTTTGATACTATGAGATACTACATCACCAAGCGAGTACCGTTGAAAGAGTTACACAAATGGTGCTAATTCGTCATGGTAGAAGAACACACCAGTGGGTCCCCCCACCGGTAAGAGGGACACCATGACAATGCTACCGGCACCTTCCTCCGCCGTCAGTTCTCCGGTGTCGTAGTTGATGTCAGTCATGCAATAGCCAGGGTGCACGCTGTTGATGCGCAACGCGGGGAAATTCTTTGCCATAATACGAGTGTATCCGTTGATCAGGGCCTTGGACACTTTGTAGGCTAAGTACTCTGCATCGGCAGGCCATCCATGAGATTTCAGCTGGCCATTCTTGTAGTCATTCAGGAACAGTCTTGACATCTCATCTAGCCTCTGTATTGTCAGGTTTTCGACGTTGTTCAGTTCCAGCTTGACGTCCTCACCGCTGAAATACCTCAGTAATCCGTAGTTGGACGACACGTTGACAAGTCTTCCATCAGAGGAGGCCTGAAGGAGAGGGAGCAGTGCTTCCGTCACATATTTGGTGCCGAAGTAGTTTATTCTCAAGCATTCTTCGGCTTCCTCGTAAGTTTCCGTGGAGTGCTGTAGCAACCACTGGAGCCTCTCCGTTGCATTCTTGCCTGCAAGCTATTTGATGCATCCACATGGTCTAGTATGTTAGTTAAGCTTCAGAACAAAACTAAGAATAAGGAAAAGTACAGACCGTTTTTGTAACTTGGCTGACTTGGTTCACTTTATTTTTCGAGTTGAAGGAGCAGATAACTACTGTTAAGCTTTCTTCTAAAGAAGAAGAACAAAATGTTAAGTGCAAAGTCTTACAGGTCAATGGGCCTACCACAACCTGAAGGGGTGTCGTGGTGTCGATCTCTGCAGTTGCACCTATAACACCTGCATTGTTGATCTGCAAAGCTTACAGGATTCAAAAATCCATATGATAAGCAACCAACAGAGACGGTACCCATGCTTAGTTAAGAATATTGGAATATCCACAGGTTTTTGTTCAGGAAAACATTATTATCTATGGCCTCTGCATCTATATGACGCGTGAAAAAAAAATACCGTACCAGTATATCTAGCTTGCCGAACTTGTTCTCGATAAAATCAGCCAAACAGGCAGCGCTGGACGGATCACTCACATCCAAGTTGTGATACACCACATCGGAGAACCCCGACGCGTGAAGCGCGCGCGCTGCTTCGGTGCCCCTTTTCTCGTCCCTCGCCGTTAGAACGACGACAACCTCCTTGGAGGCCAGCTGCCTGCATACCTCGAGCCCGATTCCTTTGTTTCCTCCGGTCACAACAGCTATCCTTCTCTCAGAGGTGGCGCTCTTTTCCATTTTGAAGAGCTTCTGCTTCAACATAAAAGCTTAGAGGGGAGATCCGGATGTGCGAATTGTCCTAGATCGATCATTAGCATTCGTCCGGATCGCAATTTATAATAAGGGAATGAGAGGCCCGGCGCTGTAAAAAAAAATGCAAGGTTGGCTCTAGTGTGTGAACTGCGAGAAACGCTTCGGCCCGTCCCTGTCGCTCCCCGCGGGCGACTGGGCGGGCGATTCCAACCCTAGCCCAGCAACTCCTCGCCCCTCCCCCTTCCGCCGCCGCAGCCGGCGTTAGCCGCCGGCCAAAGGCCGTGCGGTGCCGACGGGGGCGGATCCCGGGATCCCACCCGCGAGGCGGAGGAGCGGGGCTCTCCCATATTGGCGGCGGAGATCTGCAGCGATGAGCCGGCGCGCCGCGACGAGACGGCTCCTCGTGGTGGAGGCGGCGGAGCTCGACGACGCTGGCTTCTTCCTCGCGGGAGACGACAGCGGCGGCGCAGCTTCTTGTGGCAGGGCATCTTAGGCGACGGGGCGGCGGCCTCGGGTGGAGTGGCGGAATCTGCTATGGCGGCTAGCGGCGTCCGTTGGTGCGCGCCGTCGTCCTTGCAAGAGCTTAAACATGAGCACAACAATGGGTGCAAAGCATATGATGCAAGAGCTTAAACATGAGCACAACAATTGCCAAGTATCACATTACCCAAGACATTAATAGCAATTACTACATGTATCATTTTCCAATTCCAACCATATAACAATTTAACGAAGGAGAAACTTCGCCATGAATACTATGAGTAGAAACCA

1. Gene ID: DN76086_c0_g1

GCTATCTTTGAGAAATAGATGGGTGTGATGTTGTGGATGCATCTAGCTTCCACAGGCCAAGCACTCTTCCCGGTTGTTCAAGGAACAGACGACCTGTGCCATTTTGGCCTCATCATCTTCCTCTGCTGGTTTGACATCAGTGTCCCCATTCACCTTCAAAAAGCCTGTATCCACTGTAAACTTGATGGCATCAGCGGCTGCACGTGTTCTTAAATAGTACATCCCAGTTTTCAGGCCCTTGGACCAAGCATGGAAATGCAATGAAGTTAGCTTCGCAAAGTTTGGTTGGTCCATGTGAACATTAAGGCTCTGACTCTGATCAATGTAGCATCCACGATCAACAGCCATGTCAACCAACGTTTTCTGCTTGATCTCCCAAACAGTTTTGTAGATTGCCTTAAGATCATCAGGGATTTCTGTCATCTTTTGAACAGAACCATCTTCATAAATAATCTTGTTTTTCAGAGCAGGTGTCCAAATGCCCATCTCAGTCAAATCATGGAGAAGATGCTTGTTAACCACCACAAACTCTCCACTTAAAACCCGTCGACTGTATATGTTAGATGTGTAAGGCTCAAAGCACTCATTGTTGCCAAGAATCTGACTAGTGGAAGCAGTGGGCATTGGAGCAACAAGAAGAGAATTTCTCACTCCAACTTTAGAAATAGTCTCCCTTAAAGCTGGCCAGTTCCATCTGCTCGATGGCACTACATTCCACATGTCAGGTTGGATAATGCCCTTGCTGACAGGGCACCCTTGATATGTTTCATAAGGACCTTCTTTAGCAGCCAGTTCAGCAGAAGCTTTCAGAGCATGATAGTAAATAGTTTCAAAAATATCCCTATTCAACTGCTGAGCCTCAGCTGAATCAAATGGCATGCCAAGTAACATGAAAGTGTCTGCCAA

1. Gene ID: DN100686_c1_g3

GAGCCCCCAAACCCTACTACGGGAGTACCAGCACCACCACCAACCACCCCCCATTCCTAACCCAATCCACACCGCGGCGGCGGCGGCGGCGCGCACCACCTGCTCCTCGACGGAAGGCCCCTCTCCCACGCGAGTCGGCCGAGATGTACGTCGTCAAGCGCGACGGGCGCACGGAGACGGTGCACTTCGACAAGATCACGGCGCGCCTCAAGAAGCTCAGCTACGGCCTCAGCCAGGACCACTGCGACCCCGTACTCGTCGCGCAGAAGGTGTGCGCCGGGGTCTACAAGGGCGTCACCACCAGCCAGCTCGACGAGCTCGCCGCCGAGACCGCAGCAGCGCTCACGGCATCGCACCCGGACTATGCATCGCTTGCCGCCAGGATTGCCGTCTCCAACCTGCACAAGAGCACCAAGAAGTCATTCTCCGAGACTATCAAGGAGATGTACCAGCACTACAACGAGAGATCTGGCCTCATGTCCCCGCTCATTGCAGAGGACATCTATGAAGTCATCATGAAGAACGCCTCTCGCCTGGACAGCGAGATTATTTACGACAGAGACTTTGATTATGACTACTTCGGCTTCAAGACTTTGGAGAGGTCTTACCTCTTGAAGCTCGGTGGCAATGTCGTCGAACGGCCACAGCACATGCTAATGAGAGTTTCGGTAGGGATACACAAGGACGACATCGACTCTGCAATCAAAACCTACCATCTCATGTCCCAGCGCTGGTTTACTCACGCTTCGCCAACCCTTTTTAATGCTGGCACCCCCAGGCCACAGTTAAGCAGCTGTTTCCT

1. Gene ID: DN90778_c0_g1

GAGGAGAAGGCGGTGGTGTATATTGACAAGGAGAGGCTGCGGGTGCTGCAGGAGAGGCGCTGCGACAGCAAGGGGAGGGGTGCTGTGTACGATCCTGTTCTCGGGATCTGCTGCCATTTCTGCAGGCAGAAGAAACTTTGCGGCGAGGAGGGATGCAAGCGCTGCGGGGAAGGAGACTTTGACCAGCCATGCATAGGGAAGACAGAGTGCTCCTCCTGCCATTCCACCAACGGGATACTCTGCCGTGCTTGCCTTAAAGTTAGGTACGGCGAAGAGATGGAAGAGGTTCGGAAGAACAAGAACTGGATGTGCCCTCACTGTGTTGAGGAGAAGGGCACCAAGAAGTTCTGGATATGCAACAGCTCATTCTGCTTGAAGAAGAGGAAGATAGCACCAACTGGGATTGCCATATTCCAAGCGCGTGAGCAAGGATATGCGTCAGTTGCTCACCTTCTCATGGACCAGCTGAAGTGCCGTGGATCATTCTGAGGACTACTACTGCTTCCCACATGCTCACTTCTTCAGTGATTCTAGTGATGATTGGTGGTTGCTCTGGATCTTTTGCTCTGTTAGTTGTGTTTAGATGGTTGACAACGAGCCATCTTAGGAGAAAAATAAGGCACCACTCCTCTTCCTGAAGAATGTGGAGCTACCAAAACCTTCATCCCAGATGTTCTCAGACAGTGAGGGTGACCTTCCCTGAAGATCGAAGCAATGATTCTGCGTTGCCTATGCACTGTTAAATGTAGTGGATGAGCTTCTGAGCTTAACTATGTGAACTGTTAGATGTAGTGAGGTGTGTGTCTGCTGCTTGCGTTCATTACCTGTAAAACGTCTGTTATGTGCACCT

1. Gene ID: DN74659_c2_g1

AATGGGTTTTATTATTGGCATATGGAAGCGAACTTCCACATCGCGAGATATTGTGTTGTATTCGGCTGCTGCACAACCCTGCACCCTCACGATCGAACCCTTCGTGCACACATTATTATTCATGGCAAGCATGCACCGGCAGCACTCCCACATTCCAGTGCACGCATACTGGTAGGAAGACCTCCTCCTCATTCCCCTCAGATGGAGATGCTGTTGGGGATGCCCCTGGCTGTGAGCCCTTCGGCGTCGCCCTTGTAGTCAGAGGTGTTAGGGTAGAGCAGCATGTACGGAAACTTGGCCGGCCCGTTGCGGTTCTTGAGCTGCGGGTTCGCATTCATGGCCACCACCTGGCGCTCGATGTCCTCTAGCCTCGCGCCGAACCTCTTGAATGCCTCCAGCGCCTTGGCGTCCGACGTCCACTCCGGCGTGTCATGCTGCCCAAGGTAAACCTCGTCGGAGGAGTGCTTGGACAGGATCTCCAGCAGCGAGATGCCGACGAGCGCCTGGAATTGGTTGGTGATGGTTCGGAGGAACACCTTCTCCGGATCGCGCGTAAGCTCAACATACTGCTCGGTGTCCTTCTCCGGCATCGGGCGCCGGCTCGCCGACGGCTTGTTTGGGTGGTAGCCCGCGTACGGGTACTGCCCGAAGTTCACTGCAGCGTGCAGCGCGGACCCGGTCCAGATGATGGTGGCGCAGGCCTTGACCAGCTCCGGCACCGTCTGCATCTTGGGCCACCACGCCGCATCCTTCAGGTCGCCGTGCCCGACCTCGCGCACCTCCTTCCACCACGCCTGAAGCTCCACATCGCCCTGGAGCACGTGGTCGTTGGGGTAGTAGATGGCCACGTACTCCGCAACCCACTGCTCAATCGCGTGCCACACGGCCAGCCCGTCCGCCGCGTATGGGTAGTCCTCGATCAACAGCCGCAACTTGTATGGGCTGGATGGGTCCTCCACTGCCATGCTCCTCTTCATTAGATCATCGGGGAGAGCCTGCTCGACGAAGTTCCAGTTCTTGTAGACGACGGAGGACAGCTCCATGGCGAACTTGCGCGGGAACACCGTCATCTCGATGATCCCGCTGGTGTTGATGAGCGCCTCCCGTGCCCGTGAGTTGATGTTCATCGTGTCGCGGTAGTGCGGCACCAGGAGCTTGCTCACCGGGTGGGTCACGCTGAGCTGCCGGTTGGTCGAGATTACGAAGGGCTCCATCACGGCGTGCGTGTTGAGCCAGTGGCTGATCAGCTGGTGCCAGCCGTAGTCGTTCACGGCGACGTAGGCCTTGGCGAGCTGCCATATCCAGGCCTCGACGCCGGTCGACTCCGGCGTGTACACGGTGCTCTTCGCGGTGGTCAGGCTCCCCTGAATCAGGGGCGAACTCAGCTCGATAGCGACCGGTGCCAGCGTTCCGTCGCCTCGCAGGAACAGAAGTGTCCTGGTCGCGTAGATGAAGCTGTCGTCCAGGTTGTTGATGTTAACCAGGAACGGCATCAGGTGGTCGTGGTGGTCCACGATGTAGAGTCTGTTGCTGGCGAGTGCCTGCTCCACGGTGAGGCCCTCGAGGTTCTTCCTGATGTGAGACTCGGTGACGGTGCTGGTGTGGTCGCCGTACTTGCTGGGATCGAGAGTACTCTTGGGAGGGAACTCTGTGAGACGCGTGATCATCATTGGGTTCACGCCGGCAAGAATCTCCCTAGCAAATTCTTCGTCAGTCATCCATGCTTTCTCATCCTCTTTGATGATTTGTGGCTTGGGGAGCTTGAGGATGTAGTCGCCGCCCATAGGAACGAGGTCCTTGAGGAGCTCGAGTGGGAAGCGCTTGCGCATCTCCTCCAGGGCCGGGATGTTGGGCAGCTTGATGCCGCCCTCGTAGAGCTTGATGACGTCCTCAAAGGAGTCGAATTCGCCGGGGGAGAGGTCGACGTACGTGCGGATGGCCGGCACGATGCCATCAACGAGCGCCTTTATCGTGTATCCGAAGAAATCTGCCATCTTCAGGTGCCCGAACACCTCGTCCCGCGGCACGTAGATGGTGTCCACCACCGGCAGCAGCCGGGTTTCGTACCTGGGATCGGTTTGTGAGAGCTTGCGGCCGGTGCGGCCCCGGCGAGGGTAGGGGTGGTCGTCGGTGCCACCGAGGATGGGCCGGCCTTCGCCTAGGTCGTTGTAGACGTCGTAGCGGTAGACCCGGTCGTGCGTCTCGTAGGGGCCCTGCTGGTCGTCGCCCCTCAGGTTTCGGAGCTCGTCGTCGCGGTACGGCTTCAGCGCTGCCGGCATCTGGTGCGGCAGGTACGTATTGTTGGCGAAGAAGACGCGATTGTAGCGGTATTTGGCCTGCGGGTAGACCCACGAGTTGGCGACGAAGACGACGGTGCCGCTGGCGCTGCCGGGCACGTTGTTCAGGGTGATGCTCTTGAGGAAGAACTCCGAGGCGTGGTTGTTCTTGACGATGATGGCGCCGGGCACGCCCAGCTTGTCCACCGACCAGTCGAAGGTGACGCCGAACTTTGATTCGCCTGTGGTGATGGATGGTAGGCTCGTCGGGATGAGCCATTGCTCCAGGCTCGCCTCTGCTCCGACCTTTCCGCGGTTCCCGTTGTTGTGGTCTACGACGGTGGAGCTGATCAGCTGGCAGGTGACGCCGCGGCCGAGGAACTCGGCGATGCCGTCGACGACGGTGGCGCCGAAGTCGTTGAAGTCGAGCACGTTCTTGCGCATCAGGACCACCGAGCCCTTGAGGTGGGCATTCTTGAGACCACCCGTCAGGTCGCCGATGATACCGCCCAGCATCTTCGTATATGCCTCGGAGCGCGGAACGCTAGCTAGTAGTCGTAGTGTGTGAGGCTAGCT

1. Gene ID: DN77497_c2_g1

CTATGGGATATCCGGATATGGCCTATGGCCGAGTGTGTTCGCGAGTGACATGGAGTACACATATTCTGTCGGTGGTGTGTTGTGGAAAACAATAAGCAAAGCAAACCAACCAAGTCTCTTATTAGTGCACGAACTTAAACCGGAAAACACACACACTCACGCTTGATCTGAAACAAACATACTACACCGAGCCTGGGACATGTGCACCACACTAACACACGACTGGCACAGTGGCGCGCACACACACACACACTACTACGTAACTGACATCAAGCGGAGGCGGAGAAGCCCTCGTCACCAGCGACGTCGACGGCGGTCTCGATGGCGCTCCTGGCGTTGCGGCTGAAGCAGCTGCGGCGGATCTCGCCGACGTTCCCGCCGGGCCTGGGCGCGGTTGCGAGCTTGGCCATGGACTTGGCGAACTGGGTGAAGAAGGCGTCCTTGCTATTCGCGAACTGCCTCACAATCGGCGCCGTGCTGGCGTCCCTGATGAGCACCATGTCGGAGGTGAAGACGCCCTGGCTGGCGACCAGCGCCCTGTAGTAGCCGTTGTCGAACAGGTCCGGGGTGACCACGTCCAGGTTCTGCCGCAGGTTCGGGTTTCTGGTGCAGTTGGCGGCCAGCCTATTCCCGAACGCGTCGCCATTGGCGGCACGGTCGGAGAAGAACCCGCAGGCGGCCCTCCCGACGGTGTGCGCGCCAGAGAGCGCCACGAGGTCGGCGAGGTCGCGGAGGCCCTTGTCACTGAAGGCCTTCGCGAGGGCGGACACGCTGGAGGTCGAGGGCGCGGGGAGGCCATTGACGGCGTTCTGGGGCGCCGGGGTGAGGCTGTCGAGGACGCCCCGTGGCACAGCGTAGGTTGGCCCGCCGGAGAAGACGACGGCCGCGCGGGTAGCGAGGGTGGAGATGTCGGCGCAGGAGACGGTGGGTCCACATGCCGCGTGCACCTTGGCCCGGATGTCCTCGATGAGCTGCAGCGCGCGCGGCTGCAGCGTCCTGTTGGGGCCCATGCTCTGCTCGGTGCCGCTACCCTTGAGGTACACGGAGGCGTCGCAGCCGTTGGGTAAGCAGTCGTGGAAGAAGATGCGGAGGAGCCCCGCGGCGAGGGCGATCTCCTGCCGGAGGGCGGTCTGCACTGCGGAGGTCACGATGCTGTCCAGCTGCGGGCACGTCTGGGACAGCGTCGGGGAGAGGAGCACCGACACAGTTAGGAAGGCTAGCACCCGTGCGCCGGCGGCTCTGCTGCTGCGGCTCGCCATCGCCATTGGGAGTGTGGTGTTGTGACTTGTGAGCTTGC

1. Gene ID: DN86845_c0_g2

CGAGCATATGAACCAGCGACAGTCCTACCAGCTTGGTTACAAGGGCATAGCCCCTGCCTGCAGTTAATCCGCGAATACCCAATATGCAAATTTGTTGCTACATGCATACCACAGAACGATTGATTATACCACCAGGATAAGGGACCAGGCAACGTTCAAACGACAACGCCAAGTTTCAGCACAAGGCTGTGCACACTCCTACACCTCCCTTAAACTAGCAGCACAAGCAGGGAACAGTTGCAATGTTTCGCAAAAACTATCTTAAGTCTCCGTCTGGCCAAAAAGTGTCACAAACTTCAGGTTCCAACGCTGCGACTGCGTTCATCTTCAGATAGTTGTTGGCTGATCAGCAACTGGGTGCCTTCCCTACAGCAAAAATTGTCAGCTACTTTGATCCCCTCTACAACGCTCCCAGGTCTCAGCTCACGCACCTTCAACGAGCTGAACCAGGAGTTTGAGGTCTCTAGACCGCTACACCTGTCACCTGGGTAGGATTTTTACACTGGAATTGGGACCTTGGGGCTAGATGGTCTCCAATTACTTTTTCTTCCTCTTCACTGAACATGAACCACTTGTCATTGCTTGCTGTTGTATCAAACTCTTGGCGCATAATTGCTGTCTTTTCATAATAGCCATCCAACTTCCAGTATGCCTCTCCTTTGTCTTTCCTGACAGGCTTCGCCCTGATTAGACCTCCCAGCTTCTCCTCATCTACTGCTGCCTCTTTGTCTTCGTTTAACTCCTTGATTTGAGCAATGAGACTATTTATTTCTGCTCCTTTTCTCAAGAACATTGATTTGGCCATCTCATTCTTTAGTTTTTCTTTCAGCTCCTTTTCCTTCTCCTTTGCAGCACGGATTTTTTCTTTAGCAGCAATCTTCCTCTCAGTGGCATTTTCATTTTGTTCGATGATCCAGTTTCTCAACTTACTAGTAGAAAGGGTCTCATCACACAACAAATTTAATACATGCAGCTTTAAAGAAGGGCTCAAACTCTTGTATTCTAGTTCCCCTTGATCCAAAGAGCCAAGAGGCAATTCCTTTGAGATGAGTGTTGATTCACTAATATAATTGCCAATTTCATCCATCCAAGCATCTCTGTCGCTTGAATAATCTGAAGGGTTCTCTCCTCTGTCTTCTTGGATGACAGACAGCAAACTAATGTGAAAGTCAGAAACAACTAAGGAACCCTCTCGATTACGACGACCTCGTCCAGTTATGACTTGTAGAACCTTTTCTGGTTGTCCCTCCCTTATTTGAAGCACCTCTCCAAATGTGTGGCAGAATTCAAGAAACTGAATTGCAGAGCCAACATCGTCATCCTCCAGCTCTGTCCCTAGAATGTTAGTGACTGGAGTACCTCTGGGTAGCTCAACTTTGGCCTTGAGAACATCAGTGCTCTCCTTGAGGATTTGAGGATCACCCTTAATGTCAACAAGGACCTTCTCATCACCAGTTAAGCTATTTGCCCTGCAGCTGCTCTTCTTCAGTTTCTTCTTGGCAGGAAGAGATGGAAGTGCATTGAGATCAGTCCCAATATTCTCATCCCCTTCAGCCAACAATTCATCAGTTGCATCATCTGTTGCGAGGGCCCTCTTTGTGTTGCCCTCCTTCTTGGCCTTGGCAGGGAGTGATGACAGCGCCTGTGCAGCAATCACAATGTCTTCGCCTTTGGCTAGCAAATCATGGACAGAGGTGTGGCCCGATGCCTTGGCAGCATGAGCCAGTATTCCTGTCGGCGTCTCCCCTTTCTTCTTTCTGCAGAAGCTGCAGTTGCAGATGCCCCTGCATTTGGGGCAGGTCCAGGCCTCCTGTTTGGTCATCTCCTCAGCGTTCTCACCATACCTGTTGAGCAGGCACTTGTGGCAGAAGTGGATTGGACAGAGCCCCTTCTTCCGCGGCTGCTTGCAAGCCACCGCAAAGTCGGTGGTTTTCTGACGGCACTGGTGGCAGGTCTTGCCGTTCTCCGGGTCATAGATCCGGCTGCCGATCAGACGGACTCCGGGGCACGGGTTGCGTTTCTTGGGAGCGGCTTCCTTGATCTCCTGCGTCTGCTCCGGCAGGACCACGATCTCCTCACCGGCGGCGGCGGCTCCCTTGTTCGCCATGGAGATGGCGGCGCCCTTTTTCCCCATGGAGATGGGCTGGAGGACGGGGAGAAGGGGCAGAGAGAAGTGAGGAAAGCTCAAGAGGCTTGGGTGCGGAAGCCGCAAGGATTTCCTCCGTTGTGGTGTTGGATGTGATAAATTTTGGGAACGGAAGCGTCAGGTCAGATCATAGGGCGGGGGATAGGTAGGGTGGCATGCCGCCCGTGAAT

1. Gene ID: DN60089_c2_g4

AAAGGAGCTCGCAGTCTCCTTCCACCGCTCGGTGTTTGATCTGATCTCTGCGAGCTGAAAGTTCTGGATCCCAGTAATCTACTTGAAACTCGACTCTGATTTGAAGGCTCGATCTGTGAGCTGGAACTGCAAGTTTGACGATGGAAGGAACCATTCCGATGTCCTCCAACACAAGGATCGCTGTGGTCACCGGCGGGAGCAAAGGGATCGGGCTAGAGGTGTGCAAGCAACTGGCGGCCAGCGGCATCACGGTCGTTCTGACAGCAAGGGACGAGACGAGGGGAACGGCGGCGGTGGAGCAGATCAAGCAGCTGGGGCACTCAGATGTCATATTTCATCAGCTGGACATCACGGATGCTTCCAGCGTCGCTCGGCTGTCCGATTTCCTGAAGAGCCATTTTGGGAAGCTTGACATC

1. Gene ID: DN81556_c0_g2

CCATGTGATGTGCATCCTCTGTAATTCTAGGAGATCTGGCATTGCGTTGCTCTAGGCTCTAGCCAGAGAAAGCCATTGTCCCGATCAATCGGAGATGGATCGTGTGGTAGCTGCCGTAGTTGCCCTGGTGGTGGTGGTGTGCTTGCGGCTGCCGGCGGTCGCCCACGGGCAGTTGCAGGTGGGGTTCTACAACACGAGCTGCCCCAACGCCGAGTCGCTGGTCCAGCAGGCCGTCGCGAGCGCCTTCGCCAACGACTCCGGCATCGCCGCCGGCCTCATCCGCCTCCATTTCCATGACTGCTTTGTCAGGGGCTGCGACGCCTCCGTTCTGCTGGTGTCCGCCAACGGCACGGCTGAGCGCGACGCGCAGCCGAACAAGCCCAGCCTCCGGGGCTTCGAGGTGATCGACGCCGCCAAGGCCGCCGTGGAAAAGAGCTGCGCGCGCACCGTGTCCTGCGCCGACATCGTGGCGTTCGCCGCTCGCGACAGCATCAACCTCACCGGCCAGGCCTCGTACCAGGTGCCCTCGGGCCGGCGCGACGGCAACTTCTCCCTCGACCAGGACGCCCGCGACAACCTGCCGCCGCCCACGTTCACCGCGCAGCAGCTCGTGGACCGCTTCGCCAACAAGACCCTCACCGCCGAGGAGATGGTCATCCTCTCCGGCGCCCACACCGTCGGCCGCTCCTTCTGCTCCTCCTTCCTCCCGCGCATCTGGAACAACACCACCGCCATCGTGGACACGGGGCTGAGCCCGGCGTACGCGACGCTGCTGCAGACGCTGTGCCCGTCGACCCCGAACGCGAACACGACGACGGCGATCGACCCGAGCACGCCCGCGGTGCTGGACAACAACTACTACAAGCTCCTGTCCCTCAACCTGGGCCTCTTCTTCTCCGACAACCAGCTGCGGACCAACGCCACGCTCAACACGTCCGTGAACAGCTTCTCGGCGAACGAGACGCTCTGGAAGGAGAAGTTCGTCGCCGCCATGGTGAAGATGGGGAACATCGAGGTGCTCACGGGCACCCAGGGCCAGATCCGGCTCAACTGCAGCGTCGTCAACAACGGGTCGACCTCGGTGGCGGCCCCGGGGATTGAGATGGCGCACCTCTACCCGGGATCCACCGCCTCCATCGATAATATCGCGACGAGCTGATGACATACTTAAGTTTTCATATCAGGACTACGTGTGCACCGCTGGAAGAACATTAGTACTAAGTTAGGCGAAGATTGCTACGTATGCGTTTCATGTTTGTATATGTCCGTGCGGTGCATGCATGAGGCCTCTGTGTGTGTGTGGTTCTTTTTGATGGTGGCGATCGAGGGTGTGCTTCTGTTGTTCGCAAACGTGAATAAAACGAAGGTTTTGTTTTTCTTCTTCCTCCAGCCTTTTCCTCGCAAGCGACGAAAGGTGACTGCTGACCTTTGCTTGAGTTGGTAGGGTCCAGAGGGCGGC

1. Gene ID: DN64173_c2_g3

GTCAGTCATTAGCTTATTATTCCATCACATGCGTCGGGGTAGTATAGCAACATCTCTCGCTCCCTAAGATCCCGGAAACCCATGGCCGATCACATGGCATCATGCCAGGAAAAACGATGACGCTCATAAGTAACGTTCCCCTAGATCTCGTAGACGGCATCCACGCTCCCCGAGGCCACCGCCTTCATATACAACGCCTACGCTGGTCGCCCACTCGCTCGCTGCCTGATTGAGCGCGTGAGGCTCGAAAAGGGGGATACATTAACAAGTCGTGGCATTGTCAGTTTAGCTAGCTCCAACCCTTCTTCTGGTCAGCTAGAGCCGCCATGGCAATGTGGGTGTCTCTGCTTCCTCCTCCCAGGCTCCTGCTTCCTTTGCTGGTGCTGCTGCTGCCGGTGGTTGCTGCCGTCCATGGTGGGTATGGCCATAGCAACCTCACAGTCGGGTTCTACAGGCACTCGTGCCCCGACGCCGAGGCCATTGTCCGCGAGGTCGTCGCCGAGGCCGTGGAGGACGACCGGACCGTCACCGCCCCGTTGCTCAGGCTGCACTTCCACGACTGCTTCGTCAGGGGTTGCGACGCATCGGTGCTGCTCAACTCGACCAAGAACAACACGGCGGAGAAGGACGCCAAACCGAACCACACTCTGGACGCGTTCGACGTCATCGACGCCATCAAGGAGAAGCTGGAGAAGAAGTGCCCCCACACCGTCTCCTGCGCCGACATCCTCGCCATAGCCGCCAGAGACGCCGTCTCGCTGGTACGAAATGCTCCGTTTCCTCCATCACGGAACTCTGGTAGATTCAGAACGCATGAAAGCTGTCCGAGCTGGTTTTCAATGGGTCGTTGTGGTGTTTGCTCGATAATGTAACTAGGCTACGAAGGTGGTGACCAAGGGAGAGTGGCACAAGGACGGAAACCTGTACGAGGTGGAGACCGGCCGGCGAGACGGCCGCGAGTCCAGCGCCAAGGAGGCGGCGGTGCACTTGCCTGATTCCTTTGATGGAATCCTCAAGCTCATCGCGAGGTTTAAGTCCAAGGGCCTCGGACTCAAGGATCTGGCCGTTCTATCAGGTGCCCACGCGCTGGGGAACTCGCACTGCCCATCCATGGAGAAGAGGCTGCGCAACTTCACGGCTCAGTACGACATGGATCCGACACTGGACACGGCCTACGCGGCGACGCTGAAGCAGCAGTGCACGAAGGCCGAGGACAACGAGACGGAGCTGCCGATGGTGCCAGGGAGCTCGACGACGTTCGACGCCACCTACTACCGCCTCGTCACGGAGAAGAAGGGCCTCTTCCACTCGGACGAGGCGCTGCTCAGCAACGGTGCCACCAGAATGCTCGTGTACAGCTACATGAGGTCGGAGAAGAGGTTCTTCAGCGACTTCGGGGTGTCCATGCTGAACATGGGCAGGGTTGATGTGCTCACCGGCCGCGACGGGGAGATCAGGAGGAGATGCGCCGTTGTCAACTAATATAAGCTAGCTAGCCGGCTGATCTACTGTGTGGTGTTGGATCTTGCCAATTTGATGTACTAGTGTGGAACTGCTTTTGCATGTTCTATAGATGTGTAGGAACCGATGCTTTGACTTTGGGATTCCGAGCTTTGGGTTGACAATTGTAATTGTAGTAATGTTTATGGAATGTGATAACATGTTCCATAACTTTTGGGTCAGCTGTCATGCAAGCAGCAAGCTTTTCTAGTAAAATGGCATATGTTGACCCACAGAATGAAGAAAAAACATGC

1. Gene ID: DN83151_c1_g1

GCTGCATACCATAACCATATGAGCGATGAAGTTGATGCTCTGTTTGGTTTAGACAAGGTGATCTCGAAAACATCAATAGTCCTGGGGAGAGATGAGTACATGAGGAAGACCATTGCAGACATATTGAAACATTACAAGCAAATCGCAGTTCCGCGATGAAACAAAATTCAGAAAAGCGTGCCGTCATCTACTTGCTCGTCAGCCGTGCGCAAAATTCCACAACGCAAGAACGGAATCTTGCCTTCCCCAGCTTTACGAACGTTGTACAAAATGTAGAAACCAGCCCCCAAAAAAGGTGACCAGCTCATAAAGAAAACTCTGTTTCAGCCCGTTTCAAGACGCGTGAAACCATGGCGTAATACTCAGGTTCTTCCTCTTTCACTTGATCGAGCGTGGTGGATTCGTGCGCATCAGCCTCAGTGAACTTGAGAAGTATCAGGTTTGCCACGCCGGCAGGCTTCGATTTGTCCCACCGAACAAGCATTAGTTCATTGACAGATCTCCACATTTTTCGGCCAGTGTCCTTCATGTTTTGATCTCTAGCACTTCGGCCACGCCACAATTCTTTCACAATGTACATCACCTCTTGCGCATCAACCAAAACTTGTTCTGCTGACCCATATATCAGCTCCTCATGCTCAAGAAGACGATGATGAAGAACACGGTAATGATCCAAATCCAGGTTAACAATTGGTTCAGTTTGAAAATCTAAATTGGCTAGCTGAGTAACCACATAGGAGGCCATAACTTGACCAAATATTGCAGGGATTGTTCCCAGTACTGGGATAATGCGAACCCTGAATCCTGGAACAATCTGGTAATCTGATGGAGTTTCCTCTTCTTTCGAACCTTGAAAAGGAAGTAACTTTGCCTTGGGCTTCTCCAACGAAAAAACTACTGGTATTCCACCTTCAATTCCATGATCCTTCTTCAACCGATACCGTACCGTCCTAGAAAGGGGGTCGTTACTTGATTCTCTCAAATCTGCAACACGAATTCGGGTTGGGTCAGCTCGAGCTCCAGCCCCCATTGCAGAAAGTACCCTTAACCCTCTGCGTACGCAAGCCGCAAGGAGAGCCACCTTAGTATCAATGTTATCTATGCAATCAAGAACAAAATCTGGCTGTCCAGAAAGAATTTCGTCCTCGGCTGATGGATCATACAACTGCACTCTTGCTTCTATTTTGCACTCTGGATATATCATTGAGAAATGCTTCTTGAGGCATGACGCCTTTGGGGTCCCAACATCATCTCTGGTTGCCACAGCATGCCGGTTTAGTGATGAGAGTGAGACCTGATCAAAATCTACTAGAAGCAACCTGCCAACCCCAGATCTGAGGAGCATGGAAGCAGCATGACTGCCAACTCCTCCAAGACCAATGACTAC

1. Gene ID: DN98969_c1_g1

GGAGGGGCCCTGGAGGCCGTTGAGGCCGAGGAGGGTGGCGTCGAAGAACATGCGGTTGTGATTGCAGTACCGGTCCCCGGGGTCCACGTCGTCGAAGCGCTGCGGGAACTGGACGAAGGCGGTGTCGTCGCCGTCGCGCGGGTCCAGCATAAAGCACATGGCGGCGCGAAAGGCTTGCGAGTTGTTGATGTAGTGGTCGCCGTCGAAGTTGATGATGAAGGGCGCGTTGGACAGCAGCGCAGACACGCGCAGCTGCACGTTCATGGCGCCAGCCTTCTTCTGGTGGTCGTACCCCGGCCGTTTCTCTCGGGCAATGTACACAAGCGTCGGGAGGCGCACGTCGACGGCGCTGAAATCGAGAGGGTGGTCGGAGCTTGCTGGCTTGCCCAACTGAGGATCAGTGCTGGGCTGATTCAACATGACCTGAAGAATTGCAGGGTGATGTCCCTTCTTGTGGCCCTTGGCTGGCTCAATCCATGTTCCTTGCCATTGCGTTCCGTCTGTCATCCAAGTTGCATTTACATGTCCCTGTTTCGTGTTTGCACTGTTGCGCGCATTGGATCGTTCCTCAATGACAGTAGAAAGCCCATCCAAGCATGCCTTGAACTCGTCATACTCCGCACGAATTCGCCTATGGTCATCAAGGAAGGCACCGGCTGCGTTCCCGGTGTACGGTTGCACATTCCTCCCGAAGTAGCTCTCGGGGGCCCTTGGCTCAACACAATGTTTCCGGCAAAATGGAACCCACAAAGCAGCAAACTTTGCGGTCTCGAGCAACCCCTCATAGTGAACAAGCGATCCACCATCATCCGAGAAGTATGTGGCATACTTCTCAGCCGGGTAGTCCGTAGCAAGGATCGACAAGATGGAGTTCATGGTGTAGATCATAGGCTCGTCCACCGGGTCGACGGTATTAATGAACACATCCAAGATGGGGAGGTCAGAGATCCCGTCGAACAAATCGAATTGTTCTCTTAGCTTAGATAGCTTGGGAGCACATTTTCGAGGTTTGAGTTTTGTGACTTGGTTTAGTAACCAAGTTACCCCAAACCAAAGATCCCCTACCACCGAGATCCACCATAACCACATTGAGTCTGAGTCTCGGTGCTCCATGCGCCAAATGAAAAATAGAATAACCACCATAACTCTAATCAGGGTCAACAACCTGTAAAGGTTGATGAGGAAGCCCTTGACCCTGAACGTCCGGTAGAGCAGCGGAGGCGTCCTGGCGGCGCAGTCTTGCCTCGCCAGAATCTCTTTCTCGTCGGAGGGCACCCAGTACCTGTCCTTGGCGCTGAGCACGATGTCACCGTCGTCGATCGACGCCGCCAGCAGCGGGTCGGCTAGACGACCCGCGATGTCACCATGACCGGCGCCGGCCGCCGGTAAAGACATACCTCGATTCTGGCCTACCACTACCGAGTAGCAGATCTAGCAAAGCTAGCTTCTGCTTACTGGAGGGAGGGAGATCTAAGGGAGCTACCACTGAACTTACAATGCCT

1. Gene ID: DN95036_c2_g1

CCAGGGACTATTCAGATAAAAATTGTTCCATGCAAATATTATACATTAATTAGATATAGGTGATATACAAGCACGATGCCCAACTGCCTATGCATGAGCGCACAAAGGTTGCTTTAATCCAGAGTTCATCATGCCGAGGCAGCTTACAGCTATCGAGATCATCAGACCGAACAACAAGCCAGCGGAAAGTAATTGTATGAGTCAGCGTAGCGCGTCACAAGGGCGAACGGCAAATCTAACTTCCACATGCCAGGCAGTCCTCTCGATTGTTCAAGGAGCAGGTCACCTGTGCCATTTTCGCCTCCAGATCCTCTTCCTCCTCGGCAAGCTTCTTATCCTTGAGAAGACTAGTGTCCACTGTGAATTTGATCGCATCAGCAGCAGCTCGCGTCCTCAGGTAATACATCCCAGTTTTCAGGCCCTTTGACCAAGCGTGGAAGTGCAGGGAAGTCAGCTTTCCAGAGCTTGCTTGATCCATGTGGATATTGAGGCTCTGGCTTTGATCAATATAGCAGCCACGGTCGACAGCCATGTCAACAACAGTTTTCTGCTTGATCTCCCAAACAGTCTTGTAAATTGCTTTTAGATCGTCTGGGATCTCAGTGATCTTTTGGACAGAGCCATCCTGGTAGATAATATTGTTCTTCAGAACAGGAGACCACACACCCATCTCGGTCAAATCATGGAGGAGATGCTTGTTCACTACAACAAACTCACCACTTAGAACTCTCCGACTGTAAATGTTTGACGTGTACGGTTCAAAGCACTCGTTGTTGCCAAGAATTTGGCTAGTAGAAGCAGTGGGCATTGGAGCAACCAGAAGAGAGTTCCTTAATCCAACTTTGGAAATCATCCCCCTTACAGCTGGCCAGTCCCATCTGTCCGATGGCACTACATTCCACATATCAGGTTGAAGAATGCCCTTGCTGGCAGGGCTCCCAACATAAGTTTCATAAGGACCTTCATTTGCGGCAATCTCAGCAGAAGCTTTCAAAGAATGGTAATAGATAGTCTCAAAGATATCCTTGTTTAACTGCTGGGCCTCTGGTGAATCGAATGGCATGCCAAGTAAAATAAAAGTGTCTGCTAGGCCTTGAACACCTATTCCAATTGGCCTGTGCCTCATGTTTGATCTCCTTGCTGTCTCAACGGGGTAATAATTAATATCAATTATTTTGTTGAGATTCCATGTCACAAGTGAAGTAATCTCAGCTAGTTTGTCGAAGTCAAAGTATCTGTTTTTTGATCCACTGCTGCCAACAAGTTTAGCTGGATGTGACTCTATAGGAACACCATTTTCCCTTACAAAACGTGGTAGAGCAATTGATGCAAGATTGCAAACAGCAGTTTCGGTGGGACTTGTGTACTCAATTATCTCCGTACACAAGTTGGAAGACTTAATTGTGCCCAGATTCTGCTGATTACTTTTCCTATTGCAAGAGTCCTTATAAAGCATATATGGTGTTCCAGTTTCTATCTGTGCCTTCAAAATGTCAAACCAGAGAGACTGTGCTGACACAATCTTCTTTGCCTTACCTTCTCTTTCATATTTCTTGTACAGATTATTAAACTCATCTCCCCAGCAATCAGCCAAACCTGGAGCCTCACTGGGACAAAATAGCGACCACTGCTCATTACTTTGCACCCTTTCCATGAATAAATCAGGAATCCAGAGAGCATAGAAAAGATCCCTGGCACGATTCTCCTCCTTTCCATGGTTCTTCCTTAGATCAAGGAACTCAAAAATATCAGCATGCCAAGGCTCCAGGTATATAGCAAATGCACCTTTTCTCTTGCCTCCACCTTGGTCAACATAACGTGCAGTATCATTGAAAACACGTAGCATGGGGACAATTCCATTGGAAGTTCCATTTGTTCCTCGAATGTAACTGCCAGTAGCACGAACATTGTGAATTGAGACACCGATTCCTCCAGCAGATTTGCTTATGGAAGCACATTCGGAGAGAGTATCATAGATTCCCTCAATACTATCATCTTTCATGCAGATAAGAAAGCAGCTACTTAATTGAGGCCTTGGAGTGCCAGCATTGAAAAGGGTTGGGGAAGCATGAGTAAACCAGCGCTGAGACATCATGTGGTACGTTTTGACAGCAGAGTCGATGTCCTCCTTGTGTATCCCAACAGAAACTCTCATTAACATATGCTGTGGTCTTTCCACAACCTTTCCACCAACTTTCAACAGGTAAGACCTCTCAAGCGTCTTGAAACCAAAGTAATCGTAGTCAAAGTCCCTATCATAAATTATCTCACTGTCCAAGCGAGTAGCGTTCTTCATGATGATCTCGTAGACGTCATCAGCGATGAGGGGAGACATCAATCCAGACCTCTCGTTGACATGTGTGTACATGACCTTCACAGTCTCCGAGAAGGACTTCATGGTGTTCTTGTGCAGGTTGGAGACCGCGATCCTGGCCGCGAGCGAGGCGTAGTCGGGGTGGGAGGCAGTGAGTGCGGCGGCGGTCTCGGCGGCGAGCTCGTCGAGCTGGCTGGTGGTGACGCCCTTGTAGACGCCGGCGCAGACCTTCTGGGCGACGAGGACGGGGTCGCAGTGGTCCTGGCT

1. Gene ID: DN75230_c0_g1

ATCTCTTTGGGGTACGTTTTGGGGGACGCGGCCGGAGATGCTCTTAGGCTGCCGTAAATTTGATTCGTTGGCTTGGAACGGGACTGTGTGCGTGACTGCACATACACATTTCTTTTTCTTCAATTATAGGTACGTTGCTCAGTTCTCGAGGAGCCTTTACGTCGAGTACAGAATCAAAGGGATCGACGTGCAATGCCAGGTAATTTACTATCAGTCTCACACTCTCGGCCACACTCTGCACATGGAGTAGATCGAAGCACTGACATGCATTCAATCTCGTGCAGGCCCCGTTCTTCGTGGCGACCGGCATGGTGTCGAGGCTGTCGGAGACCAGTCGCCTCTCCCTGCTCCTCGTCGCGCCGACCCCCGACGCCTACGCGCGCGCGGCGGTGCGCTGGATGGGGCACGGCCCGCCGCTCTGCGCACCAAACCTCTGCCACCAGCTCCTGTGGTGCCTCGCCGCCGCCGTGCCGGACTCCGTCCACGACTGGCTCCGCCTGCGCGCGAACCTGCATCACCGAGAGCTCTTCCACACAAGGTCGTCAACACGTCGTGAGGGCGTGCAAACAGTCACGCGAACTCCCTAAGAAGAACCTGCATTTATCATCTTTACTCTTTTGGAATGTGGACCGGTTGAATGCGCCAGCCCTGCGCGATGTGATCTTTCAGCTTGTCACTCCATGTCGGGAGCAAGCGATGTAGTGTTTGAAGA

1. Gene ID: DN87390_c0_g3

GTGCGCGCGCCGCTGATCGTGCCGCTGCTGCAGCTGTCGGTGGCGGTGTGCCTGGGCATGGCGGTGCTGCTGTTCCTGGAGCGGCTGTACATGGGGGTGGTCATCGTGGGCGTGAAGCTCCTCCGCCGCACCCCGGAGCGGCGGTACAAGTGCGACCCCATCGCGGAGGAGGACGACCCGGAGCTGGGCAGCGCCGCCTTCCCCGTGGTGCTCGTCCAGATCCCCATGTTCAACGAGCGCGAGGTGTATCATCTGTCGATCGGCGCCGTGTGCGGGCTAACGTGGCCGTCGGATCGGCTGGTGGTGCAGGTGCTGGACGACTCCACGGACCCAATTATCAAGGAGATGGTTCGGTTGGAGTGCGAGCGGTGGGCGCACAAGGGGATCAACATCACGTACCAGACCCGGGAGGACCGCAAGGGGTACAAGGCCGGCGCGCTGAAGCAGGGCATGAAGCACGGGTACGTGCGGGAGTGCGAGTACGTGGTCATCTTCGACGCCGATTTCCAGCCCAACCCCGACTACCTTCACCGCACCATCCCCTACCTCCACCACAACCCGCAGATCGCCCTCGTCCAAGCAAGATGGAGGTTTGTGAACGCAGATGAGTGCTTGATGACAAGGATGCAGGAGATGTCCTTGGATTACCACTTCAAAGTGGAGCAAGAAGTGAGCTCGTCTGTCTGCGCGTTCTTTGGCTTCAACGGGACCGCCGGGGTGTGGCGCATTGCTGCATTGAATGAAGCAGGAGGCTGGAACGACCGGACCACCGTGGAGGACATGGATCTGGCTATCCGGGCCAGCCTCAAAGGGTGGAAGTTTGTCTACCTCGGCGATGTCCAGGTTAAGAGTGAACTCCCCAGCACTTTCAAAGCCTTCCGGTTTCAGCAGCACAGGTGGTCGTGTGGTCCGGCAAACCTGTTCAGGAAGATGCTAATGGAGATTGTGACAAATAAGAAAGTGACGATCTGGAAAAAATTTCATGTCATCTACAACTTCTTCTTGGTGCGCAAGATTGTCGCGCATATCGTGACTTTCACCTTCTACTGCATCATCATCCCGACAACCATCTTTGTCCCTGAGGTTCATATACCAAAATGGGGCTGCGTCTACATTCCCTCAATCATCACACTTCTCAATTCTGTTGGAACTCCCAGGTAATTATAGCCAGTAAAGAACCTAACGATCAAACAAATATGTACAGAAAGTAGTGCACGTTTGGTTGTTTGTAATATATGATCAAACTCTGTGTTTTGTTGACCAAAATGTATCAATTTGGCAGGTCTTTCCACTTGCTTTTCTTCTGGATCCTCTTCGAAAATGTCATGTCGCTGCATAGAACCAAGGCCACATTGATCGGCTTGTTAGAGGCAGGCAGGGTGAACGAATGGGTTGTCACAGAGAAGCTCGGCAACGTTCTGAAGATGAAATCGGCTAACAAAGCATCAACCAAGAAATCATTCATGAGGATGTGGGAGAGGTTAAATATTCCTGAGCTTGGCGTGGGAGCCTTCCTCTTCTCCTGTGGGTGGTATGATGTCGCGTTTGGGAAGGACAATTTCTTCATATACCTTTTCTTCCAATCGATGGCTTTCTTCGTCGTCGGTGTTGGCTATGTTGGCACAATCGTCCCTCAGTCATAACCGAAAACATGTGGAGAATATAGCGACCGCCCTTGATACGCAATTCCTCGAGGAGAATTCAGCAAAGCTAGCAGTGCATAGCCAGTTTGAAGTAAAGATCAAGCAGTCTTCTCTCAATTTCTCTCTGCTTTTCTTTCTCTTTGGAGACTGATAGTATTGCGAGCAGCATTTTCTTTGTATGTGCTATTGTAATAGAAAGTGCAGTGCAGTGTAAGGTCACTGCAGGAGTACTGTGGTGTTCCATTTTTCTTCTTCTTGTAGCAGTACGTGCCGTGATTAAGGGGAGCAGGATGACTGCTTCCAAGTCAAATGTAATACTAATAGTATAAGAAAAGGAAGAGGTTCAGACATGTGTGAGCAAAATCAAACTGTACATGCTACTCTTTAGGCCACTCCATTTCACGTGTGCTAAATGAACAAAGATCCCTC

1. Gene ID: DN64596_c1_g1

GTACACATAATTTTCTTTTAATATTCTGAAGAGCTATCAATTCTACAAGATACAAAGTTTCTACTTGCTACAAGTACGTACATAGGTATACGGCCGTCGTCTGCACTAAAAATGACTAAGTACGGCTGTATGAGTTAGCATAACCTAAACTAATCACAATTTTTTGACTGCTCTTTTAAAAAATTAAGCAATATTTCATTTGCCACGTCACTACTGCTAGTAAGCTAGCTCTCTAGCTAGGCTTGTCGCTGCAGCTCCGTGGCGCTGATGAACGTGCCGTGGAATCCATACGGCACGCGCCCTGGCAGCTTTACGGCGGCCTCCAGGCGCATGTCGCTGGCGTTCACCACCAGCAGCTCCGACGACGTGCCGCCTCGGCTCTCGTCGTGCACGAAGCAGAGCACGTGCCCGTCGTCCTCCGCGCCGGAGCCGTCCGCGCGCGGCACGAAGCAGGGCTCGCCGCCATACCGGCCCTCGCCGTAGGTGAACTTCTCCACCGTGCCGGCCTCGAGGTCCACCTTGGCGAATCCTGACACCTTGGGCCAGGGCTCGGCCATGGCGAGGTAGGCGTAGCGAGTCTTCCTCCCCAGGAGCTGTCGGTTCACCATGCCGGCCTCGAGGTTCACCTGGTCGGCCTCCCGCAGCACGGCACGGCGGGTTGAAGTGCCGGTGCGCGGGTCGAGACGTATCTCCGAGAGCACGCTCTGGAAGCTCTCCTCCTGGTCCGGCGACTCGTTGAACACGGCGTCCGCTGGCGTCATGCAGGAGCCGATCACGACGATCTCGCCGGTGGACTCGTCCTCCCACGCGTTCCAGAGATGGAAGCAGAAGCAGTCCGGGACCTCCACCCACAGGAGCTCCGAAGCGTCGGCCGCGCGCTTGGGCAGCACGCCGAAGCGCGCCGTCTTGTTCTTGTCGTACACCACCGGCGATCCACCGAGCACCATCTCCTGGAGCTTGAACACCATCTGCTGGTCAGGCACGACGGCGTAGTTCTCGGTGATGGCGAAGTCGTGGATCATTGTCGGGGCGTCGACCGGGATCTCGACGTCAGGCGACTTGTGGCCGTCGGCGGTGAAGTAGAAGTACTTGAGGTAGGGCTTGGAGACGACATTGTAGCTGAGCGCGAAGAGCTCGCCGGTGGACGGGTCGAGCTTCGGGTGCGCGATCATGGCGCCTTCGAGCTGCCCGTGGAAGTCGTAGCGGCCGACCGTCTCGAGGTCGCCGTCGGGGGTGACGCGGACGTGGTAGGGGAGGTCGTCCTCGGACATGGCGAGGAGCCGGCCGTCATGGTACACCAGCCCGGCGTTGGCGACGCCGATCCCCTGCGACGCGTCCACCACGCCGCAGAGCGACCTCGCACCGAAAAGCGCCAGGCGCGCCAGGCCGGAGTGGCCGTGCAGCTCGCCGATGGCCTTGGGGAAGACGGGACGGCCGATGTCGCGCTCCTGCCTGAGCCGCGCCGTCTCCGTGAAGCGGCAGGCGTAGGACTCGGCGCGGCCGGCGCCCAGGCGCACGGCGTGGAGCATGCCGTCGCCGTCGAACAGGTGGTGCCCGCCGCGGGGCGCGTGGAGCGGGTTAGCGCCG

1. Gene ID: DN79085_c0_g1

AGTTTCTACCACGTATCTTGGTTTACAAGGATACCATCAATAATAGGTATCATATACTTGTACAAATGGAGAATGCTAACTCATTACACTGCCATCTTCATTTCTATTTTCTGGTGAGGATCATAGGAAACCAGTTTAAAATCTGAAGCAACAAATGAATCCATATCCTTCTTCAGAGGATTTATCTTCAAAATAGGAAAAGGCTTAGGCTGCTTCTTAATTTGTTCCTCCAAAGCTCGAACATGTGTGCTGTAGACATGAGCATCACCTATAACATGGACAAAATCTCCAGGACAAAGATCACAAACTTGCGCAATCATACATGTCAGAAGAGCATAAGATGCAATGTTGAATGGCACGCCAAGTCCCATGTCAGCTGAGCGTTGATACATCTGACATGATAACTCTCCATTCTCAACATAAAACTGTGCAAACATGTGGCAAGGTGGAAGTGCCATCTTCTTGAGATCTGTTGGATTCCATGCGGACAGAATGATTCGCCTATCATCAGGGTTATTCTTGATCTTGTTGATTACATCAAGTAACTGATCATAACCTTTCCCCGTGTAATCAGCATGCATGTTGGTATATTCAGCACCAAAGTGTCTCCACTGAAATCCATATACTGGTCCCAAGTCACCCTCCTCTCGTTGTGATAAACCAATACTGTCAAGATACTCCCTCGAAGCATTGCCATCCCATATGTGAATGTTTTTTTCCTGTAAAACCTTCGCATTTGTTGAACCACTGATGAACCACAACAGCTCTTCAAGAACACCACGCCAAAACACCCTCTTTGTTGTGAGTAATGGAAAATTCCTCCTCAAGTTAAACCGCATCTGGCAACCAAATTTTGATAATGTTCCTGTTCCTGTTCTGTCATTTTTCCGAGCACCACTTCGTATTACATCTTGAACAAGATTGAGATACTGATACTCATCATGCTTTTCGAAGATACTCTTAGGTAGAAATGAAAAGTTCTGAATTTCAAACTTTTCGTTCTTAGTATCATTGCCAGGTAATTCACTGACATTTGAATTGCTAACTTCTGCTACTGATTTTCTAACACGAACAAAGCTCACAAAAGAATGCCTAATATTGTTCTCCAGAACTGGAGCAGATGAAAACCACAGGTGGAAGACTGATAGATCAACAGGAGGAATGAAAGTGTCACACTCAATGGTCGACTCTATGTCAGTAAGATGGATGGCCTCACATGCAGATGCATTTAGAGATTCCCTCAGCACCTGTCCACCCCCTATTACAAAAGCTTTCTCAATGATTAAGCTGTAGGGAGTTGATGCTAATAGTTTTAAGGAAGCATCCAAGCTTCCACAGACAACAACATTCTCGACTGTTGCATAATCAGAGCTGCCAGAAGAAGTCAGTATAACATTCAAGCGACCCGGCAATGGTCTAAACTTAGGGGGTATGCTTTCCCACGTTTTCCTTCCCATTATAACAGCATTTTTCTTTGAGGGGTCTGATGTAGCCATTGTAAGCTCCTTAAAGTATTTAAGGTCACCAGGCAGCTTCCATGGCAATGCCCCATCCTTCCCAATGCCCATCTCACGGGTAGCAGCAACCACGACTTGATAGTTTCTCCGAATATCAGTGCGTGAATTGCCATTTGTGTCACTGGCCATCATCTGAGATTGGTGAAAGCAGGAAGACATCAACTGGTTGCTGGCTTGAAAAGGTTTAAGCCTTGGCGAACGAAATGTTTTGGTGGTACGCGTGCTGAAAAATTGCGTGACCGCCACCGTGCCTGCGGCGGCCGGCTGCTTGCTTCGCCGTCGGCGCCGCCGCCGTCGATGAGGAGGAGACGTCAAAAGGAATTTGGCGGGCAGTGTTTGTG

1. Gene ID: DN83175_c0_g2

TCGTCAGGACGCTCGCGCTCGCCGACCCCTCCCCTGAGTCGGACGCGCCCGCGCAGGGGTTCAGTTCAGAGGACGGCGTCGCCAACGTGCGCGCCCGGGTGCTCGGCGGCGGCACCGCCATCAATGCCGGCTTCTACTCCCGCGCGCACCCGGGATGGTTTTACGGACACGGAGAGGGTGTTGATGTGCCGGACTGGGATATGCATTTGGTGAATGCATCCTACGAGTGGGTGGAGCAAGAGCTGACATTCCAGCCGGAGGTGCATGGGTGGCAGGCGGCGGTGAGGGCCGCGCTGCTGGAAGCTAACGTGACGCCGTGGAACGGATTCACCGTGGACCATGTCACTGGAACTAAGATTGGTGCCACCACCTTCGATGCATCGGGCCGACGCCACAGTGCGGCGGACCTCCTTGCTTTCGCCCGTCCTGGCCGTCTCCGTGTTGCTGTCCGTGCTACGGTTACACGTGTCATCATTAACCCTATTGATCCAGCCGATCGCCGTGGAAGGTCACGACCAGCAGTAGGAGCAGTTGGCGTTGTGTACCAAGACCGTCTTCTACAGCAGCACCATGCCCTGTTGCGTCCAGGTGGGGAGGTTATACTTTCTGCAGGTGCCCTAGGAAGTCCCCAGTTGCTGCTTCTGAGTGGCATTGGCCCTGCTAGCGATCTTGCATCCCTTGGCATCTCTGTTTCTGCTGATGCCCCTGATGTTGGGAAGCATATGTTTGACAACCCTCGTAACGGCATCTCCATCATTCCATCAGTCCCTATTGATCACTCGCTTATCCAGGTAGTTGGCATCCCTTCTGCTAATGGGACTGCCTCCTACCTTGAGGCCGCGTCATACATCGTCCCCCTTGCTCCCATGCTGCGCCCTGGTCCTTTTATGAGCCCGTCTTCTCCACTCTATGTTACTATGGCAACTATCATGGAAAAGGTTCCTGGCCCATTGTCTGAGGGTTCGCTCTGGCTATCATCACCCAATCCGCTGGAGACCCCCTCTGTGCGGTTCAACTACTTCAGCCGTCCCGAGGACTTGGCGCAGTGTGTCGTGGGCGTGCGCCGTGTGGCACAAGTGCTCCGGAGCAGGACAATGGACATATTTCGTTCGCCATTAGGATCTTCGAGCCAAGGTAGGAGAGGGCCTATTAGGAGGGACTTCAGAATTGTTGGGGCAACACTGCCACTTGACTGGAGTACAAACGACACGTCCGTGGCAGATTTCTGCAAGCGAACCGTGACAACGCTGTGGCATTATCATGGAGGGTGTGTAGTTGGAAGGGTCGTTGACAAGGATTTCCGAGTTACTAGCGCGCGCTATCTTCGCGTGGTGGATGGATCAACATTCAGCGTGACACCTGGGACAAATCCTCAAGCCACAATCATGATGATGGGCAGGTATGTGGGGTTGAAGATGATTGGGGATCGGCACAGCAGAAGGCTAGTGAACAACACATCATCATAACCAACACACTCCCAATGCTTCTGAACATCCAATAACACCAAGTATTTCAGTGACATCGTACAAAGTGCGCCGTATTGGTCTTCCATACATCCAGTGCATAACATAC

1. Gene ID: DN101477_c1_g1

TAGTTCCCGGCGATCTGCACGGCCGGGTCTGCCGTGCGGGGAAGCGGGTGCGCGCTTTCGAGTAGGCCGGCGATAAGCCTGTCCTCCACCGCGTCCAGCGCCGCCGCCGCGAGCCGCTGGAACGGGTTGGCCGACTTGCCGGCGCGACGCGGCTGTGCCGGGACAGGCCGCATTGGCTTCTTCCAGGTGACGGCGGCAGATGCCGAGGTATCAGTGCTCTTGGTGGGGACATTCGGCTTGAGCCTGGAGGGCCTCTTGATCTTGGCCGGTGGATTAGCGACACCGGTGGGCGGGGCAGAGATAGAGGATGCCATTGATTCGGAGCTGATCAAGGCGTTCTTGCTCGTGGTAGACTCACAGACTCTTCCGTGTTGTTTGCCAAAAGCTGGGAACTGGGAGCTGAGGTGAGCGAGAGGAGGAGTGGTG

1. Gene ID: DN98385_c1_g1

GTCCCATACTCTTAGTTCATTAGCTGAACCAAACAGATACTAGTAGCGTATATGCGCGCCTCGACAGAACACGTACAGATCGAAGAACATAGCACATAGTACAGGATCTTCGTAATATAGCACAATGTCACACAGACTATACTGGCGGCACCAAAAACTGCGTGATGCTACTAGTTATCAGATTCAGATAAAACCGCTCGATCGGTAAGTGCATTATTTGGTTCAGTTCGCAATCGCATCTGCCTGCAGCTTCGCGCCATCGTCGTGGTGTTGGCAGCGGACGTCGTATGGACGGAAGCCGTGCGCGAGGGCGGCGCGCCAGGCCTTGTCGACGCCGGTGACGGCGTCGCCGCACTCGTGGCCGCCCCACTTCTCCAGCGCGTGCACGATCCCCGCCGCGCGCCACGCGCTCATCACCCGCCTCGGCAGCCAGTTCTCGCAGGCGTGGAGGTTCTCGTAGGAGTCCGGCACGACGAGCGCCGGCGTGGAGTGGTACACGCAGTCGCCGCGGGTGTTCATGGGAGGGAACTGGGAGTAGGGCACGAAGTGGACGCCCGGCTGCGCCCTCTCCTGCTCCAGCGCCGTCAGCCCGTCGCCAACGAGCCACACCTTGCTGCTGTAGCTGCGAGACGGAGCCAGGTGACTCTGCAACTCCGGAGACAGCTGCTGCTTCAGAGTCTCGTGCAGATGTTCGTCCGCCACACGAACCTGAATTTCCCGTTCGCACAGGGCCTGAGCCAGCACGCGCACGACCTTGCTCTCCTTTCCGAGCAGGAGCACGTCCTTGGCACCACGAGGGATCGTGTGGAGCACCGCCGCGACGGCCAGGCTGGTGCCGTCGACGATCTTTGTCTTCAGGCCGGGCTTCCTGATCACGTACAGCTCCCCGTTCCTGTTCATTTCGTACCGCTGATTCAAGAGGCCCAGCGTGAGGACTGCGGCGCCACTTGCCTCGGCGTCGGACACCGCCTTCTCGATCAGCCGGCTCACCGCGTACCCGTCTTTCTTCGACGTGTACTGGGAGGAGTATCTGGGAACGACCCACGTCTCGGTGCTGAGCTTGCCCATCCTGTTGGCCTCCGACCTGAACGCCGTCCTCCCGAGCACCGACGCCAGCGCGGCGAGCGGCCGCTCCACCAGAGCCAACGCCGCCGTGAACGAGCCTGAGAAGAGCGGCGCTGACGCGACGGACGCGAAGCCGAGGCGGGTGTGGAGAAGAGAGCCCGGCGCGGTGAGGTGCGTGAGGTGGACGACGTCGGGGGCCTCCTCCCTGCCGTGCAGCGTGCGCTCGTACAAGTCCTCGCTCGACTTGTCCATGGTCCCGTACAGGTAGTCGTAGAAGGGCACGAACAGCGAGTAGTTGGTCCTGAACTGCGTGTGGTGCAGAGAGTGGAACGATGGGGTGTACATGAGAAATTTCAGAGGGGGGAACATGTCGAAGAGGACTTTGGGGACGAGCTCGAAGTTGCAGTGCCCCAAATAGTTCATGAAGTCGATGTAGACGAGGTAACCGTTGGCGACGGCGACGGAGCCGGTGCCGGTGGCCATCATGGTGAGGAGCGGGATGGCGAAGAGGCCGAAGTAGACGGCCTCCTCGGCGAAGGGGTGGATGACGGAGGTGATGGGCTCGGTGACGATGGAGGCGTGGTGGTGGGAGTGGTACCGGGCGTAGAGGTAGTGGTGGTGCAGCGCGCGGTGGATCCAGTAGTAGAGGAACTCCACCGGGCCGAGGTGGAGGAGCACGGTGAGCACCAGGCCGCCGGAGTTCCACCACGGCAGGCCCTGCGTCATCGCCATGAAGGAGTTGACCACGTAGTACAGCAGCGCCGTCAGGATGATCTGGTCGTCCCAGTTCCTTTCACGGTCGACCTGGTCGAAGTCGAGGCTCTTGCTGACGATGCGGTGCTTGGAGCGGGCGGTCTGGTGGCGGGAGATGGAGATCCAGAGCTGCGTGTAGAGCAGCCGGAGGAGCACCGTCGGCAGCACGAAGAAGCTGAGCAGGTCGCCTTCCTCGCCTCGCACCTTCGCCGCCGCGAAGCTGTAGGTGCTGTACGCCGCCCACGGAGCCACCAGCGCGTACTTGTAGTTTCCGAGCTCGTGCCATGGCCACCGGGTGAGAGGCCCTGGCTTGGAGGCCATCTCGTTGGATACCCCTGTTTGCTTGGGAGATGTTTGTGAGCTCTTCGTTGCTAGATGGCACTTGCAGAGCTGCAATGGGGGATGGCCATGGCAGAGGAGTGCTTGCTTGGATTTATAGTAGTTCTC

1. Gene ID: DN85366_c0_g3

GAACTTTACTGCAAACTTGGCTTGCCATTTGTAATGGGCACAACGGGTGGAGATAGGCAACTGTTGAACAAAACAGTGCAGGATGCGAATGTTTATGCTGTGATATCCCCACAAATGGGCAAGCAGGTTGTTGCTTTTCTTGCTGCCATGGAAATCATGGCTGAGAAATTCCCTGGTGCGTTCGCAGGTTACAAACTAGAGGTCATGGAGTCTCATCAAGCGACAAAATTGGATGTTTCTGGAACTGCCAAAGCTGTAATCTCTTGCTTTCAGAAGTTGGGCGTTTCATTTGACATGAACGAGGTAAACTTGGTTAGGGATCCTGAAGAGCAGATGGCCATCGTCGGTGTCCCAGAAGAACATCTTGCAGGGCATGCATTTCACAATTACCATCTCACATCACCTGATGAGACAGTGTCGTTTGAGTTCCAGCACAATGTATGTGGCCGTTCAATATATGCAGAGGGAACTATTGACGCTGCCATGTTTCTCCGTAAAAAGATACAGGATGGGGCGAACAAAAAATTATATGATATGATTGATGTCCTGAGAGAGGGCAACATGAGATGAACAAGGCTATTTTAGGGTAGTACTTGTGTAGCCCAGTGATGGTGTTGTATAAACTCTTGTTTTGCGGCAAAATATATTAGGCCACTGCCGAGCTGTAGCAGTTTTGCGTCCTAATTGAATCTTAGGTTCGTTGAAACAAGTTAACTCTTTAGTTGTGGGCAAAAAGGCATTTGGACAAACTCG

1. Gene ID: DN78281_c0_g1

ATCCTCGCCATGGCCGCGCGCGACGTCGTCTCCCTGACCGGCGGTCCGAGCTACGGCGTGGAGCTGGGGCGGCTGGACGGCAGGACGTTCAACAGGGCCATCGTGAAGCACGTCCTCCCCGGCCCGGGGTTCGACCTCAACCAGCTCAACACTTTGTTCGCCAGCAATGGCCTCACGCAGTTCGACATGATCGCGCTCTCAGGTGCGCACACAATCGGGGTGACGCACTGCGACAAGTTCGTGCGCCGGATCTACACCTTCAAGCAGCGCCTCAAGTACAACCCGCCGATGAACCTCGACTTCCTGCGGTCGCTGCGCAAGGTGTGTCCCATGAACTACACACCCACGTCGTTCGCGATGCTGGATGTCTCCACGCCCAAGACCTTTGACAACACCTACTTCGACAACCTCCGCTACAACAAGGGCGTGCTCGCCTCCGACCAGGTTCTGTTCACCGACCGCCGCTCCCGCCCCACTGTCGAGCGCTTCGCCGCCAATTCCACCTTCTTCAACGAGGCTTTCGTCGCAGCCATGGCCAAGCTGGGAAGGATCGGGGTCAAGACCGGCAGCGACGGCGAGGTGCGCAAGGTGTGCACCGCCGTGAACTAGGACTAGGTAGCA

1. Gene ID: DN89118_c1_g1

CGCTGACTGAAGACTCTGTAATTTGTTACTGCAGATGACATGGAGAAGAACTCGCACCTCAAGGTCTTGCAAGCGCTTGGCCCCTTGGAGATCATCTGTGCCGAGCTAGACATCGAAGGCAGCTTCGATGAGGCAGTTTCCGGCTGCGACTACGCCTTCCTCGTCGCCGCTCCGATGAACCTTCGGTCGCCGGATCCTGAGAGGGACCTGATCGAAGCCGCCGTCGATGGAACTCTGAACGTGCTAAGGTCGTGCGCGAGAGCGGGGACGGTGAAGCGGGTGATCCTGACGTCGTCGGACGCCGGGGTGTCCAGGAGGCCATTGCACGGCGGTGGGCACGTGCTGGACGAGAGCTCCTGGTCGGACGTCGAGTACCTCAGAGCAAACAAGCCACCAACTTGGGCGTACGCGGTGTCCAAGGTGTTGTTGGAGAAGGCGGCGATCAAGTTCGCGGAGGAGCACGGCATCAGCCTCGTCACGGTTCTGCCGGTCGACCTGCCCGTCTTCACCTTGGGCGCGGTGCCGGTCTCAAAGGCCAGGACCAGCGTCCCCGTCACCCTCTCCCTCTTGTCCGGGGACGAGGCGCAGCTAGAGATCCTGAAAGGCCTGCAGTCGGTCACCGACTCAGTGTCGATAAGCCACGTCGATGACCTCTGCCGCGCCGAGGTATTCCTCGCCGAGAACGAGCCATCGTCGGGGAGGTACATCTACTGCAGCCACAACACCACCGTCCTGCAGCTCGCCCGTCTCTTGGCGGAGAAGTACCCGCAATACAATGTGAAGCCCAAACGGGATCGAGCCTCACTGCCTGCCTGCCTGCCTCAAGTCAACGTTTCACTTCGTGAGGAGAGGTTGTGACTAGTGAGTAGCGGCCGGCGATCGATGATGTCTTTAGATGGCGGGAGAAGGAAGACGGCGTGTGTCACCGGAGGGAGCGGCTATATCGCTTCGGTGCTCATCAAGACGCTTCTCGAGAGGGGCTACGCTGTCAAGACGACCGTCAGAGACCCCGATGACATGGAGAAGAACTCCCACCTCAAGGACTTGCAAGCGCTTGGGCCCTTGGAGATCATCCGTGCCGAGCTAGACATCGAAG

1. Gene ID: DN85886_c0_g5

TGGCCTCCGACGACGCCGACCCCATGTTCACCACGGCGCCCCTGCCGCGCTCCACCATCCCCGGCAGCACCGCGGCGGTCACCTCCGTCACCGCCCACAGGTTCACCTTTATCATCCTCACCCACGCATCCGCGTCCGCTTCGTGCAGGTACCTCGCGCCCGGCTTCGCCACGCCGGCGTTGTTCACCAGCACGCCAACGTCCAGCCCCTCAATGGCCTCCTTCAACCGCGCCATCGCCTTGTCACCTTGAGGGGTGGCGACGCTGGAGAGGTCGAACACCACGGTCTTGGTCTGCACTCCGTGGCGAGATCTGATGGTGTCGGAGATTTCCCGGAGATTGGCGGCGTTGAGGTCGACGAGGACGAGGTTCATGCCCTGGCTGGCGAGCTCCAGGGCGAAGGACCGGCCGATGCCGGAGGTCGGGCCGGTGACGACGGCCCACGCGCCGTACCGGCGGCGCAGGTCCTTGGGCCGGCGTAGGCACAGGGAGAGGTGCGCCACGAGGCGGAACAAAAATGGCGCGACGTAGAGGGCGCCGATGACGGCCAGCGACACGAACCATGCCGGCGGCGCTGCGGGCTGATCTTGCCGGAGGAACGATGCCATGCTCGCTTGTCTGTCAGTAATGCTCGAGCCAGCTGCTGGCTGCTGAGCGTTAAATGAATC

1. Gene ID: DN85366_c0_g1

CTGCTGCTCTGTACGCCCACCTTCCCCCAGAATCTCACGCAGAATCGCAGATGCTCTCTGCCGCCTTCGCCGCTCACCCCGCCGCCGCCGCCGCGCTGCAGCGGCATTCCGTCCGCCCGGGCCAGCTATTTTGGGCACCAATCGGCGCGGCGACGCCAGCGCGGCGGTCACGAGCGGCCATGCTTTCGGTGAAGAACGCCGTCGCTAGCCAGAGCCCTGTGACCGCCCGCGCGGCGCGTCGCGAGCTCTCTTTCCCGATACTGGTGAATAGTTGCACCGGCAAAATGGGGAAGGCTGTTGCTGAAGCAGCTGTATCTGCTGGTCTTCAGTTAGTTCCCGTGTCGTTCAGTGCAGTGGAGGTTCCTGATGGAAAGCTCAACATTTGTGACAGAGATATCC

1. Gene ID: DN92896_c0_g2

CCCAACCATCAAACATTTGCCTTCCTGATGCATAACACAATTTGTCAAGGTACTAATCAGCCACACTGAAGAAATTTTTAACCACAAGCATTCAAGATACATTATATTAGAAATGGGCCAAAGAGGTACCAGAATGCTTAAATTCATTTAAACGGACAGCACCCTTTTTACCCCACCCTCAGCTCTCGCACTAACTAACTAACCTCTTTATTCAAGAATCACGCCACAGTGAATAAGGAAAACAGATCTCTCTACCAGAACAGCTTCTACTATGCCACTGAAGGGCTGTCAATAGCAACAGCCATCGTGTTTAGTCCGTTGTCGACATAGACAGTCGAGCCGGTGATAGCAGAGGCCAAAGGAGACACCAGGAATGCTGCTGCGTTCCCAACTTCATCTGCCAGAAGTTCCTTCTGCAATGGTGCATTAACGTAAGAGTACTCGATCATCTTCTCGATAAATCCAATTGCTTTTGCGGCTCTGCTTCCCAAAGGACCTGCGGATATGGTGTTAACTCGAATTTTGCCTTTGCGTCCAGCTTCAAAAGCAAGTACTCTTGTATCACTCTCAAGAGCTGCTTTAGCTGAACTCATGCCACCACCATATCCAGGAATCGTTCTTTCAGATGCAATGTATGTTAGCGAGATACTAGCACCACCTGGATTCATAATAGGAACGAAGTGCTGAAGTAAGGAAATAAAGGAGTAACTGGATGCTGACATTGCAGCAAGATAGCCACTTCTTGAGGTTTCCAGCAAAGGTTTTGTAACCTCAGGACCATTAGCAAGAGAATGCACAAGAATGTCGATGCTACCAAAATCCTTCTTGACTGCTTCAGCAGCTTCCTTCACAGTCCAATTTGATGATCCTGCGTACCTTTTGTTTGTTTTGACATCTTCAGGAACATCCTCCGGGCAATCATAGACAGCGTCCAGTGGATAGACTTTAACAATCTCCATAAGAGATCCATCGGGCAGCTTCCGTGATTCGTCAAACTTTCCACGCCTCAGGCTTGTCTCGAATATGTTAAGTGCAGGCACCCATGTACCAACAAGAATTTCAGCACCAGCTGCTGCAAGAGCCTTTGCAATTGCCCAGCCATAGCCATTATCATCAGCAACCCCAGCAATAAATGCCTTTTTCCCTCTGAGATCAATGGGTAGCCCCTGAGGGGCACCTTCTCC

**List of sequences of identified genes in Table S10.**

1. Gene ID: DN70701_c1_g2

CGCGCCATAACCTTAACCGCATCGATCGTAGCTAAGCAAGCTTTCAAGGGAGCCGAGATGGAGGGAAGCAAGGGGGTCGTCCTGCTCAATTGCTTCGTCAGCCCCTTCGGCAACCGCGTGCGCATCGCCTTGACGAAGAAGGGCGTGGAGCACGAGGTGACCGCAGAGAACATGGCCCAGAAGAGCGCGCTGCTGCTGGCCTCCAACCCCATCCACGGCAAGGTCCCGGTACTCCTCGTCGCCGGCAAGCCCATCTGCGAGTCGCAGGTTATCTTGGAGTTCATCGACGAGGCCTTCTCCACCACCGGCGAGCAGCTCCTGCCCACCGACCCCTACGCCCGAGCCCAGGCTCGCTTCTGGGTCGCCTACGTCGATGCAAAGATTCCGGCCTGCGCCCCGAAGATATGGCAGTCGCCCAAGGGAGCGCCGGCGGTAGAGGAAGGGAAGAAGGAGATGCTGGTCGCGCTCAAGACGCTCGAGGCGGAGCTGGGCGAGAAGCCCTACTTCGCCGGGGAGTCGCTCGGGTATGCGGACGTGGCGCTGGTGACCTTCGCGCCATGGTTCCTCACGTACGAGCGTCTCGCCGGGTTCAGCATCGCGGAGGAGTGCCCGGTGCTGACTGCCTGGGCGGCGCGGTGCGCCGAGGAGAACGAGTGCGTCTCCAAGTCGCTGCCGGACCCAGAGGCTGTGTTCCAGTTCGTCGGAGGAATGAGGAAGCACTTCGGCCTCGAGTAGCTTTGCTCGGCTACCTACGAAGCGCGTCACTCTATTATATGAATGTATGATCCATGCAAAATAAGTTAAGCTCATACGCATTGTCTCGTGCGTATGTGTGTGCGCCTCATACGGATAATGTTTCCTCGCCGTCCGAGTCGTCTGTGCTTTTTATTTGGAGAGGCTTTGTGTTTTCCGAATTGATGGTGATCGATTTAAACTTTCTTGGTCGCGTCGTTGATGAACTGTACTCTGCTTGAAGAGCTGTGTCTTTTCTTGTTGATTATATATGT

1. Gene ID: DN50518_c0_g1

TTCAGCATGAGCCTCTTTTACAAAGTCCAACTTAGCGATATTATCTTTGTATCCTTGCAAAACCTTGAAAGGTGCCATGTCAACTCCAACATAACCTGGCCATTGTAACATAATATATAAGTACGAATCAGCTACGGTAAAATCATTTCCAACTAAATATTTCTTTCCACCACTCAACAAATTGCTTTCCACAAACTTTAACTGCTTGACAAGTTTCTCTCTCAAAGGTACTTTTTCTTCTTCAGATTTTGCATAAAATAATGGTCCAAAAGCTTTATGAACTTCAGAAGCGAGATATCCAAGAGTATTGACAGTTTCAACTTCTTCTTTTGCGCTTTTTCCAAGTAAATTGTTTTTACCGTTCTTGCCAACCCAATACAAAGTTCCAACGTTCTCATTAAGTAAGGATCCATCTTCTAAAACAATGGTTGGAACATTTCCTTTAGGATTCACTTTGTAGAAATCTGATCCATCCTTGATTACTTTATGGGTTCCTAAGTCCACAATATTCATCTCATCGTACTTGATCCCAGCTCTTTTGCTTGCAATGAAAGTTGCAGCACCACAGGAAGTTGGAGTGAAGTAAAGTTTCGGCATCTTTATTTAGTTATGACTTAAATGCAAAAGCGAGCGTTCTGTTTTGTTTTGATTTGTTTTGTTAAAATTGCTTTTTTGTTTGTTTGAATTAGAGTTTGAATTTGGTTTTGGTTTT

1. Gene ID: DN53663_c0_g1

AATATAGCTTTATATATCTGTTCGCCAAGAAATAGCGACTACTGGCGTAGTTACTCATTCAAATATATAACCTTGGCTCTATCAAATCCGTTAAATTTCGATGCTGCCGCAATAGTATAAGCACCCATGTTCTCGAAATACAACCATTGGCCAATTTCCAGTTTGGGAAGGTCAACGTCCCTAGCGATGCAATCAATGCTGTCACAGGTATGACCCCAAACACTAGATTTATAAGTAGCCATCTCATCGGGTGTAGCATCCTCCTCTAACTCCAGAACTGACGGGGTGACATGTGCATGATCATAAATAAGATTATTGAAACTGCCATAAACGCCTTCATTGACATAATACATCAACGAGTAGTCAGTAAAAGTTTTGCGCTCATCCTCAGAAGAGTCTGTGCTATAGGCGTCATCCCGTATTATTGTTTGGACTTCGCATGTCTCACGTCGCCCTATAATGTTTGCGGCCAAGGTTCCGCAAGAATGCACAAAGTAACGACCAGGTTCCGCAATAAGGCGAATGCCCGAATCTCTTGGAAACAAGTTATCTAACACCTGATTCATCTGGTTGGCGATGTCTGGAAACAACAGTTTACCCGAGTTTTCGGGTTGATTCCAGCTCGCAACCGAATCTCCAGGAAACCCACCACCTACATCAAGGATATTCCATTCAAAACCCTCTTCACGTGCATATTCGAAGGCACGATGCGCGAGGTAGATGGCATCGATGAAAGCCGCGCCATTGAAACACCCAGACCCTACATGGTAGCTTGCTCCGCAAACGTTCAACTCAAGCTCCTTTGCAAGACGAAGCAAGGATGGCACAACCTCAATATCGGCTCCAAACTTTAAACCAAGTCTGCAAACAGAATGGGTGTCATCTGGGCGGATCCTTAACAAAAGCTTTGCGTGCGGACATGTTTTCTTGACCTTGCGCAACTCATCGGCGTTGTCAAAAGTGAAAAAAGTAACACCCTTTTCTGTTGCGTGTCTCAAGTGAGTATATTGTTTGCAAGGGTTTGCATAAATAATTCTGTCCGGATGAACCCCGCACGAGATCACTTGATCAATTTCAGCACGTGAAGCACAGTCAAAGTTACTGCCCAAATCTGCCAAGAGTTTAACAATCTTTGCGTCTTGGTTACATTTAACTGCATAAAATGGTTCAACACGTGGAAAATATGTTTTCCATGCTCTAAATTTCTCACGAACGTCTTCTAGATCGACGACAATAAAGGCTTCCTCTTCACCTTTTAGGACAAGTTCCTTGACAACTGCTAACTTCGGATCAACGTACTTCATCGAGTATCTGAACCTGCAAATTCACAGAACAAACAGCCCACCCTCTCTGCTTTTTTTTTTTTTAACTCTTTTTTAGGTCTTTTTCGAAAACAGAAATAAAAATACTGCGCCTTTAATTCCCTTTTTCTCCAATAAAATTTCCCTATGAAATACTATTTCGTTCCAAAAAAATAATAGTCCTTAGAAAAAGTTATA

1. Gene ID: DN53607_c0_g1

AAAAAATTTTATTTTTTAAAATTAATAAAACTAAATTGAAACTTTATAACTGAATGAGAAAAGTTATAGCAGGATTTTTAAAGCGGCATATTGTAGTATAAGTAAAACTTGCTGCTATATGGACTTGCGCCAGCTTGGCTTACAAGTATTTTGGAGGAGCTTACTTTCGTTCCCAGCGCGCCCTTTTCAAGCAACTCGTTCAGTTGGTATGAACCAGATATTCAAATTTACTAGTGCGTCTTCTCGTTCGATGGCTACTACCCAGAAATCCGCTGCAAACGCGCATACTTCTATGGATGAAACTACTGATGGCGCATTTATCCGCAAGGATAGTGCTTTCAGGAGTTGGGTACGGGCTTCGCCGAAAGCTCAGGGTGAATACCCTGCTGAGGCTGGTCGGTACCATCTTTATGTATCCTATGCTTGCCCTTGGGCTTCTAGATGTTTATTTGCTCGAAAAATCAAGGGCTTGGAAGATGCTATTAGCCTGAGCGTTGTGGGCTCTGTTTTCCAGAAAACGTCGAGCAACCCCAATGACAGGCATACAGGTTGGATTTTCTTGACGCCAGAGGAAGAACCCGGGTGCATTCCAGATACGGTAATGAACTGCAAAACCATAAGGGAACTCTATGAGAAATGTAATGACAAACTGGGGAAATACACTGTCCCGGTACTATTTGATAAAAAGACGAAAATGATTGTTAATAACGAAAGTTCCGAAATCATTAGGATGTTCAATTCTGAATTCAATGCGTTTGCAAAGCATCCGGAAATTGACTTCTGTCCTGAAGACAAACGAGAAGAAATTGACAAAGTGAATGACTGGGTTTACCACAATATTAATAATGGAGTTTATAAAGCTGGATTTGCGCAAAAACAAAAACCATATGACCAAGCAGTTAATGCTGTGTTTGCCCATCTCGACAAAGCTGAACAGATTTTAGCAAAACAGAGGTATCTTTGTTCCAAGGACGCGATAACAGAAGCTGACATCAGGCTCTTTGTTACGATAGTGCGATTTGATGAAGTATATCATGGACACTTCAAGTGTAATAAGAAGCGACTTAGTGACTATCCAAATCTGACAAATTACATGCGAGAAATATATCAGATGGACGGAGTTGCTGAAACGGTTAATATGGAACATATAAAAAGTCATTATCATCGATCGCATCCGTCCATCAATCCGTTTGGAATTGTTCCTATTGGACCTGACGTATTGTCAAGTCTTACGCTACCACACAATAGGAGTTCTCTATAAATGTGTTGTTCGTAGCTAATTATTATAAAAGAATACAGAATTCGGACATATAGA

1. Gene ID: DN77185_c2_g1

AACACAAACACCCGCCATTATTCCAGGTGAGCTCTCTGAAACAATCGAGAACGAGAGAGGGAAGCGAAGATGAGCGAGCCGGTGAAGCTGTTGGGCTCGTTCGGGAGCCCGTTCACGCACCGCGCGGAGGCGGCGCTACGGCTGAAGGGAGTGGCCTACGAGTTCATCCAGGAGGACCTGGGCAACAAGAGCGAGCTGCTGCTCCGGCACAACCTGGTCCACAAGAAGGTGCCCCTGCTCCTCCACGGTGACCGTGCCGTGGCCGAGTCGCTCGTCATCGTGGAGTACGTTGACGAGGCCTTTGAGGGCCCGCCCCTTCTCCCTCTCGGAGATCCCCTCGCCCGCGCCGCCGCCCGGTTCTGGGCGCAGTTCGCGGTGGACAGGTGCTCGAGGACGCTGTTCAAGGCGCTGTGGACGCCGGACGGCGAGGCGCGGAGG

1. Gene ID: DN62218_c0_g2

AAACATTTGGCCACTCCCGCGCTTTTCCTCCAAATCGCGCGCGGTTCCCCCAACCTCCCACCATATACCACTTCTCTTCTAATTTTCTTTCGAAATCATCCGCACGCGACGATCCCTCCACCTCCAGGCCGTTCCAAATCCCTCGCCGCCGCCTCCGCCTCCGCATCCCCCAATTCCCGAACCACCTAGCGCCGACGCCACCTCCCCGCTGCCGCGCACCGTGCCCGTGACGTGCTCGACGGAAGGCCCGCCTGAGCAGCCCAAACCGGCAGGCGGCGGAGATGTACGTGGTGAAGCGCGACGGGCGGCAGGAGGCCGTCCACTTCGACAAGATCACGGCGCGGCTCAAGAAGCTCAGCTACGGCCTCAGCCAGGACCACTGCGACCCCGTCCTCGTCGCCCAGAAGGTCTGCGCCGGCGTCTACAAGGGCGTCACCACCAGCCAGCTCGACGAGCTCGCCGCCGAGACCGCCGCCGCCCTCACCGCCTCACACCCCGACTACGCCTCGCTCGCGGCCAGGATCGC

1. Gene ID: DN76086_c0_g1

GCTATCTTTGAGAAATAGATGGGTGTGATGTTGTGGATGCATCTAGCTTCCACAGGCCAAGCACTCTTCCCGGTTGTTCAAGGAACAGACGACCTGTGCCATTTTGGCCTCATCATCTTCCTCTGCTGGTTTGACATCAGTGTCCCCATTCACCTTCAAAAAGCCTGTATCCACTGTAAACTTGATGGCATCAGCGGCTGCACGTGTTCTTAAATAGTACATCCCAGTTTTCAGGCCCTTGGACCAAGCATGGAAATGCAATGAAGTTAGCTTCGCAAAGTTTGGTTGGTCCATGTGAACATTAAGGCTCTGACTCTGATCAATGTAGCATCCACGATCAACAGCCATGTCAACCAACGTTTTCTGCTTGATCTCCCAAACAGTTTTGTAGATTGCCTTAAGATCATCAGGGATTTCTGTCATCTTTTGAACAGAACCATCTTCATAAATAATCTTGTTTTTCAGAGCAGGTGTCCAAATGCCCATCTCAGTCAAATCATGGAGAAGATGCTTGTTAACCACCACAAACTCTCCACTTAAAACCCGTCGACTGTATATGTTAGATGTGTAAGGCTCAAAGCACTCATTGTTGCCAAGAATCTGACTAGTGGAAGCAGTGGGCATTGGAGCAACAAGAAGAGAATTTCTCACTCCAACTTTAGAAATAGTCTCCCTTAAAGCTGGCCAGTTCCATCTGCTCGATGGCACTACATTCCACATGTCAGGTTGGATAATGCCCTTGCTGACAGGGCACCCTTGATATGTTTCATAAGGACCTTCTTTAGCAGCCAGTTCAGCAGAAGCTTTCAGAGCATGATAGTAAATAGTTTCAAAAATATCCCTATTCAACTGCTGAGCCTCAGCTGAATCAAATGGCATGCCAAGTAACATGAAAGTGTCTGCCAA

1. Gene ID: DN91181_c0_g1

CTTGAATACCGGCTTCAACTCTTCAACCAGAGCAAACAGACATCTGGCCGCCATGGCAGGGCTTGGCACTGGGGCAGGAGCTCCTGTCGTGAAGGTGTACCATGAGAAATCCATGATCCTGCCCGATGTGTCAAGGGTGCTTGCTTGCCTGTATGAGAAAAATGTTGAGTTTGAAACTGTGAAAGACTCGTACAAGGACATACTCAGACTCCAGTCAACGAGGAGCGTTCCAGTTCCATTCTACGACGGACCCATATTTCGACAAGAATCAAGAGCAATCTGCCGCTATATAGCAGAAACATACGAACAGCGTGGCTATCCTTTCCTCCTGGGAAAGGATGTCCTTGAGAGGGCTTCCATTGAACAGTGGCTACGGCATGAGGAGCATGCCTTTGATCCTCCAAGCAGGGCATTGTTCTGCCATCTGGCTTTTCCTATGCTTGATGAAGACATGAGTGATATCGACAGAGAGAAGAGAAAGCTGGAAGAAGTCCTGGAGGTCTATGAACAAAGGCTTGGCGAGAGCGAGTTCCTTGCTGGGAACAAGTTTACTCTTGCTGACCTTGTTCACCTACCAAACACTCATCACATAGTGACATCAGAAAAGTTCGCTTACCTGTATGACTCAAGGAAGAACGTGCAGAGGTGGTGGAATACCATCTCTGCCCGAGATTCTTGGCAGCAGGTGGTGAGGGATATGCAAAGTGTGGAGGAGCAGAACCAAATAGAAGAACTTGAGCAGCAGCAGCTGGAGGAGCAGTGGCAATGGGAGACAGAACCACCACCAACATCTGGTCGCCGCATCTTGCGCATAGACCCTCGACAGCAGACCGGCACCGAGTCGCGAACAGTACTGGTTCCACCACCCAGCGGCGGTGCCATATCACCATTGTCTTTTACAGTTGAACAGGAACAGCAACCTCTTCACACAGAAACAACCTCTCATGGTGAAACTTCACCTAACCACAGAAAAGGAAGTAACTTCTTTACTACCACCGATAAAACTCCAGCGCCCTCAAAGCAAAAAACCTCCACCCCTCAGAAACCACCTAGCAGTGTTGAAGGCACTAGGAGTAACTTCTTTACCCCAACTAGCCCTCCTACCTCCACCAGAGTTTCTTCAAAAACTAATAATGAGAAATCCACCTCCAAAGATGCCTCAAAAACCTCTGACAGGGATTTTCAAGCTCGTCAGACTGGTGAAGTAGTTGCTCCCCATAAACTCCATTCAGGATCACACAAAGCCCCCCAAGAAAATACTCCTGATAAATCCACTGAGAAATCACCTAGCAGTGTCCAAAACACTACGAGTAGCTTTTTTACTCCTCCTGCCACCACCAAAATGCCTCAGACAACCAATACTGATAAATCCACCTACAAAGATGCCTCATCTCCAACCATACCCAGTCAAGGATCATCCGAAGACAACATTAAGGAAGCTAGAGACGCTGATCAAAAGAGGTCTGCATCAGCAACAACCAGGGAGCTACCATCAGGTTCTCAAAATACCCCTCAGCAATCTAAAGCATCACCTAATCTGAAAGCTTCTGACTTGTCACCAATGCAAGAAGAATTTGAAGATACTCAGGGTGAAGATGAACGGTTCTCAACTAAGAGGCTCAGAAAAATGATTCAGCAAAGTGATCCAGAAGTACTCAAACCACAGTCTACAGATTTGCAAACCCGTCCAACACAGGAGGAAACACCTTCCATTTCTAAGAAGCCTTTGTATGTTCAGGACAGAAAAGGGCAAGCTGGTAACAATCCGACTGATGGAAAAATTGATGGTATTCCATCAACTGGCACAAGAGATCCTGGCACTCCATCATCTGGGACAAGTGATCCTGGCACTCCAACTGCTGCTGATGCAAGGAGAGCAACTACACCACCAAAAGGAGGAGTTGATCCTGATGGTCGTGGTGCCACTGAGCCACGAAAATTACAATCCATCAATGAGCAACAACCAGCTCCACCAATGCCAAACCGATCACCTATAAGTAGTGCTCGGGGTGCTTCTGCATCATCAAAAGGAGCTGCTCAAGATGATGACTTAGCCCAGTCAAACATTGATCAATGGAGGCACACGTCTACTCCTGCAAACCAAGGAGGAGTTCCAGATGCTGCTATCACTGATAAGTTGGACAAAGCAGCAGACAAAAGAGCACAGACACAGGCTCCAAACAAAACCTCGACCGGCCAGTCGGACAAGATCGCTATGCAGACACCACGACGTACAACTACCACATCAGACCAAGCTCCAGTGTCACCTTTTTCTGCTGATAGAAGGAAGACAGGTATTGATGAAGCCAGTCAAACTACCAAGGTAGCACCTGATGAGCAGCCTGCTGGGAGAGCTCCTACGAATTCTGTGAATAAGCCGATATCTCCCAAGGATAATGGCAAGTTAACTAATGAAACCGTATATTATAGCAGCTCAGAAATATCCAGAGAAATGGCACCACCAGGCTCCGAAAAGAGCATGGGACAGCAGAAAATGCAAAGTGATAAGTCCAGTATATCACTGCAAGCCAATGGGAAGCAAGGTTCTGAAGCTGCACTTCTCACAAATACTGATCAAAACACTGGGAAAACTTCAGGACAAGATTTACAAGAGTCTCAGAAACAAATCTCCCCTGATACTCAGCAAATGAAGAACAACAGAAGCAACAGCACATTGGATGGCTCAAGCAAACCCACCCAATTTGATGGCAATGAAGGTGATGTCCGTGAGACACAGAGAGGTGGCTAGTAAACCAAACTATAGCACGCCTCTGTCAGGTCATCTTGTTGCAGCTTC

**List of sequences of identified genes in Table S11.**

1. Gene ID: DN85177_c0_g5

GCCTGCATTGCATCAATGTCCAGGATAACAAACATGCTTATTATGTTCGATCACACGCTCATTATTAAGCAACATGCCATATATGCATATTCATGCTTTGCCGATCGCCATCAAACCGACCTGAAGGACTGGTTTTCCGCGGCTCATCCAGCCACCACCTATGCTGCCTCGGCAAACCCAACCCTCATTTTTCCATAGTCAAAGACAGTGTGGTAGGCTCCCATAAATATATCGCCCAAGATCCAGATAGGACCGCGGGGACGAGGAACGTCCATAGCTGAGAATCCACTGATGCACTGGGTAGCATCACCCTCACCGATCTTCAGTATGTATTGTTCTGGTTTAAGCACAAACTGTTTGCCTCCGATGGAGAAGGCGACGTCAGGCAAGGATTCAAGTTTTCTGCAGTCCACGGATGACTCTCCCATAGGGTTAGGAATATGGTCACATAGCTCACTGATGTACTGCAATATAGAATCCTGAGTGTTATTCTGAGCAATTTGGTTCATCGTCCATTGAACAGCCAGCTCACAAGCCTTGCACAAAAGAACACCTTCTGATGTCCCACCATCATCTTCTACGACACTTCGAATACCAGCGCTTACACCATCAGATCCACCAAGGGGGCATAAATCGACTAAGGCACATGTCTTTTTTGGCTCCGTCTGCTTTAGCAGCAAATCCAGGATCCGCCGCCCATACTGAGAAACAACGGTCTTGCATGCTTGGCTCACTATTCCAGGTGCACCAAGTTTATGATTTATCTCAGTAATTATCGCCGTGGGGCCAGTAAGCAACGAAGTTCCGGAGTCTACTATTGCTGCACAACCAGATTTACATAATCCTGTGGAGTTTCTTCCAATCAAGACATCGCCCATATCAAACTGCCAGTATCCCTTCTTAGTAACAGGGACATAGGTATGACTTCCCTTGTGATGATTAGGATCGACTCCTCCAAAAACTATTTCTCCTCCCTTCCCTTCACCAGCATGTCGGTTGAACCAGAATGAGAAAACAGGGCTACCAACCAGACCTTGGCTAACCATGTTATACCACACAGGTTCAGCACCGCCGGCCGAGATTTCTTTAAACCCAAGACCAAGAATACCGTCGAATTTTCCAAGCATGAAAGAAATACTTGGTTCCAAGGCAGCTTCAATAAAATCCTGATTTTTCACAACTACACCACCAACTTGCACACTGTCTTGGCTAATATAGCCAACAATTGCACCAGTTCCGTAATGAATTGATACCCATTTTCCTTTCTTCTTGTACGTGCTCGACCGTCTGGCCCTGTAGCTCTTGTGCAAGTAGCATGCAATCGAGAAGTAGCACTTGGAGGAGGGCACCCAGAGGTTGGAGCTGCCAGTGTCGAAGATGACGGTGAAGTTCTGCGACGGCGTTCCGATGCCGACCTCACCGTAGTACTGAGCGTTCAAGTAGTTCTTCAATATAACAATATCACTCTTTGGCTGCGGCTGCGGCTCCTTGTTAAAGACGAGACCGTGGCGCTGTGCGAGGAGGCTCTGAGCATCCTCTCCGGCGACGAGGCTGTTCTCGTGGACCAACGGTAGCTTCTTCAGCGGGATGCGGACCAGACCTTCCGAGGGCGTGCCAAGGATGAGCACGGCGGAGAGGATGAGTAGGTGCTGGAGCGCAACGCGGCCCGTTCCCATGGTTGCGCTGCGAGTCTCAACTGTGCTGAGGAAGGAAGGAGAAGACTGCCCTGCGAGCTTGTGGACAGTGCGGCGCCTTGGGTCCATTTATAAACTTCGGCCGGGTGGCTAGAACAGAGCTACATGATCATTTTCACCGTTGGCGGACTTCTGTGAGCCGTCAGATCAGGACGGGGTGATTCTATCCCAAGGTGGATTTTTCCCCTTTTGCTATAAGGTGGATTCACCCGTAAAATTTGTTGAAAGTCAATGTCTCCGTGCAAGTACTATGTGATCGATTGGAAAAGTGGATGCAGGAACATGCTAGACCACACTTGTGTCCACTCATGAAAAAGGTTGTGTAGTTTGTACCATTGCCTGAAGAAACAAAAATAGATTGCTTGTTAGTGGTGTATAACTAGTGTGCCACACGGTTCGTTAATCACGTATAGCATGG

1. Gene ID: DN77249_c1_g1

CGAGACAATATATTAACTTTGACCTGTGCAATAATGGTGTCGACCCAACGACGGCCAGCGGAGCACTGCTTGTTGGCGGTGTTTGTTCTTCTCCTCTGCGGCGACGCTCTGGCAGCCTCGCCGGCGCGCTACCTCTCCGTCAGCCTTGATACTGTCATCAGCTCCAAGGCTCAACTCAACTGCTATGACCCTGACACTACGATTCCAGCAGCATCCTCTGGCAATAGGCTCGCCATCCATCCGGCTTGCTCCGGCGGGTCCGAGAAGGCGTGGCGCCAGCGCGACATACTCGTGCACGACAATGCCCGGCTAAGCACTGTACTCCAGAGGTCGACCTCTTCAGTCATCACCCCGGTTTCACCCGCACCGGCTTCAGCGCCAGCAGAGGCGCCATCCGCAACCATCCCAGATCGCCCGGGGACCTATCTGGACACACTGGAGTTCGTCGTCATCATCGGCTTCGGCACCCCTGCCCAGTCCTCCGCTATCATCTTCGACACCGGCAGCGACGTGTCGTGGATCCAGTGCCAGCCCTGCACGGGCCACTGCTACCAGCAGCACGACCCGCTCTTCGACCCCTCCAAGTCCTCGACCTATAGCGTCGTGCCCTGCGGCACGCCGGCGTGCTCAGTTGCCGGTGGAAAGTGCAACGGCACCACCTGCCTCTATACCGTTCAGTACGGCGACGGCTCCTCCACCTCCGGCGCACTCTCTCAGGATACGCTAACGTTCACCTCCGCGCGCACCTTCTCAGGCTTCACGTTCGGGTGCGGCACTACTAACCTCGGGGACTTCGGCGAAGTCGATGGGCTGCTGGGTCTCGGCCGTGGCCATTTCTCGTTGCCCTCACAAACCACTTCGTCATTTGGAGGCTCCTTCTCCTACTGCATGCCGTCTTTCAACACCACGCCCGGGTACCTCACCATTGGCTCCACGCCTGTCACCGGCAAGGTCCAGTACACGGCAATGATCAAGAAGCCAGCGTACCCATCCTTCTACTTCGTGGAGCTCGTGTCCATCAACATCGGCGGCTATGTCCTGCCAGTACCACCAACCGTGTTCACGAAAACGGGCACCCTCCTCGACTCGGGCACAATCCTCACCTACATCCCCATGAAGGCCTACACCTTGCTCCGCGACAGGTTCAAGTTCACCATGAAGGGGAATAAGCCGGCGCCGGCTTTCGACATCCTCGACACCTGCTACGACTTCTCCGGCCAGGGCGCCATAGTCATACCGGCGGTGTCGCTCATATTCAGCGACGGCGCCATTTTCGACCTCAACTTCTACGGGATCATGATATTCCCGGACCAGACGCAGCCGGCCGTCGGCTGCCTCGCCTTCGTGGGGAGTCCCGCGGGGATGCCCTTCTCCATCATTGGCAACACGCAGCAGCGTTCCGCCGAGGTGATCTACAACGTTGGAGGGGAGAGGATTGGTTTTGTTCCAAACAGTTGCTAAATCCGGGGTTCATAAGTCACCAAAGTATAGTACTACTCAATTTTATCTTCTTAATTAGTACATGTGTGAGTATAT

1. Gene ID: DN108310_c0_g1

GAGCGAGCAAGAGAAACCTTGTTGTTTCCATAATCGAAAACGGTGTAATAGCTTCCGAGGAAGACATCGCCCAAAATCCATAGAGGACCAGCTGGAGGAGGGATATCAATTCCCATGAATCCAGAAATACAGACAGTTTGACCCAAAGTGGAGACCTTCAAGACGTAGTCAGCACCATGGAGAACAAAGTCCTTACCGTTGAGAGTGAAGGTAATGTCTGGGAGAGAGTCGATCTTGCTGCAATCCACACTGTATTCACCGTTGGCAATAGGGGTAGCTCCAATCTGTTCGTTGATCTTCTTGGTCTCGACAGTTGGACCAGCAAGGAGAGACGTTCCGGTATCGAGGATAGCTTGGCAGCCACCATCGCAGAAAGCACTGGATCCTAACTTGATACCGTCCATTTTGACTTGCCAGTATCCGTCCTTGGAAAGAGGAACATCACTGAAGTCACCGGTATAATGCTTAGAGTCTACATCACCGAAAACAAGTTCACCACCATCAGTCTCGGCAGCTTTACGGTTTAACCAGAAGGCGAAGGTTGGCTTGGAGATGAGCTTTTGGTCAACCATGTTGTTGAAAACTGGAGGAATGCCGTTGACAGAGATTTGAGGGAAACCCATGCCCAAAATACCGTCAAACTTGGCCATGACAAAAGCCAAACCAGGCTCTTTAAGAGCTTCAGCAAAGAGTTGGTTCTTGACAGCGAGAGTTCCAACAGTAACTGTATCTTCAGAAACAGTTCCTGAGAGAGAACCAGAACCATATTGGATAGCGAAGTCAGTTCCATTCTTTTGGAAGGTAGAGGAAGCAGAGCTATCGTACTTGCGATGAAGCTTGCAAGCAATGTTCAAAAGAGAGCACTTCTTAGATGGAACCCACAAGTTGGAAGAACCAGTGTCGAAGACAACACGGAAGGTTTGAGGAGGAGTTCCGATGGAGATCTCACCATAATATTGAGCGTCTTGGTAGTTGGACAAGTGAATACCAGGAACAGAGGTATATTTGTATGCAAGAGCCTCTCTGGCTTCAGCGAGATTGCGGTTAACAGAGTCAGCTTTGTGCAGTTTGACAACATTGGCGGTGGCAACTGCCACCAAAAGGGCAAGCAACAATACAACCTTCATCTTTTTCT

1. Gene ID: DN96587_c1_g1

AGGGCGCCGTAGTAGTCTGGGTTACCACTCTCGATGTCGAAGTCGACGCCGTCGAGAACAGCGTTGCCGAGGGGCCTCTTGCCTGACCCGCCGAGGTAGCTGTTCCAGATGTACTGGGCGAGGTCCGCGGCGTCCTGTTCGGAGTTGAGCGTGTACCCTCCGGCGCCTCCGCCGATGGAGAGCATGACCTTGACGCCCTTGGACTGGCAGGAGCGGATGTCGGCGGTGAGGTTGGTGCACGCGTTGGAGTAGGGGTCGCAGTGGCCGGCGAGGTTGAGCTGGGGAGACTGCCCAGAGCCGAAGCTGCAGAGGAAGGCGATGTTGACGAAGGCGTAGTTGCCGGTTGCGCATGTCTCGGCGAGGGTGCCCTCGTTGCCGTTCTGGCCCCAGTAGATAGCAATGCTGCCGCCATTTGCTGAGGAGAAGAGGAAAATTGCCAAGACGGCGGCGATGATCAGGGAGCTCCGAGGAACGGCCATTGTTGCTAGCTAGAAAGCTCTTTGGTTATATGCAC

1. Gene ID: DN76141_c1_g4

TCCCACTCGTCGGGGAATGGGCACTGCGGCGCAGCGCTCAGCAGCACCGTCGTGCCACCGTTCTTGCCCAACTTCTTCAGGTCCGTTGCGAGATTGTTCCAGAACTTGGCGCTGCCGAGCTCGATGTCGAAGTCGATGCCGTCGAGTACGGCGCCGCCTAAAGGGCGAGAGGAAGACGTGCCGCCCAGGTAGTTGTTCCATAGGTACATGGCGACCTGACTGGCGTCGCCAGGGGACGAGAGGCCGTAGCTCCCGTCGCCGCCGCCGATGGAGAGCAGGACCTTGACGCCGCTGCGCTGACACGACTGGATGTCCTTGCTCTGGCTTCTGCAGCCAC

1. Gene ID: DN79714_c0_g2

CACAAAGCACAGCTCACTGTCTCTCAGTAGCCTCTCTACACTCTTTCCTGTTCTAGCTAGGCAAGCTCCGGTAATTTATCCCAGGCCAGCCATGAAAAGCCGATCTCTCACCCCCTTCTTGCTCGCTGGCTCCCTCGTCGTGGCTTTCCTCGCGACGTGCCAGGCCGGCAGCATAGCCGTCTACTGGGGCCAGAACGACGGCGAGGCGTCACTGGCCGAGACGTGCGCGTCTGGAAACTACGAGTTTGTCATCCTTGCTTTCCTTCCCAAATTCGGCAAGGGCCAGACGCCGGAGCTAAACCTTGCCAGCCACTGCGACCCCTCGTCAGGTGGCTGCAGAAGCCAGAGCAAAGATATCCAGTCGTGCCAGCGCAGCGGCGTCAAGGTCCTGCTCTCCATCGGCGGCGGCGACGGGAGCTACGGCCTCTCGTCCCCTGGC

1. Gene ID: DN78248_c0_g1

TCTTGTTCACTGTACTGTGCTGTGCTGTATTACCTGATGAGTCGCCTCCTGCTCGTCCTCCTCGGGATTGTCTTCCTCCTCTCGCATGCAGCAGAGTCGGGCGAGGTGGGCGTGTGCTACGGGACGATGGGGAACAACCTGCCGACGCTAAGCTGTGGCGCACAGCTGCTGGTGGATAAGGGCGTCGGAAGGGTGAGGGTGTACGACCCCGACCCAGCTGAAATGCTGACGGCGTTCGCCAACACCGGCATCAAGGTGGCTGTGACGCTGCCGAACGAGCTGATCGCGGACGCGGCCGGTGATCCAGAGTGCGCGGAGGAGTGGGTGCGGGACAACGTGGAGGCCTTCTACCCGGAGACGCTCATCGAGAGCGTGTGCGTCGGGAACGAGGTGTTCAAGTCGGCGAGCGAGCTCACCCCGCAGCTCCTCCCGGCCATGGAGAACGTGTACAGGGCGCTGGACTCTGCGGGCTTGGCTGACGCCGTCAAGGTCACCACGCCGGTCGCGTTCGACGCGTTCAAGAACACGTTCCCGCCGTCGGCGAGCGTGTTCCGGGACGACCTCGCGGAGTCGGTGATGTGCCCCATGCTCGACTTCCTGGACCAAACTGGGTCCTACCTCACAGTGAACCTCTTCCCCTACATTGCCTACATAGCTCAGCCAGAGAATATCTCCCTCGACTACCTGCTGTTCCGCCCCAACAATGGCGTATATGACCGAGGGAGCAAACTAATGTACTACAACCTTTTCGACGCCCAGCTTGACGCCGTGTACTATGCCATGGACAAGCTGCTGTCGTCCGGCTCCCGCGCGAGGGCCAATGGGGGGAGGAAGCTGCTCCAACAAGGGCCTCAGGGCGACGCAAAAGTCGGGGAGACAAATGTCGCGCACACAGGACATAGTGGGAGTGGGCCGCCTCACAAGCAGGCCGCGGATGACGACGGCGGTTGCGCCGGCGCCGCCTGTGGGGTGACTACTGTGGAGAACTCCAAGGCGTATGTCGGCAACCTCATCAACAGAGTGGTCCAAGGCGAGCAAAAGACCGGCAACCGCGGCACGCCGTACCGCCCCGACGCCGACATCGACGTGTACATCTTCGCCCTTTTCAACGAGAACCAGAAGGAGGGGCCAGAGGACGAGCGGAATTTCGGGCTCTTCTACCCAGACCAGACGCCCGTGTACAACGTCGACTTCCAGGGCGGCAACGTCGAGGACACCAGGTGGTGCGTGGCGAACTCAGCAGTCGGGGATGAAAACCTGAACAAGGCCCTGAGCTACGCGTGCGGCCATGGAGCCAACTGCAACGCCATCCAGAGCGGCGGACGGTGCTTTAAACCTGACACCGCCGTGGCACATGCCTCCTACGCATTCAACGATTACTTCCAGCGCAACGGCAGGTCCAGCGTTTCATGTGACTTCAGCGGCGCCGGTTACGTCGTCTACCAGCAACCAAAGTTTGGCAATTGTGAGCTCCCATCAACACCAACTGATAAAATTTGAGGCAACACCAACAAAGGAATAAGCTACTTGTTTTCCATTTATCGATACTTGTAAGTGTGTGTCTTTGTGAAGAGAAAGACCAAACCACAATATACCAAAAATAAGATCACGTGGCTATACCCAACTGAGGTAACAAGAGCGTGC

1. Gene ID: DN73750_c1_g1

GCGTGTGTATATTGTTATTTATCTGTTTGTTACATGAAAAAATGAATAAATCGTGAGGGGAAATGAGGGCAGCGTATGCTAGCATGTGTGTGTGTACGCTGCCTTCTCCCTGGTGCTCTCCGCTCTGCTGAGGCAGGTGACCGACTCTGCAGTCTGCACTGAAAGTGCACCGCAGGGCGCTTAATTAACTGGATTTCTATTTCATGGAGCACTCCGGTGGCACGCCGTTGGGGAACCTCTTGGTGTCCGTGCAGTAGTTGTAGATCATGTATTCCTCACGCACCCGCTTCATCTGCTGCTTCGCTCTCCCGTCCAACTCCTGGTCGTAGTAGTCCCCGCCGGCCGGCGACGACGAGGTGGCTTTGAAGTTGCGGTACTGCGCGAGGAACGGCGCCGCCGACCAGTCCGTCTTGACGCGGCCGCCCTGCGTGGCCCAGTCGTCCGCGTTCCACAGGCTCCCGAACAGCCGCATCCGCTGCGACGACGGGTACGGGTACGCCGCGCCCTTCTTCGCGTAGTTCCTGAACTCCCGGATCGGCGTGCCGTCCACCAGCACGAGGATGTGCTTGGGTGTCCAGACGATGGAGTAGGTGTGGAAGTCGGCGGCGGGGTCGAACCAGAGGCGGAACTGCTCCTCCTTCTGGCCGGTGCCTTCGGCGAAGATGTTGGTGTGGAGCGTGTAGGGCTGCCCGCTCACGTTGCCCAGGAACTCCAGGTCGATCTCGTCGTGCTTCTCCCATGGCCCCTCCGACAGGAAGAAGAAGGTGGTGACGGTGCCGGCGGAGTTGTTGGGGACGAGCTTGATCTGCAGGTCGGTGCGCGCGTACAGGTACGTGTCTCTGGACCGGAGACCCGAGCCGCCGGAGTCCCGGTCGAGGTACAGCGAGATGACCTGGCCGTCCTTCGAAACGTTCCCGTGTCCCCAGTCCAGCTCTAGCCCGTCATCTGACACGTTCCCGTTGCCCCAGTCCAGCTCCAGCCCGTCGGCGATAATGCCCCCGGCCACCACCGCCGTCCGCGCGGAAATGCAGAGCCCCACCAGCACCGCCAGCAGGCAGGCCTCCGTCCTCGAGCTGACCGCCATGGCGTAAGCTTTGCTGCGCCTCGTCAAAGAGCTGTGTGTGTGTCTGTGATGGACGTGAT

1. Gene ID: DN77114_c0_g2

CGGCGGAACATGGTGCCGGTGCCTAGGTAGGAGGGCCCTTGGAGGCCGTTGAGGGAGAGCATGGTGCCGTCAAAGAAGACACGGTTGTGGTTGGCGTAGCGGTCCGTCGGGTCGACATCGTCGAAGCGCTGCGGGAACTGGACGAAGGCGGTGTTCTGGCCGTCGCGAGGGTCGAGCATGAAGCACATGGGAGCGCGGAGAGCTTGCGAGTTGTTGATGTAGTGGTCGCAGTCGAAATTGACAACGAAGGGCGCGTTGGAGAGCAGCGCGGAGACACGGAGCATCACGTTCATGGCGCCTGCCTTCTTTTGGTGGTTATAGCCTGGGCGCTTCTCGCGGGCGATGTACACAAGTATGGGGAGCCTCGTGTCAATGTTGCTGAAGTCGAATGGATTTTCGGTGCTCGCCCGTGACCCGAGTTGTGGTTTACAACTTGGATGGTTTAGTATGACCTGAACAATTCCAGCATGCTGGCCTCT

1. Gene ID: DN75875_c0_g1

AGAGCTTCAGAGGGCACAGTGGAGGAGCCAAGCAGGGACATGGCTTCCAGCTCACCGTCTCCTCCTCCTTCACCGCGGCTCCTCCCCCTCCTCGCCGTGGCGGCCGTCGTGCTGCTGCTCGGCCCCGGCGGCGGCGTTGAGGCCAGGCAGCCGCCGCCGCTCCACGGCGTTCGGCCCATGGCCTTCGACGAGGGCTACACCCAGATCTTCGGCAGCGCCAACCTCGCCCTCCGCGGCCAGGGCAAGCGCGTCCACCTCTCCCTCGACGAGGCCACCGGCGCCGGGTTCGCCTCCCAGGACCTCTTCCTCCACGGCTTCTTCAGCGCCGCCGTCAAGCTCCCCGCCGACTACGCCGCCGGCGTTGTCGTCGCCTTCTACCTGACGAACGGCGACAGGTACGAGAAGACCCACGACGAGCTGGACTTCGAGTTCCTGGGCAACGTGCGCGGCCGCGAGTGGCGGGTGCAGACCAACGTCTACGGCAACGGCAGCACCGGCGCCGGCAGGGAGGAGCGCTACGACCTCCCGTTCGACCCCACCGACGACTTCCACCACTACTCCATTCTCTGGACCGAGCACCGCATCATATTCTACGTGGACGAGACGCCGATCAGGGAGGTGGCTCGGACGGAGGCCATGGGCGCGGCGTTCCCCTCCAAGCCCATGTCCCTCTACGCCACCATCTGGGACGGCTCCGCCTGGGCCACCCTCGGCGGCCGCTACCGGGTCGACTACAAGTACGCGCCCTTCGTCGCCCAGTTCGGCGACCTCGTCATGCAGGGCTGCCCCGTCAACAGCCCCGCGGCGGCGTCGTGCGGCACGCCCTGGTACGAGGCCGCCGCGTCCCTGTCCGGCGAGCAGCGTAGGGCGATGGCGGCGTTCAGGCGCGGCCACATGTCCTACTCCTACTGCCACGACCGGCGCCGGTACCCTGTCGCCCTGTCCGAGTGCGACGCCGCAGGGCTCCGCCGCCTGTTCGGCCCGGACGGGATGAAGTTCGGCCGCCGTGGAGCGCGCGGCCGCCGCTCCGACGTCGTCATGTGATCCCGCGCGGCCGGCGCGCCCCGCCCACCCGTCGCGGCATGGTGTGGCATTGCCATGGCGCGGGCGTTCCGGCACCGAGGGTTCCAGGCTGTATAGAGAGGGCTGGGTGGGGCGGCGTCCTTGCTTGCTAGGGTGACTGACTGCCGCGCGCGGTGGAGACCGGTCGGAATTTATGCCCCCCGTTGTTCGTCACGAAGGTTTCCCGCGGCATTGTACTCTCGCCCTGCCCCATCCGCCATGGATCCGGGGCGGGCGAGTAGCTTAGCGTTTATTGCCTCCTCTGGACCATGCATCCGTGCCATTGATGATCGACTCAACAACGTTAATTCCGAG

**List of sequences of identified genes in Table S12.**

1. Gene ID: DN73397_c1_g4

GGCAGCATTACATAGTGATGATCGGGAAGGGAAATGGCAGCATTTGCATGTCTCTCGCTCACTCGTCACGCATTTACACTGAAAATCCACAATAAACTTAACAAACACAGTAGTGCTAGATTCGCGTACGTACATATACCAAACTCCCAAGCTACGTAAATGGCAACCTCAGTAGCTGGTTGCCGTTATTCTTGTCCCCATTTCCACTTCTCTGATGAAGTACAAATTAAAGATAGCGCGAAATCTTATGCCTGCATCGATGGTGGCTGCCATTCGGTGCTTGAATGAGGGTACTTGTCATCCTTGCTGACGTCCGCGAGGCCTATGCCGGCGGTGGTGGAGGTGGTGCCGGGGCCGACGCCGTTGTTGCCGTCCTCGCCGGAGATCTCTTCGAGCGACCGGCCCATGGTCTCCGGGACGAGGAAGGTGAAGAAGAAGCCGAGCATGTTGGTGACGGAGAGGATGATGAGCGCCTCTGCCATGTGCTTGGCGTCGCCCTTGAGAGTGAGCCTCTGCACCCCGAAGGCGGCGACGATGGCGCCGGCCTTGCCCGACGCGGCGCTGATGGCGTGGCATGTGGAGCGCACGCGCGTGGGGAACAGCTCGGCCGGCAGCACGAAGGTGGTGCTGTTGGGCCCGAAGTTCGCAAAGAAGAAAGTGAGCGCGTACAGGATGGCGAAGAGGATGTGGTTGCTCTCCTTGAGGTATTCGTACTTGATGCCCATCACCAGCATGAACAGGGACATCATGAAGAAGCCAAGGAGCTGGATCAGGTACCTTCCCATCTTGTCGATGAGTGCTACGGTAACCCAGTAGCCGGGGAAAGTGCCGAAGAGGGCGATGAGGAACATGGCCCGTGATAGGACGAACACCTCTCTCAGGGCGTTCATGGACTCTGGGGGGCCGGTGAGCTTGATGGCTGGGAAAATGTCTTTCTGCGTCAGGTTCTGGCTGTAGAAGGCTACGTCGAGGAGGAACCACGTGGTGGTGGTGCCGAGGAGGTGCAGTCCGTGGCGTTTCGCGAACTCCTTGGACAGCAACGAGTACTCGTTGGCCGCCCTGAACTTGGCGAGCTTCTCCTGCTCGTCGTCGATCGTGATCTCCAGCACCTTCTGCATGTCGTTGGAGGCCTGCTTCGCGTTACCCTCGATGATCGCCGTGTACCTCGCTGTTTCCGGCATCTTCATCCTCCAGTAGAAGGTCGCCAGCGCCGGTAACGCACCGAGCATGAGCACGATGCGCCACATGTAGTCCGCCGCCGGCAGCTGGGCCGACAGCCCAGGGTTCTCCTTGTACGACGGCGCAGGGTGGTAGTGGAGGAGAATGCCTGAGACGATCATGGAGACGAGCCCCGCGAAGATGATGCCCACACCCTGCATCGCGAACACGGCGGCAATGAACGCGCCGCGGGTCTTCTTGTTGGCGTACTCGGACATGATGGTTGCCGACAGCGGGTAGTCCCCGCCGATTCCGAAGCCG

1. Gene ID: DN87006_c2_g1

GATGAAGATGAACCGCTCCTGGATGAAGGCCGGCACGGCGTCGTTGGAGGAGAAGAAGAAGACGCAGACGGTGAAGATGAAGAAGCTGAGGCGGTTGGTGATGCCCTGCGTGTTGTCCTTGGGCTTGGTGAACATGGTGGCCATCAGGAACCCCATGACCGTCAGCACCATCAGCCGCGACAGGAACAGCTCCGGCGTGCGCCAGATGTTGGTGAAGTTGCGCCGCATCAGCACCCACACCTCCCCCGCGTACGAGTTGGCGAACTTGCCGCGGTGCCCCAGCGTGTTGACGCCCACGCCGGGGGCGCCCGTGGCGGTCTTGCGCTGCGCCGGCGACAGGTAGTCGGCCTCGTTCACCGTGTAGACCGAGATGCTGCTCAGCGGCGTCGGCGTCCCCATCACGATCTCCGGCCCGTACCTGTTTTGATCTTTCTTGGGGCGGCGGGACGGCGTGAACGGCGTGCCGCTCCAAGGAGACTTGGAGTGCTGGCTCCTGAGGCTATGGTCGAAGTCCTCGCCCCCGGGCCCGATCGGTGTCGCAGGGATACTAGACACGAGGGAGAGCCCCTCGGCGGCGAGCTTGCGTGGCTTGAGGCCGGTGAGGCAGAACTCGGCGAGGGCCTTGACGCCGAACTCGGACTGGTCGTACTCCTGGATGACGTCGAGGAGGTTCTCGATGGAGTTCTCCCCTTTGGGCACCTTGCGGCCCATGCGGCCGAGATGCGCGGTGACATCCTTGGGCCCGCCGCTGTACATGAGCTGCCCGCGTGCCAGGATGATGAGGTGGTCGAGCAGCTGCAGGATCCGGGACGACGGCTGGTGGATGGTCAGCACCACGGTGCTCCCGGCGCACGCGATGTCGTGCACCTTCTCGATCACGCTGTGCGCGCTCGTCGAGTCAAGCCCCGACGTCGGCTCGTCCAGGAACAGCAGCGCCGGCCCGTGTATGATGTCCACGCCGATCGAGACTCGCCGGCGCTCACCGCCGGACACACCCCTCGTGCCCTCGTCCCCGATGTACGTGTTTCTTGATGTCGTGAGGCCGAGCTGCTCGATGAGGTTGTCGACGCGGAGCTTCTTGTCGGCGGCGGAGACGGTGGAGCCGAGGCGGAAATCGGCGGCGAACATGAGCGTCTCGTACACGGTGAGCATCGGGAAGAGGCGGTCGTCCTGCATGACGTAGGCGGACGAATGCTTGATGACGCTGGGGCTCATCTCCACGCCGTCGAGCGCCACGCGGCCGTCGAGGCTGGATATGCGCCCGGCGAGCGCGTCCAGGAACGTCGACTTGCCGGCGCCGCTGGGACCCATCACGGCGGTGACGCAGCCCTTGGGGGCATACCCCGTGATCTCGTGCAGCAGGTCCACGTCCTTCTTCTCCCACTCGCCGCCGACCCCGCGCTGCTTCTTCACCACAGTGTACGTCAGGCTGGTGAACTCCAGCCCGCCGGTGAAGCCGAACCGGCTGCCGCTTTTCGCCGTTGCTTTCTCATTCTGGGCCATGTCCATCGCCGCCTCCAGGTCTCGGTGGCCGCCGCCGTGCCGGTAGTTCGGCTGCGCCATGCTGCTCTAGCTAGCTACTGTCTATGTGCCAGTCGAGTATGTTGTTT

1. Gene ID: DN74986_c1_g1

GCAGCAAAAGATCTATGCACGCATCCCGCCCACACCTCTGGACTCTCTACCTCCACCACACTCGGACACCAACAGTCTAGTAGCCGGGAGCGTTGAGGGTGATGCCCATAAGAATACCACCGATGAGACCGGCGATGGGGATGGTCATGACCCAGGAGAAGAACAGGAGGCCGACACGCTGCCAGTTGACGGCTTTGAAGGTGCCGTTGCAGAGGCCCACGCCGACCGTCGCGCCGGTGATGCACATCGAGGTCGACACGGGCAGCTTGTACTGCGAGAAGATCAGGATGGTGATGGCCGCGCCCATTTCCATGGACGAGCCGCGGGAGGGGGAGTGGTAGGTGATCTTGTTGCCCATGACTCGCATGATGTTGTAACCGTAAGTGATGAAACCGAAGCAAATCGTCAGAGCGATGACGGCGATCTGCCACAGAGGGACAGGGGCCTTGGAACCGACAGTCTCGCCAGTCGACCAAGCCGAGTACATGGCAGCCCAGACACCGACGGCGTTTCCGACGTCATTAGCACCGTGCGCGAAAGAAGCAGTGCAGGCCGTGATGATCTGCACGAAGGAGTAGAGGTATTCGACTTCGTTGCTGTACTTGGGCGCGTGCGAGTACACGCGGTCCATGCGGCGGCCTTCAGGAGTTCCGAGAACGCCGACCTGCGCGCGGTGGATATCGTACGACATGCCGTAGAGGGCGCCGACGACGATCTGAGCTGGGATGCGGACGAGGGTGGCCTTGAGGTTGTGGAGCTCGTAGATGGAACCGGCACCGAGCTGGTTGTTGTGCAGAGTGCGCATGGCCCAGCCGAGAGGTCCCTGCTTCTGCCTGAGTTCCTCGTGGTGCTGCTCGCGGGCCTGAGCCAACAAGAGACGGTAGGCCTCGCGCGGGTTTTCGTGGAGGGGAGCGGGCCTCTCCTCGGAAGAAGTGTCGACGTCGACGACGGTGATCTCCTTGGTGTCGGCGTCGGACATAGCAGCCTCGGCCTTCGCGCCCGGGCGCTTGATGTCACCGCTGTGCTGGACGACGGCGTAGTTGGGGACCTGAGCGAACTCGGCATCGGCGGGGGCAACCCTCTTGAAGAGAAGAGGACCCTGGAAGACATCCCAGAGCTTGAGGGTGTAGTCCTTCTTGATCACCTTGGCGTGAACGAAGGGAACGAAGAAGAAGGCGGACAGGACGAAGAGACCGAAGCCCACGCCGAGAGTGACCGAAGCAATGTACCAAGCAGGCTTGTCAGACAGGCCAAGCCTTGGCGAACCCTTGTAAACGACGGACAGGGTGCAGATGGTGCCAGCGATGAGGAAGAAGAAGGGCGAAGTCCAAACGGCCCAGGGAACAGGGTCGCGACGGACGTGGACAACGAACTTGATCAAGGAGAAGATCACCGAACCGAAGATACCGGACGCGATCGGGGCCATGCAGAGACCGGCAAAGATGGCTCCAAGGCCGGAGCCACCGTTCCATCCCCACTCGACCTTGCTGGCGCCCACGGCAGCGACACCGACACCGGCGATGGAAGAAACCAGAGAGTAGGTCGAAGAAACGTGGGCGGAGTTGCGTGTACACCACATGACCCAGATGGCAGCACCGGCGGACGCGCAAGTGAATGCGAGGAGCTGGACACCGGCATTGCCGCTGAAGGCGGCGATGGGAATAATGCCGTTCTTGATGGTGGAGGCAGTCCTGGCACCGACAGTGAGGGCACCGGTCAACTCGAAGATCAGGCAGAGAACCATGGCCTGCTGGTAGGTGACGGAGCGAGAGGAAACACTGGTGGCCCAGGCGTTGGCAACATCGTTGGCACCATTGTTGTAGGCCTCAAGCAGGGCAAAGAAGGTGCCGATGGCGAAGACGTAGGTGTATTCGGTGAGCATCTTGGCTGCTGCGGTACAGGCGGTGCACTGCGAGAGCTGGTGGGCCGATGGCTGTGG

1. Gene ID: DN40831_c0_g1

ACCTCACGTTTCTTCACGAGCTTCAAGATCCAGTGACGATGCCTTCTCTGCGCCTGCTGAGTCTTGCGGCCTTCGCCGCCTGCGCGGCGTCTCTTTCGACGGCTGAACGTGAGACTGCAGACTCTACTGCGCAGAAACGCCAGGAGTTCTTCGAGGTGGGCAGTTGCCACGCTCACGGCGATGTGCTCTTCTGCATCCACGATGGCGCGGAGTGGGAGGTCACCAGCGACGTGACTCCTGCAGATGCGCCAGACAGTTTCACCGGATGCCACTCCCATTCAGACACAGAGATCCACTGTGTTGATGCCGACCAGACCTACGACGTGAGCTTGATGCTAGAGGGCGCGGAGTCCACGAGCACCGAGGAGGCTGGGTCGGAAGATCACGCCCATGCCTCAGACGCCACGGCAGCCGAGGGAAGCACCTCGTCTGAAGAGATCACCGAAGTGACCTCTTGCCATGCCCACGCTGACGGCCTTTATTGCATGGCCGATGGCGAAGAATGGCAGGTAACGACTGACTACGACACTGCCAACCCTCCGGAAAGCTATACCGGCTGCCATGCGCACGGTGCAGACGAGCTACACTGCACCTCAGGTAACACCGAGGCCAGCTTGTCAAGAGCTGGCGCCGAATCCCACAGTGGATCCTCCGAATCATCATCATCGGCTGCTGGTTCTTCCACCGGCGAGGCAGTGGACTGTCATTTCCACGCTGGTGTTGAGCACTGTGTTGACGAAAATGGCCGAACTGTCGAAATGGGCTGCTCAGCACCTGACCACGACTACGATGTCCCTATTCGCATCGGCCTCCTTTTCGTGATTCTGGTCACCAGTGCCATTGGCGTGTACATACCCATCTTCACACAGCGATTTACGTCTGTCAGCATGGACAGTACGCTCATTGTTGCTATGCGTCAATTTGGTACTGGTGTTATCATCTCGACCGCCATTGTACACCTTTTCACCCACGCACAGCTGTTCTTCAACAACGACTGCCTCGAAGGCGTCGAATACGAGGCAACTACTGGTGCTATCCTGATGGCTGGCTTGTTCTTGACTTTCCTCATTGAGTACGTTGCACACAGATGGGTTGACCGGAAACGCCACATGTTCGAAAGGCCAAACGCGAATGCTGTCGAATCAAACGGGAATAAGGATGGTTCGGACGCCAACATCTCAGAGGCCAGCTCCGGTGAGCAAAAGTATTCGCCAGCTTCGCTTACGCTAAACACTGCCGTCATGGAGGCTGGAATCATTTTCCACTCTATCCTCATTGGTCTGACCCTGGTCGTCGCTGCCGACTCTGGCTTAATCACCCTCTTCATCGTCATCCTGTTCCACCAGATCTTTGAAGGTTTTGCGTTGGGAGCCAGGATTGCCATGATCCCTTGCGGACTCGTGCGCAAATTGATACTTGGAGCAGCTTTCGCCATCACCACCCCAATCGGCATGGCAATCGGAATCGGCGTTCTCTCGAGCTTCAACGGCAACGATCCTTCGACTCTCGTCGCAATTGGCACGCTCAACGCCTTCAGCGCTGGTATCTTACTCTGGGTCGGTGTGGCTGAGATGTGGTTTGTCGAATGGTTCCACGGACCGCTGACTCATGCCGGACCATTCAAGGTGGCGGTTTGCTTCTTGAGCTTGGTGAGTGG

1. Gene ID: DN55460_c0_g1

GGTGTAGTGCGACGGCGACGAGTTGGCAGACGACGCAGCGTCGCTGCGCGCATCGTTGACGCGCTTGAGGAGCTGCGCCAGGGTGGAGGAGGAGTGCTTCGTGGGAGGCGACGGCTCCGGCGCCATCATGGCGCCCTGCTGCGACTGCTGCTGCTGGTGATTGTAGTGATGGTGGCCGCCGTGCAAGTGGAGGAGGCCGCTGCGCGGGATATCGTCGGCTTCCTCCATGGGCGATGCCCTCCGGTCAAAGAACGGCAGCTTGTCGACAAACCGCGACATCTTGCTCAGCTCAGCTCCTCCCCGTGTACCTATGCAAGCTTGTAGCTGCTTGCACGGTCGGCCACCTTGGCGTGGCAAGAGAGATGGATGGAAGTGGCG

1. Gene ID: DN66524_c2_g4

GTCACGGTAAGGTTCTTTTTATTTTATTTAATCTTGTACCAATGAAATCATCTGAACAACATGGTCTAATTTACATAGACTATGAGAGTATATTACACAAAGTTCTAGCTTGCAACTACATCTTGAAGGAATGTAGTAACCGTTGTTGTTACATACTTCTCATGTATGGTACTCATATGCATTTGGCAAGCTAAAACATCCTTGCATATTTGCCCATCCACCATTATTCTCGGGTCCATGAGCTACTTGAAATCATATTTCTTTGGTAATGATAGGAATGGCTTGAAGGACAACGCAAGCCCAAGTAGTTCAACTAAATTCAGTATCACAAATATGATTTGATCCCCTGGAAAGAACGTAGCATTTTGGCGCTTTTGGGCCCATGAGAACAGAACACCTGCTCCTACTGGAGCAATAGTCTTGAAGAGGGACTGTCCCGTCGCGGATAATCCATTTGCAGCACCTCTTTGATTTTGTGACACTGCATTATTTTGTAGAATAGCATTCCCCGTTAAGATAGTCATAGCAAGAGCACCCTTTGCAATGGCTGCAAAATATATAGCTAGCCCAAGTTTAGTCCCTGATAGGTTTGTCATGAACGGGTAGCAAGCAAGAATTGGTATGGATAGAGCGGATGCAATACGCAATGAATTGACAGATCCCAATAAATTATGAACATGTCGAAAAATGAATAGTTGATACACAAGAAGACCGGCACCTGAAACCGCGAGAACTTGGCCAACGTCTTTAGATGAAAATCCTAGCCCACCATACTTTTTGTCACTTACTGTCCACAATGAAAATATCTCACTGTATGCAGTATCATGCAGGGTGAAAACACAATATGCTGCTATAGAAGACATCAATGGCCGATTCTTATATAGACTCTTCTCAGGATGTGGAGCTTCCCTACGAGGTGCCATTCTTGTATCACCAACCATTTCTACTTCCACTTCTAAGTTTTTATGCATATGCAGTGTCTCCGGTAGCCATATGCAGCTTATTAGAGCAGCGAGCGCAACCAATGAAATACAAAGGCACGGCAAGAAATATGGAAACCTCCCAAAAATTGAATTCTCGGAAAATAGTTTTGGGTAATGCTTTGCAGGCTGTGCAAGGTAGCCTCCAATGGATGGACCGATTACAAGACCTATGCCCCAGGCACTGCTTAGAACTGATAATCCCAGCGCATGATGTTCAGGTCGACAAACTTCGACACAGTAGGCCTTCATTGGGGCAAGCATGCCATTTAGAGAACCAAGAAGTAATCTTGTGGTAAGCGCCATCCAATAATTCACACTTAGTCCAAACAGAGTGTTAAGGATGACCACTGTGAAGAGGGAAAATGCAATGATAGGTTTTCTGCCCACTCGATCTGCCACCACACCCCAAAACAGGGACCCAGCAAATCTACCGGCCATGTATGATGCCCCAAGAAGACCAGCATACACTCCAATGTCTTGTTCGTTTTTCGCCACATGCATATCTTGTATCATGAAATACAAGAAGGGGAACAGCGACGATATTGGTAAAGCGGAGGCAAGGCTGGTGACTCCGACGAAGACGAACTCCTTGTAAGGGATCCCCGTGCTGTTCTCTACCTTGCGTTCCATGGCGCAGCCGGGGCAACCCTCGTAGTACACCGGCGCCGCGTCCTCTGGCGGCGGTGGAGTCGCATTCGCCTCATCGTTGTCTGGGAACGTCCGACTCCTGGTAGTGTTGTCCATTTGGTTCCTGACTAGTTAATCAAGCACTAAGCTAGTAGCTAGCTG

1. Gene ID: DN76268_c0_g2

ACCTCATCCGGGGAACTTAACCATGGATTGAATTCCACACGAGCACACATTTACACAACATATTCAAACATGTACGAGAAGTCATGCAAATGACATAAATACATATGTGCACACTGTCAAACATAAAACCGAAATGAAATTAAGCATTGTTTGCATGCCCACGGGCATAGATGCTGAAGAATCAAATCAAATCAAAACACAACATGCTCATGAATTAACCGATCCATGGATCAGTCCCATATCATGACAACGGCCATGACAGCAACGCCAGCAAGTGCGCCAAAGAACTTGAAGATGGGCTTGTCAAAATAGTTTGGTTGCTGTGGACGGTACCCCTTGGCCATGAGGTGGTTAACAGCGACATAGATGAAAACCCCCGTGGCGATACCCATGGATATCGCGTAGGTCCAGTCAGCTGCTGAACCCTCAGCTGTGGCGTCGATGGCGATACCGATTCCCACGCCTAGTGGGCTAGACACAGCGAAAGCCAATGAGTAGACAACCGTCATTAGGAACGGGCGCTTGGGGATCATCCGGAGGAGCGCGATGCCCATGGCCACCGCCGCGAAGATCTTGTGGAGCCCGATCGTCCATAGGTTCCTCCACGCCTCACCCTTTGTCGCTGAGACGCCTATGGCTATCCCCTCGAAGATGGAGTGGAAGCAGAGCGCAACGATGAGGAGGATCGCGTCCTCGAATGACGACGCCGCGGTGGCCATGAGCATCGGGTGCTTCCCGTGCGCGGCTGATGACTCCACCCCGTCAACCTCGCCGCCCTCCTCCTTGACCTGGTTCTTCTGGCGCCTGGCGACGGCGACGATGGCGACGTCGCTGAGCATGGTGAGCAGGAACCCAGCGCAGGCGAGCATGAAGGAGAAGGGGTACTTGTTCTTGGTGAGGCCATGGAAGGTCTCGGTAGCGTCGGCGAGGAAGTGCATGAGCGCGGTGCCGAGGAAGATGCCCGCAGCGAATTGGGTGCCGAGGAGGAGGAAGGCCTCGTTCCAGCGGTAGAAGTAGGGCGACACGCCGCCGAGGAAGGTGCCCACCAGCAGGATGACCAAGCACCACACCTTGACGGCTATGAGGCCCTTGGAGCGGAGCACCGACCTGTCAACCGCCGGCGCGCTGTCGCCACCCGCTTCCCCGTCGCCGTCGTCGATGCCGCCGTGGCCGCCAGCCGGGTGGAGCTGGATGCTGAGCAGCGCGAGGAAGAGGAGCACAGTGCACATGAGATTCATCTTCGTCTTCATCCTGCCAGCCATCACCATGGAGAGTTCTGTCGCTGGCTAATCAACCTAGCTAGGTTTGTATCCGTAGCAACTTCGACGATCTCTGCACACATATATATGGATTGACCAACCAAGGTACAAAT

1. Gene ID: DN76268_c0_g1

CATGAAGAAAGACAAAATAAAAGATGAATAAAGACAGAATACATATGTTACTTCGGACGTACACTATATATCTGAATTCTTTGGTCAGAAATTTGAATTGAGCTGGACCTACCCTTGGATACATATTTGTTTGATAATTCTAGGCTCTCGATGAACCACATCCATCATCTTTCCAAAAGTTACTCCATACAAAGACGCAGCAAATTGACAATGCTGCAAATAATTATTCAGCATCTCATTTGAGGAGCTCAACAGTGGATTAGCATCCACATGACTACACATTCACACAATCAATCCGAACATGTACCATATGTAATTTAAATACGTATCTGCACATTGCCACACACCAAGGCCAATATGAAATCACATCATTGTTTGCATGCCCGCATGCCGAAAAATCAAAACCAAAAGGGTCGAGAATTAACCGAGGGATGATCAGTCCCATATCATGACAACAGCCATGACGGCTACGCCAGAGAGCGCGCCAAGGAACTTGAAGATGGGCTTGTCAAAGTAGTTTGGGTGCTGCGGATGGTACCCCTTGGCCATGAGGTGATTGATAGCAACGTAAACAAAGACCCCTGTGGCGATCCCCATGGATATGGCGTATGTCCAGTCGGCTGCCAAGCCCTCGGCCGTGGCGTCGATGGCGATGCCGATGCCGACACCCAGCGGGCTAGACACAGCGAAAGCCAACGAGTAGACAACAGTCATGAGGAAAGGACGCTTGGGGATCATTCGCAGGAGCGCGATGCCCATGGCCACGGCCGCAAAGATCTTGTGGAGCCCGATCGTCCACAAGTTCCTCCACGCCTCATCCTTCGTTGCTGTATATTAATTGATCGATTTAATTGCAAGGTATACTTTTCTCTATAAAAAAAGTAAAAAATGGTTTGGTGTCCTTCATAGAACGGTTATTCATCTTTTAACGGTCTATTAGATATGTGAAATTTTAGGTTTTAGCTAGAGATGTGCAGTTGCAACCTAACAGCTACATGTATATTGATCTAATTGTACTTGTAAAATACATTTCGACAAAAGGAAGAAAAAATAGTACATGTGTAGCACGGTATACTAGCAAATTAAGTAAAAAGCACTTGCGCATTGGCTTAGCTAGGAAATGAACGGTGGTGTGTTGATCAAGAGTGTGCAATTGCAAGTCAGCAGTTGCATATTTGTTGATGTAATTAAAATCATCAATTTAGTGTCAACAATATGTAAGTATAACACAGTGGATTAAAGGAAAATACTACTATATATAGTAGAAGCCAGGCGTGCATTGGCGTAGCTAGCAGATTAACGATTGAACTGGAATGGTGGTGTACGTGTGGAGTTGACTGCTGACCTGAGACGCCTATGGCGATCCCCTCGAAGATGGAGTGGAAGCATAGCGCGACGATGAGGAGGATCGCGTCCTCGAATGACGACGTGGCCGTCATGAGCATCGGGTGCTGCTGTCCATGCGCCGTCGTCGCCACCGAGGACGACGCGTCCACGCCCTCCTCCTTGCGGCCCTCTTGGTCGACCTGGTTCTTCCTCTGGCCGCGCCTGGCCACGGCGACGATGGCAACGTCGCTGAGCATGGTGAGGAGGAACCCGACGCAGGCAAGCATGAAGGAGAATGGGTATGGGTTCTTGGTGAGGCCATGGAAGGTCGCGGTAGAACCGGCGAGGAAGTGCATGAGCGCGGTGCCAAGGAAGATGCCGGCGGCGAACTGGGTGCCGAGGAGGAGGAAGGCCTCGTTCCAGCGGTAGAAGTAGGGGGACACGCCGCCCAGAAAGGTGCCCACCAGCAGGATTACCAGGCACCACACCTTCACGGCGATGAGGCCCTTGGACCGGAGCACCGACCGGTCCACCGCCGGAGCGCCCTC

1. Gene ID: DN81633_c1_g1

AAAATTTAAAAAAAACTAATAAACTATTTCTTTATTTGTTTAAAAAAAAGTTTTTTTAAATTTAAAAGCAAATCGTAATTCAAACTCTCAAAAAAGAAAAAAAAGATTAGAAAATTAGATTTTGAATTTAATTTTTTAGAAAGAAAGAAATGGAAAGCTTAAGAACTTCTTTGTTGAACGCTTCAACCACTGAAGCCCGTACTACAGCTGCATCTGAGTTTGCTAAAGCAGTTGAGTTGAATAATGTTGAGTCAAGTGGTGTTTTGAAGACTTTGGATGAGTTATTAAGTGGTGCTCCTGTTGCTAAGGAAGGTGGAAGTCTTTTATTTAAGGCTTTGGTTGATCGTTTTCAACAATCTGCTGTTCCTTTTTTGTATCCAAAGTTGTTACAAGTCATTGAACTTTCTGGTGATAAGAAGTCTGCTGAGTTGAGAGATGCTGCTGAAGCTGCTGCCAAAGCTTTTGCTAAGAACATGACTGCTTTTGCTGTTCCTCTGGTCTTTGACCAAATCTTGGAAACTTCTGAAAAGTCTACCAAATGGCAAGTTAAGGTTCTTTGCTTGAAACTTATGTCAAGCTTTGTTAACACTGCTCCTGAACAAGTTGCTCAACGTCTTGTTGTTATTGTCCCGACTGTTTCTGCGATGATGTGGGATTCGAAGAAGCAAGTTCAAAAACAAGCTGCTAAGGCTTTGAAGAAACTCTGTGGTGCTATTGACAACAAGGATGTTGAGCCTTTCATACCGAAATTAATTAGTGCTATTGCCAAACCAGAGGAAGTAGAAGAGTGTGTTCACGAACTTGCTGCTACGACCTTTGTGCAGACTGTTGATGCTGCTGCATTGTCTATCACTGTTCCAGTGTTGGATCGTGGTTTCCGTGAGAGAAAGGTTGCTGTGAAGAGAAAGTGTGCTGTCATTACGGAGAATATGGCTAAACTCGTGGAAAATCCTGCTGATGTCGCACCGTTCATGCCTGTTCTTGAACCTCACTTGGCTCGTGCTAAAGAAGAGGTTGCGGATCCAGAGTGCCGTACTCGTTGTGCTGCCGCTCATCAAGAACTGTTGAATATCAGCAATCGTGCTGAGAAAGTTGCAGTTGAGAAAGCTGATTACGAAAAAGTTAAGGCAGATGTTAAGTCTATTGTTGGAAAGACTGGACCAGTCGACGCTGTTGACCCTGAAGGTCTTGTGGTTGATTACTTGGCCAAACTTATTCAAGGTCTTTCTGTTTCTGAAGACGCAGATGAGGAGGACTGGAAAGAAGAGCTATTGCCAGGTTTGAAAGTCTTTGGCATGGATGAAAGTGCAGCAATTGCTATAAGACAAAAATGCGTAGGTGGCGCCGATAACAAAGAGACTTTTGAAGATGATGCTGAGGAAGATGCTGCTGAAAAATTGTGTGATTGCCGCTTCTCTTTGGCTTATGGTTCCCGTATTCTGTTAAACAATGCTCGTCTTTACTTGAAGAGAGGTATGAGATATGGTATTGTAGCAGCAAAGAGCGCTGGTAAAACCACGCTTCTACGCTCCATCTCAAACGGCCAGTTAGAAGGGTTTCCTGTTAATACCTTGAAGACTGTTTTTGTTGAACACGATATTCAAGCGTCTCAGGCTAGAATGAACACTATTGAATTTACTTTGGACACTGTGAATGATGAAGGGGTATCAAAAGATGATGTGATCAAGATGTTGACTGAAGTTGGTTTCACTGATCAAATGCAAAATTCCCCTATTACCTCGTTATCTGGTGGATGGAAAATGAAATTGGCTCTGGCTAGAGCAATGCTTAGAAAGGCTGATATTTTGTTGTTGGATGAACCAACCAACCATTTGGATGTTGTGAACGTGCAATGGGTGGTAGACTATCTTACCGGTCCAACTTGCAGCCATGTTACTTCGATGATCGTTTCTCACGACACTAGTTTCCTTGATAAAGTTGCAACACATATTATTCATTTTGATGCTCTGAAGTTGAATAACTATACCGGCAACTTGTCCAAGTTTGTTGAAAGGTTTCCTCATGCTAAGGCATATTATGACTTGAGTGCTTCCTCACTGAAATTTAAGTTCCCGGCTCCAGGGCCTTTGGATGGTGTAAAATCAAAGGGACGTGCAATTTTATCTATGACCGATGTTGAGTTCAGATGGCCTGGACAACCTGTACCACAACTGAAGAATATCAATATCAAGGTGTCCATGGCTTCTCGTGTTGCGTGTGTTGGTGCGAATGGCGCTGGAAAGTCTACTATGATTAAACTGTTAACTGGTGAAGTCAAGCCGGATCATGGTACCGTGTATAAGCATCCAAATTGCAATTTCGCATACGTAGCACAACATGCATTCCATCATATTGAACAACATTTGAATAAGACTCCAACTGAATATATTATGTGGCGTTACAGTGGTGGTGAAGACAAGGAAGCCTTACAGAAATCTACTGTTAAGATTACTGATGAAGAGCTGAAGAAGATGAAAACTCCAATTTTGGTTTCTTATGAAGATGAAGAGGGCAAAAAACTCAAAGAAAAGCGTGTTGTTGAGAAGATTCTGACTCGTCGTCGTCAAGGAAAGATTTATGAATACGAGGTGAAATGGGAGAACAAGAGCCAAGATTTGAATACTTGGTATCCTCGTCATGAGCTAGAAGAAATGGGATTCACGAAGTTATTGGATGAAGTTGATCGTCGTAAGGCAGCTCAAGAAAGTGCGTTTGCGCGTGTGCTTTCAAAGGCAAACGTTGAACAGCACTTTGAAAACGTTGGTCTGGACCGCGAATTGGCATCTCACAACAGAATTTCTTCACTTTCAGGAGGCCAGAAGGTTAAGGTTGTGCTTGGAGCGTGCACATGGTCCCAACCTCATTTGATCATTTTGGATGAACCCACTAACTACTTAGATCGTGAAGCTTTAGGAGCTCTCGCAAGCGCTATTAATGAATTTGAAGGTGGAATTGTTTTGATCACTCATAATCAAGAATTCGCAAATGCAACCACTCGTGAAACATGGGTTGTTGCAAACAATACTTGTGATATTAAAGGTGACGCAGATTGGACAGCTTATGCTGCTGAAGCAATTGAACTTGATCTCAATTATAGAGAAGACCGTCATGACGCTTTGGGTAACAAGATAGAAGTGAAACAAACACCTGCATCTGTGAAACCAAAAGATAAGAAGCGAATGATGAAAGAATTGAAGAAAAGATTAAAAGAAGACCCAGACTGTCGCTTGAGTGAATTTGAAGAAGCTTGCTGTAACGAATGGGGACTTTTTGAATAGAAAGTTAGTT

1. Gene ID: DN79236_c0_g1

CGCATTGCTAATTCGCCATACATTTGTGTTCAAAAAAACATTCGTCATACATATATCTGAAGCCGCGACTATGATGAGCTGCAAACAGAGGATGCCTCGCTCTCCCTGGCAACTCTGATGAACACGTCCTCCAGAGTAGTGTCAGCCAGGCCCCAGGCGAGGATCGTCACCCTACTCTTCGCATTCTCCATGGCATGGAACACGTCTGATATCCTGACCTCCTGCTTCGGCATCTCAAACTTCTGCGTCCCAGAGATATGGTACACCCTGCTCACCCCAGGCGAGATGGACTGAACTAGTTTCTCCACCTCCTCCGCCTCCTCACCTGCCGCAGTCGTTATTGTCAGTACATATGAGCCTCCGTACTTGGTTTTCAGCTCTTTTGAGTTTCCGATGCACTGCAAGGTACCGTTCGCGATTATTCCTATTCGATCGCACAGAACTTCGGCTTCTTCCATTGAATGTGCTGCAATATTGATGGAAGTAAGTTTGAACTTTGATGATGCAGTATCAAGCTCAAGTTGCACAGACACACACAATAATGTGGTACTGAACTGAAAATACACTGCTGTTTGAGAATTGAAGGTAAAGTACTGTACTCGTGAGAATTATGGCCCTGTCCTGTTTAGCAGACTTTACGGCATTCCATAAGTCTTTCCTCGATGCGGGATCTAAGCCCGAACTTGGTTCATCCATATAAACAACCTTTGGGTCACCAATTAGAGAGATTGCAACACTGAGACGACGCTTCATGCCACCGCTGTATTTTGCTACAAGCTTATCAGCAACACCACCCTCAAACAAACGCACACTTTTCAAAGATTGTTTGATAGCCTGAGCTAATTGTGTACCCTTCAATTTCTTAAGCCTTCCGTAGAACAACAAATGCTCTCGGCCAGTCAGTGTTTCCCAAAGCAAGTCATGCTGCGGACAAACACCGATTCCTGTATAAATTTTGTCCATGTCCAGTCGTATGTCCATTCCTTCTATGTACGCCGTGCCAGACGTAGGTTTCGTAAATCCAGTGAGCATGTTGATGAGTGTGGTTTTTCCGGCACCATTTGGACCAAGAACACCAAAGCACTGCCCCCGAGCCATTGAAAGGGACAACTCTCTGACAGCAATTTTCTTTGAGTTGCCATCTTTTCCACGATACACTTTCTTAAGGTTGTCACATATGACTGAATAACTTCTATTTGGTTCCTGTAATAGCTGTCCAACTATTTCTCTCTCTTTGATAACATCTGTCCTCTCCATTTCAACAGAAGCTTTGAACTCTTGAACTTGCATGGTCTGCTGTTGAGCTGCTGAAGATTGCTTTGCAGCCCGGTGTGAGTGAAGAAGTAATACTGCTTTTCTTATTCCATTTTGAAAGGAACCAAAGTGATCCAGGTAGAATGATAATGACATGAATAGGATCCATTCGAATATCATTATGGTTAAAACACTTCTCATCCCATTTTTGGTATCACTCAGGTCGCTCCACTGCATACCCGTGGAGCCCATAACACTTACCAGTAATGCAGATTGTGATAACTCGTACACAATGCGATACAAAGAAAATGGAGGAAAAAGTTCCAAAAGTATAATCCAGCTTCCTGAGAGGAAAATGTCTTCAATATATGGCTTGAAGAAAAATTCTGCTATAAGTCCGGACCCAAATATGTAGAAATATCCTGTGACAGTAGCTGTCCTCACATTTGAGAAGCATGTAGCCACAAGAAATGCAAATGAAATTTGCAAGTTCATGTAAGCAAAGTAGAACACAAACTGCAGGTCATAATTATTTAGTCGAAAGAATGATATTCTTAGTGCAACACCAAATATCATGAAGGACAGCATGTATAGCGTTGATAGAAGGATAAAATACGAATATGTTATAGTCCAATATGGCAAATCACCAAGACCATGCATCTTCATCATGATCCTAAGCTTATTTTGCTTCTCGTATACAAGGTTGGTCAAAATTACCGGGAAAAGAAGCATCATAGTCCATAGATAAGGCAGCTGTCCTATTATTGAAGATATATCAAAGCTAAATGATTTAGCTGCTCTGGGCATATCTTTAACAAAATCAAATGATATCTTGAGAGCATTTCCTCTTAAATGAAGGTATGCATTTGATGCCATATTTGTTAACCTTGGAACTTGAAGCAATCTAGGTGCTTCCCATGGCCCAGGTAATTGGATGATAGAGATTCCTGTGTCCACATCACCAAACTTGTTTGTCGAGTTATATGAAATGACCAAGTTAAAGCTGCTCAGATCAGAGCTTAAGAAGTCGTACGCTGCAGCAATTTCATTTGTCTTGTTCCCTCCGTAGTATGCTTTATACAGTTCATCACTAATGAGCCATGAATTGTCGCGCCAGAGCATTAATCCTTCCGTGCAATTCACATCTTGGCTGACAGTTTCATTGCCTTCTATATAAGCATATGAAAGTGTCAGGTTCGGAGTGCATGTGTTCTGAAGAAATGAGTTCACTCCTGGAGGATTAAACAGTGTTTTTTGCATATAGTACCTGTTAGCCAGAACAAAATCGGCTAGAGCGGAGATGTCGTCGGCCGACACGTTCACCGACGTGCTCTGCACGGGGATCATGTTATCCATCACACTTGAAACGAAGGACTGGTTGGTGCCGGTGACGAGGAACCTCGCGGCGCAGGACCCAGGCGCCGCGGCGCCGGCGCTGCACGACGCGCCGCCGTCGTAGAAGAACGGGTTACTCGACGATGGCGACGGGATCTGCAGCACGGGTGGCCATTTGGGGGCGCGAGGCAGAGGGCACGAGTCCGAGCACACCGCGCCCCCCGTGCTGTTGCCCGACACGACGGCATTGTTGCAGGAGCAGTCCACGAGGATTGGATGTTCTCGCTCCTCCTTGGCTATCTGTCGGTCGATGGTGATCTGCAGGCCGCCGATGGCCCCGCAGATGATCAGCGGGAAAAAGATGAGGCAGCAGTTGGTCTTGCGGGCGCGCCTCTGGATCACTATATTCTTCATGAAGAGCGCGTTGGTCTGCTCGAGATTGCTCGAGCCCATGGCGCCCCTTCTCTCCGTCGGAGAACTCGAAG

1. Gene ID: DN93260_c0_g1

TAGAGGTACTCACATTCGCGGCCGACCTCAAACACAGAAGTAGCCATGGTGGACAGCGTGGTGAGCTCGGCGATGCAGAAGCTGGAGGGGATGGCGTTCGCCACGGTAAAGCAGCAGACGGAGGTGGGGAAGAGGGCGCAAGGGCTCAAGGATGACATGGCATGGCTGCAACTGGTGCTGCGGGGCGCCGACCAGCGCCGGCGGCGGGAGATCAACGATTACATCGAGCTGTGGGTGCGGCAGACGCGCGAGGTGGCCTTCGACGCCGAGGACCTCCTCGATGAGTACTACCACGAAGGGCGGCTGCACTGCCGCGGCGTCCTCGACCTGCCGTCCTTCCTCCGGTGGCTCCGCCACTCTGCCACCGGCCTCTTCGTCCGCCAGTCCATCTGCAGCGACATCGACGACATCAAGGCCAGGCTTGAGCAGATCAGGAAGAAGACGGAGGACAACAGCGTCCAGCTGAAGATGAGCCTCCCCGCCACCGCCAGCAGCGTTAAGCCCCGGAAGCGCTACGTGGACTGGGATGCGCCAAGTGGATGCAACATAGACAACCTGTTGGTAAATAATGAAAAATTGAAGAAAATCAAAGGCTACCTGAGCAGTCAAGGAGAAACAATGCCAAGAATTATAGTCGCCATCATGGGGAAGAGTGGTGCCGGGAAGACAACATTGGCTAGGTGTGTCTATGAGAGCAGCGAGGTCAGGGCCATGTTTCACCACATCATCTGGGTTCATTTACCCCAGAAATTCAGGCTGGTGGATGTCATTGCCGACATGGTCAGGCAAACCACATTCCCAGGAATGGACTTGGATCCGGAGAAGGATGTACATGAGAAGAATGTACATAAGAAGGATGTATATGACATTCGTCGGCTCAGAAATAGCCTCACTGGAAGACTCAATATGAAGAAGTACCTGATCGTGCTGGACAATGTGCGCAGCCCGGATGAATTGAACTTGTTCTTGTCCGTGCTACCTGAATGCAAAGGTAGCGCATTGTTGATCACGACAGAGATTAAACCTGACCATACGGCTTGTGGTTCCAGTTCAACTATGAATGACTCAGATACCCGTTGGAAGTTCAAGAAGCTATGTGATCATCAGGTGGTGCTACAAAAACTGAAGAAGGAAGAGGCTAGAAAGATGTTCCTGATGAGGCTATTTGGAAAATCCAGTTCTGACAAGAATGTCAAGAATGCTACACATGCCAAGGTGATCGAGAACCTCCTCGACAAGAGCCTTCCATTGGCTGGCACTCTGTTAGCCGGGCTCTTAAGGACCAAAAAGGAGGAAGTGTGGACTGATGTAATAAATCAACTCATGCAACAAAGGCACGAATTAGAGCAGCAGAAACAGAACCATGAAAAAGAGCAGGAACAACAACAGCAACAGTTAGCAGAGGGTCTAGAAGAAATGAAGAGGATGGACCAGCAACGTTCATGGCGACAAGAAGAAAAACAGCAGATTATAGACGAAAAGCAAGAAGTTAAGCTGCCAGCAGGGCAGCAAGAAGGACAACAAGAAGAAATAGAGCATGCACAGCTAGAAGGGGAAAAAGAACCTGAGCAACAGCCGGAGAAGCATGTATACCGGCAGATGTCTCTGCTGGAGCAAATACTCATGTTGAGCTTCGACGACCTCCATCCTCAACTCAAACAATGCTTCCTCTACTTTTCTGCATTCAAGGCAGAGGAGCCTATCAACGCGGACAAGCTCATCCGGTTATGGGTAGCGGAGGGGCTTGTGCGACCCACTGACGGACGAGCAGCGGAGAAGCACGGCCGAGATCATCTCCGGACACTGATCTCTAGGTGCTTGGTCAATCTGGTCGAGAAGGACTACAGCAACAACATCATCAGCGTCAGCATGCACGAGCGTGTCATCGCCTTTGCAAGGTCAGAGGCGCGTGAGATCAACTTCCTCCAGGTCCATCACAGCACCTCTGACCTTCCGAGCACAGCCATTCGCCGCCTCTCTGTCCGCAATGCATTTGATCCACACACAAGGCTCGCCCTCGCAACCCCGAAACTTCGATCACTGTTGTGTGAGTGTCCAGAAGCACCCTATGCCGATGACGGCTCCACTAGCTTTACTTCACACATCCGCACAGTATGGGAGTACATTGGAGGCCGCGTGATAAGCTTGAACATCCACCAATGCAAGTTTCTGCGTGTGATTGACCTCCAAGGAATGGTGCACCGATCAACCTTGCCATACGAGATTGGCTGGCTGATTTATCTCCAGTACCTGGGGCTTGCGCGTACCGGGTTGAGGAAACTTCCAAGGTCTATCAAGAATCTTCACCGTCTGCAAACACTGGATATCAGCAGCACCGAAATCAAGCATGTTCCAAATGGTCTTTGGTGGATTAAATCACTCCGGCATGTGCTGGCAGAACAGCTCGATAATGGCCCAACCAACAACAATGCTTTGCACAACCTCCAGACCCTCCATACGGTGCAATGCAAAGGCTCTGCACTGAAGAAGCTGATCAACCTCCGGTCCCTACGACTATGGGGAATCAATCAGAAGCTAATGCTTGCGGATTGTCTCGAGAGAATGGAGTGCCTCAAATTCTTGGATTTAGCAGCAAATGATGGGGTTAAGCTCCCATTGGTCGACATGCTAACCATGTTTGGACTGCGCAGCCTTCAACACCTCAAGTTAGATGGCCGAGTCAGCGAGGAAGGCATCAGAAAAGTTCCCGCTTATTTGCTTCATAAACTCACCAAGCTCGAGCTCCAAAACTCAGAAATGGAGCAAGGACATATGGACCTCATCGCCAAGGTACCAAACCTTGCTGGTCTTATCCTTGGGAAACATTCATACACTAAACCAGAAATGAATATTCCGACGGATGGGTTCCCAGAGCTAAAAGACCTCCAGATCAACAACTTGAAAGAACTGGAGGACTGGACTTTTGCTCAAGATGCTGGATCTACACTCAAGCAGCTTCAGCGAGTATCCATTCTCAACTGCACTGCACTTAAAAAGATTCCCGATGAGCTGAGAACACTACAGCATCTGGTGTTGTTCGCTGTCCGCAACAGTCCCGTGAATTTCCCATCTGGCAAGTTCGAGTCAGCGGAAAAGCTGTCAATCATCAAAGAAGAAAGTGAGGATAAGGTGTACCCTTCCACGTGAAAGAAGCATATGATTAGAAAAGTTTGAGTTTAGGGAGTCTGATTTGAGTACGATGGGGTTTTTTGTCCCGCAGAAGGTTTTCCCTGTCGCGTTCAGTCCGAGTCTGTCTAAGAGTGCTGAGTGTTAAATATTGTGTTTCACCATTTGCATGTATATCTGTATATTTTTTCATTGTACAAGTGGTTTGTTGCATACTTGCCTGTT

1. Gene ID: DN57749_c0_g1

GCCTTCCGTTGTTCTGCACCCTAGGGTTCATGAAGTCTTCAGCTAGTAGGTCGACGAGAGCCATGTAGTTCAGAATTCCTGCAGCGGCAGCGGTAAGGAGCCCTTCCACGATTAGTGCACTAGGACTGTTCTCGTCGTAAGTAGAGGATATCACAATGCCAATCACGACACCAAGTGGCATGGTGAGTGAGAAGAAGAACGCCATCATCAACACAGACTTCAGGCGAAACTGAGCCTGAACAATGCATCCTCCGAGCCCTATCCCTTCGAAGAACTGATGAAAAGTTAGCGCAGCCACGAGTGGTTTGATCGTGGTAGCATCCTCAGATGCACCTAAAGACATGCCGATGATCACCGAGTGCACTACGATCCCCAGCTCCAGCACCTGCGAGATGACGCGGTGGCGGATGAGCTGCTCGCCGTCGTCGGATTCCCCCGCGAACGACGACGATGCGATGGCGGCCGACACGCCGTGGAAGTGACCGCCTTGCGTGTGATCGGCGCCTGACGCCTCCAGGTACCCGGCGGCCGCGGAGACCTTCTTGGCGTGCGCGCGCTGGAAGTACCCCGTGGCGATGGTGTCGACAACGAGGGTGGCGATGGCTGCTAGCATTGCGACGAATCCGGCGAATGGGAATTTCTGCCACGGCCCATCGACGAGGCACGGCGAGCCGAGCCTCTCGAACGCTTCCGGGAGGATGTGAACGAAGGACGTGGCGAGGATGACCCCCGCAGCGAAGGCCTTCACGGCGAAGAACAGGTCCGTGTCCGGGCTCAGCGCCGGGAACTTCCGTCCAAGCGAGGGGATGCCGCAGCCCGCAGCGCTGGCCACAAGGATGCAGAAGATGGCGATGATCTTGAGCTTCAGCGCACCCGCCTTGTCGTGCTCTTCCTCTCCGGCCTGCTCGCACTCGCACTCGGCGTGCACGATCAGAGGGAGGGAGGAGACGACGAGGAGAAGGAGGCACAACGCGGCGAGCTTGAGATTGGCAGCCATGATATTTAGTCGGAG

1. Gene ID: DN93774_c1_g1

GAGGGACATGTAGTGGAACCACAGCCAGTAGGCCGGAATCCTGTCCCGGTTGATGAAGAAGCCGCTGAAGAGCAGGAAGTAGGCCAGGATGGCCACCACCACCGTGTACCCGATCATGACGTGCGGGATCACGCCGGAGAGGAACGTCACGAAGCCGCTCCCGGCCCAGAACGAGGCCAGGATTGCCAGCGTGTAGAAGGCGAACCCGGACACGCCGCCGGCCAGGCCGACGGCGAAGAACGTGGTGAAGGCGAAGGCGAGCGAGAGGACGACGAGCGGCGGGAAGGAGACGATGGCGTTGGAGAGCACGTAGGAGACGTGGCGGTACGCGCCGTAGGCCGTCTCCCGGAGGAACACGTAGCGCTCCTGGAGGAACACCGGGAGCGCGTCGGCGCAGGTGTAGAACATGGTGGACATGGCGAAGGCGAAGAAGCCGAGCCGCTCCTGCGCGCCCTTGGGCGACTGGTCCAGGCGGAAGAAGACGGTGGCCAGGATCACGCCGGTCACCACCACGGCGCCCAGGCGGATGAGGAACAGCTCCGGCATGCGCCGCGTGTTGAGCGCCGACCGCCTCGTCAGCACCTTCATCTCCACCCAGAACGGGTTCGCGTAGGACGTGCGCATCTCCCCGGCGGCCACGTCCGCGCCGGACACCAGCTTCCCGCGCGAGACGCTCGCGCTGATGGCTTCCTTCAGCGACATCGTCGGCGCCCACGCAGCCGCGTCGGGGCTCGCCGCGTGCATGACCTTCCACGAGCGGTGGAAGTCGACGAGAGGCTTCGTGCCCGT

1. Gene ID: DN79723_c2_g3

TGCCAGCCAGCCGGAGTCAGATCGATAGTGATATGGAAGATCAATCTCCATTCTAATCAGTTGATCTCACCTCCTTCTCCTCCGTTAGCATAATACTACTAACTACTCCTTCCTTTTATGCACTCTCCTCTGAGCTAGCTATAACAAACTAGCTAGCAGTGAAAGCAAGCCATGGAGGGTCCGGGGCCGGGGCTAGGGGGCACGCTGTCGTCCAGCCGCCGCGGCAGCGGCAGGAGCTGGGGCAGCTCCATCTCGCACTCCTTCCGCCATCAGGGCCTCCAGGCCGACACCGACGACCCCTTCAGGCGAGGCTCCCGGCGGCACGACGACGATGACGACGAGGAGAATCTGCGGTGGGCGGCGCTGGAGAAGCTGCCCACGTACGACCGCATGCGCCGCGCCATCGTCCTCCACGAGTTCGCCGACGGGGGCCTGGTGGAGATCGAGCACCTGACCAGCGGGGAGGGCGGCCGCGCGCTGCTGGAGAGGGTGTTCCAGGACGACAGCGAGCGCTTCTTGACGAGCCTGAGGGACCGGGTGGACAGGGTCGGTATCGAACTCCCGGCCATTGAAGTACGCTACCAGGATCTATCCGTCCAGGTGGACGCCTTCGTCGGGAGCCGAGCCCTGCCCACGCTTTGGAACGTTACCACCAACTTCCTTCAGGTATCTCATTGTCACCATGCATATCATGCTCGGCTTCATCTTTATTTTACCGTCCCCTTGTTGTCAAGGCCATCTTCAGGTGCCAACCTATTTTGAATATCAAAGTGTCTGCATCGGTTTGTTTGGGCCGGGTCACGAGCCTAAAATCGGCTATGTCTTTTCTCTTGTTCGCTTGGGTCAGGTAGCCCAGCAGCTCTTGTGCATTTTGGTCCGCTCTCACATTTTAGTTTTTTTTTTTACTATTTCACCAAAATATGCCAAATATATGAAATGGAAAACACCACCAAAACTGAATATAGTGCTAATAAATCACCAATAATTGGGGAATCACATAGTTCATACATACTACAAAATCAAAGTGCAAAAGGCACAAGTTTTATGTGAAATAGACAAACTGTGATAGATATGTGTGATCGCGCATCGCGTTGGCTTCTTGACGAGCCTGATGGACCTGGTGGGAAGAGTCGGTATCAAACTCCCGGCCATTGAAGTACTCTACCAGGATCTAACCGTCCAGGTGGACGCGTTCGTCGGGAGCCGAGCCCTGCCCACGCTTTGGAACGTTACCACCAACTTCCTTCAGGGTCTTATTGGACGACTGGGATCCTCCAACAAGAAAACCATCAACCTACTTCGAAACGTCAATGGCATCCTCAAACCATCAAGAATGACTCTTCTTCTTGGACCTCCTTCTTCAGGAAAGAGCAC

1. Gene ID: DN89057_c1_g1

CTAGGGGGCAATGCATTGTTGCATACTGCACGTACAGGATACAGTGCGCGGTAACGCGCGCGGGCAGGAGATCCGTCGCACGGTCAAGCCCACTTGGCCATGACGGACATGCCGCCGGCGCCGAGGAGGACGGCGGTGAGGCAGATGAGCTGGAGCCTGACGCTTCCCTGCAGCTTGGGCCCCATGAAGTCGGCGGCGAGGAGCTCCACGAGCGCCATGTAGTGCAGCAGCCCAGCCGAGGCGGCGTTGAGCAGGCCGACGACGATGAGCGCGGTGGGGCTGTTGTCCTTGTAGACCTTGGTGAGCGCCAGGCCGAGCGCGATCCCGAAGGGCGTGGTGGTGGAGAAGAAGAAGACGAGGCCGGCCTTCATCTTCATGCCGTACTCCGCCTGGAGGATGCAGCCGCCGAGGCCCATGCCCTCGAACATCTGGTGGAAGCACATGGCCGCCACCAGCGGCCGGATGGTGCACACGCTCTGCGACGCCCCCATGCCCAGCCCGATCACCACCGAGTGCACCACGATGCCCATCTCGAGCACCTGAACGACGACGCGGTTTCGGCGGAGCTGCATCTTACCAGCCTCGGCGTCGTTTGCTACGGCCACGCCGTGGCCATGACCGTGGCCGTGACTGTGCCAGTGTCCTCCTTGCTCCGGGCTCTCGTGATCGGCGACCACAGCGCTGCTGGGGCCGCTAGCTCCGCCCTTCTTCCGGTTGTAGAAGGTGAGCATGAGCGAGTCGACCATGAGCGTGAAGATGGCGGCGAGCATGGCGACGAAGGCGGTGAAGGAGAAGTCACCCCACGGCTTCTTGGGGAGGCAGGGCGAGGTGAGGTTGTTGAAGGAGTCCGGCAGCACGTGCATGTATCCGGTGCCGAGGATGACCCCCGACGCGAAGGCCTTGACGACGGAGAAGAGGCTGCGGTCGGGCTGCAGCGCCGGCACGGACTTGGCGAGGAGCGGCAGGCACACGCCGACCACGCTCGAGAACAGGATGGTCGGGATGGCGATGAGCTTGAGACGCCGCGCCTTGGGCACGTTGTGGCATGCGTCGGGCGCCGCCGCCGGGTCGGCGCACAGGCCGGCCTCGGGCGCCTTCTGCGCGTGGGAGAGGAGGGGAGAGGAGGAGCAGACGATGACAATCAGGAGGGCCAGCGCACGGGCTTGGGACGGCGGCGCCATTGCTCGATCTTGGCTTGGCTGAGGTTGTTGTACGTAGCTAGCTGGGAGGGAGGGAGTGACTTAGCTTGCTTAGTGAGCAGCTGTCTCTCTCTTACTACTTCTGCAACTGCAAGGGTCGTTGTGTACTTGGGTACTTGTACGGTTG

1. Gene ID: DN99764_c1_g3

GGAAGTAGGCGAGCGTGGAGACCACGACGGGGAACCCCAGCTGCACGTTGGTGACCACGCCGGAGAGGAAGGTCGCGAAGCCGCTCCCGGCCCAGAAAGAGGCCAGCACGATGGCCACGAAGAAGAAGAAGCCCTGGGCGCCCCCCGCCAGCCCAACGGCGAAGAAGGTGGTCAGCGCGAAGGCGAAGGAGAGGACGATGAGCGAGGGGAAGCCGACGACGGTGTGGGAGAGCACGTAGGAGGAGCGTCGGTATGCGTTGTAGGCCGTCTCCCGGAGGAAGATGTAGCGCTCGTTGAGGAACACGGGCAGCGCGTCGGAGCAGGTGTAGAACATGGTGGACATGGCGATGGCGAAGAAGCCCAGGCGCTCCTCCACGCCCTTGGGCGAGTCGTCCAGGCGCCAGAAGATGGTCGCCAGGATGAACCCCGTGATCAGCACCGCGCCCAGGCGGATGACGAAGATCTCCGGGGTGCGCTTGGTGTTTATGAACGCGCGGCGGGTGAGAACCCCCATCTCGATCCAGAACGGGTTCGCGAACTTGGTCACCGCGGAATCCGTCGCCGACAACTCGGAGGGCACCGTCACATTGCCGTCGGTCGCACCGGACACGAGCTTCCCGCGAGAGATGCTGGCACTGATGGCCTCCTTCAGGGAGAGCGACGGCTTCCCGCCATCGTCTGCGTGTTTGATCTTCGGGCCCATGCGCTTCTGCCACGACTTGTTGTGCTCGACCAGGTCGCTGGCACCCTCCGGCATGGTCTCGAGCTCCCTGACGAGGTCGAGCGCGAACTCCGTTGGGTTCTCGTTGTCGTGGATGGGCTTGCCGAAATCGTGGAAGAAGGACGAGAGCGAGCCGGGCGAGCCGTAGTACACGGTTTGGCCGCGAGACAAGAACAGCAGGCGGTCGAGGAGGCCGAGGATGCGGTAGCTCGGCTGGTGGATGGACATGACCACGACGCTGCCGCTCTGGGCGATG

1. Gene ID: DN55310_c0_g1

CCGATGTAGATGAACACGTACTCCCTGGTCTTGCGCTCCATCGCGTTGGGGTCCCGGAGGTAGAACACCTCGATCATGTTGCTCATCACGATAGCAAACGTCGGGCCGATGAACCCAGACATGATGGACCCGATCGCGCCCAGCACGGTGTAGGGCCACTCCGGGGCATTCAGCTTGAGCAGCGTGAAGAAGTACCCCTTTGGCGCACGCCGGTCATTGTCGGCGTTCGAGACCATCTCGATGCGGCCATCGGCGCCGGTGCTGTATGAGTAGCTCAAGTTCCTCAGGCTTCCTGACCTGAGGCTCAATGACCGGGTGGACAGGGAGTTGCTCAGGCGGGATGAGCGGTTCTTGCGGGTCGATGACCCGCGGAAGTCACGGTTTCTGGCCATCTCCTGGAATCTGATCAGAGCTGCGTAAGCACCCGAGCTTCCTTTGGCGAGGAGCTCGTCATGAGTACCAGTCTCAACAACCTGGCCTTGCTGGATCACCGCGATCATGTCAACGCATCGTATGGTTGAGAGCCTGTGTGCAACCACCACAGTTGTCCTGCCAATCATTATGCGGTCAAGTGCTTCTTGAACAATACTCTCAGAACCTGCATCAAGTGCACTAGTTGCCTCATCAAGAAGAAGGATCTTCGGATTCTTCAGCATTGCACGGGCAATGGCAATTCGTTGCTTCTGACCACCAGAAAGCTGAAGTCCTCTTTCACCCACCTGAGTGTTATACCCATTAGGAAGAAGAGCAATGAAGCTATGGGCATTGGCAGCTGAAGCAGCAGCCTCAACCTCAGCCATTGTAGCATCTGGCTTGCCATAAAGAATATTCTCAATGATGGTGGTCGCAAAGAGGGCGGGTTCTTGATTCACCAAACCAATCTGATCTCTCAGCCATTTCAATTGCAGAGTCTTGATATCCGCATTATCCAGCAAAACTTGTCCCTGATTAGGATCATAGAACCGTTCTATCAGAGACACAACTGTGCTCTTTCCAGAACCACTGCCTCCAACTACGGCTGCCGTCTTCCCAGCAGGAAAGAAGAGCGAGAAATCACGGAACACCATGACATCCGGGCGGGATGGATAGCTGAAGGACACTTCCTTGAACTCAATGTTTCCGTGGACTTCATCCAGGCACCTTCCATCAGCCGAGTCCTGGACTATCGTCGGCCTCTGCCTTATCACCTCCAGTAGCTTGTAACCGGCAATCTTCCCTTTGCTGAAAGCTCCAAGGTTCGAGAATGACTGTCCCAGGCTCAGGCCTCCGACAATGGCGGAAAAAATCGCCGTGAATGCCTTTCCACCATCTGTCTGTCCACTCCGGATGAACACGCCGGCGTACCAGAACACCAGTGCCCATGACATGCAGGCAAT

1. Gene ID: DN102612_c6_g1

GCCCTGCTCGCTTGCAGTATAAGAGAACAGAACGAGGACCGCCGGGAGCAGGAGCAGGGGGAGGAGGCTCTGGCGGTACACATTTCGGTGGGTCTAGATCCAATGCCAAGGAGGTGAGCTGCTCTTGTGGCCGCCGAGCTCGCGAGGGAGAGGGCCAGAACAGTGATAAATGGCGGAGGAGACGGGGAAGGCGGAGGCTGCCGGGTCTGGGAGCTGCGGCGGCGGCGGGTGCGAGCCAGTGAAGAAGAGGGCGGAGCAGAGCGTGGCCTTCCATGAGCTGTTCAGCTTCGCGGACCCGCTTGACTGGCTGCTCATGGCGGCGGGGAGCGCCGGTGCCATAGTACATGGCGCCGCCATGCCGGTGTTCTTCCTCCTGTTCGGTGAGCTGGTCAACGGCTTCGGCAAGAACCAGCACCATCTCCGCCGCATGACCGACGAGGTGTCCAAGTACTCGCTCTACTTCGTCTACCTCGGCCTCGTCGTCTGCGCATCCTCATACCTGGAGATTGCGTGCTGGATGTACACCGGCGAGCGCCAGGTGGGCGCGCTCCGGCGGCGGTACCTGGAGGCCGTGCTGCGGCAGGACGTCGGCTTCTTTGACACCGACGCGCGCACCGGCGACGTCGTCTTCAGCGTCTCCACCGACACGCTCCTCGTCCAGGACGCCATCGGCGAGAAGGTCGGTAACTTCATCCACTACCTCGCGACGTTCCTGGCGGGGCTCGTCGTCGGATTCGTCTCCGCCTGGCGGCTGGCGCTGCTCAGCATCGCCGTCATCCCCGGCATCGCATTCGCCGGCGGGCTGTACGCGTACACGCTCACCGGGCTCACCTCCAAGAGCCGGGACTCGTACGCCAACGCCGGAATCATAGCCGAGCAGGCGATTGCCCAGGTGAGGACAGTGTACTCCTATGTGGGGGAGTCCAAGGCCCTGAATTCCTACTCGGAGGCTATTCAGAACACCCTGAAGCTAGGGTACAAGGCCGGGATGGCGAAGGGGCTTGGCATTGGGTGTACTTATGGGATTGCCTGCATGTCATGGGCACTGGTGTTCTGGTACGCCGGCGTGTTCATCCGGAGTGGCCAGACAGACGGTGGAAAGGCATTCACGGCAATTTTTTCCGCCATCGTCGGAGGCCTGAGCCTGGGACAATCATTTTCGAACCTCGGAGCATTCAGCAAAGGGAAGATTGCCGGATACAAGCTGTTGGAGGTGATAAGGCAGAGGCCCACAATAGTTCAGGACTCAGCTGATGGGAGGTGCCTGGATGAAGTCCATGGCAATATTGAGTTCAAGGAAGTGTCCTTCAGCTACCCATCTCGCCCGGACGTCATGGTGTTCCGTGATTTCTCGCTCTTCTTTCCTGCTGGGAAGACAGCAGCTGTGGTTGGAGGCAGTGGTTCTGGAAAGAGCACAGTTGTGTCTCTGATAGAACGGTTCTATGATCCTAATCAGGGGCAAGTTTTGCTGGATAATGCGGACATCAAGACACTGCAATTGAAATGGCTGAGAGATCAGATTGGGTTGGTGAATCAAGAACCCGCCCTCTTTGCGACCACCATCATTGAGAATATCCTTTATGGCAAGCCAGATGCCACAATGGCTGAGGTTGAGGCTGCTGCTTCAGCTGCCAATGCTCATAGCTTCATTGCTCTTCTTCCTAATGGGTATAACACTCAGGTGGGGGATAGAGGACTTCAGCTCTCTGGTGGCCAGAAGCAGCGAATTGCCATAGCCCGTGCAATGCTGAAGAATCCAAAGATCCTTCTCCTTGATGAGGCAACTAGTGCACTTGATGCAGGTTCAGAGAGTATTGTTCAAGAAGCACTTGACCGCATAATGATCGGCAGGACAACTGTTGTTGTTGCACACAGGCTTTCAACCATCCGATGCGTTGACATGATCGCGGTGATCCAGCAAGGCCAGGTTGTTGAGACCGGTACTCACGACGAGCTCCTCGCCAAAGGAAGCTCGGGCGCTTACGCCGCTCTGATCAGATTCCAGGAGATGGCCAGAAACCGTGACTTCCGCGGGTCATCGACCCGCAAGAACCGCTCATCCCGCCTGAGCAACTCCCTGTCCACCCGGTCATTGAGCCTCAGGTCAGGAAGCCTGAGGAACTTGAGCTACTCATACAGCACCGGCGCAGATGGCCGCATCGAGATGGTATCGAACGCCGACAACGACCGGCGTGCCCCAAAGGGATATTTCTTCACGCTCCTCAAGCTAAATGCGCCGGAGTGGCCCTATACCGTGCTGGGCGCGATCGGGTCCATCATGTCTGGGTTCATCGGCCCAACTTTTGCTATCGTGATGAGCAACATGATCGAGGTGTTCTACCTCCGGGACCCCAACGCGATGGAGCGCAAGACCAGGGAGTATGTGTTCATCTACATCGGGACCGGGTTCTACGCGGTGGTCGCGTACCTTATCCAGCATTACTTCTTCAGCATCATGGGCGAGAACCTGACCACCAGGGTGCGGAGGATGATGCTTGCAGTTATCTTGAGGAACGACGTGGGATGGTTTGACGAGGAGGAGAACAACTCGAACCTAGTGGCGGCTCGCCTCGCCACTGAGGCCACGGACGTGAAGTCGGCCATCGCAGAACGGATATCGGTGATCCTGCAGAACATGACCTCGCTCCTGGTGTCCTTCATCGTCGGCTTCATCATCGAGTGGCGGGTGGCGATCCTCATTCTCGTCACCTTCCCTCTCCTTGTGCTCGCCAACTTTGCTCAGCAACTGTCGATGAAGGGGTTCGCCGGCGACACGGCCAAGGCGCACGCCAAGACGAGCATGATCGCGGGGGAGGGCGTGAGCAACATCCGCACGGTGGCGGCGTTCAACGCGCAGGACAAGATCCTGTCCCTCTTCTGCGGCGAGCTGCGCGTCCCGCAGGCGCACAGCCTGCGCCGGAGCCAGATCTCCGGCGCGCTCTACGGCCTCTCCCAGCTCTCCCTCTACGCCTCCGAGGCGCTCATCCTCTGGTTCGGCGC

1. Gene ID: DN56950_c3_g2

GCCGGGTCCTTGAGCACGGCGCGGGCGATGGCGATGCGCTGCTTCTGGCCGCCGGAGAGCTGCACGCCGCGTTCGCCGACTGGAGTCCTGTAGCCGTCGGGGAGCGCGCTGACGAAGCCATGCACGTTGGCGACCTTGGCCGCCTCGATGACCTGCTCCTCCGTGACGCCGTCCTTGCCGTAGGCGATGTTCTCCAGGATGCTGGTAGCGAAGAGGACGGGCTCCTGCTGCACAAGGCCGATCTTGAGGCGCAGCGACTTGAGGTTGAAGCGGCGGATGTCCTTGCCGTCGATCAAGACCTTCCCGGCCATGGGGTCGTAGAAGCGCTCGATGAGCGCGATGACGGTGCTCTTCCCCGACCCGCTCGCTCCCACGAGCGCCTGGCTCTGGCCGGCCCGGATCCTGAGGCTGAAGTCCTTGAAGACCATCACGTCGGGGCGCGACGGGTACGCGAAGTCGACGTGGCGCAGCTCGATCTCGCCACGTACCGACTCCACCTGCTCGGCCTCCGGG

1. Gene ID: DN62657_c0_g1

CTACCGGTTCAGCTATTTTGAAGCCTTAATTTTCTGTCCAATCCAATCCACCCGTCTTCTTCAGCTCAGGCCACTCACGGATGATCCGCCGGTAGTTCGGCCGCGTCGACTACATAGCCGCGGAGCGACGGCGTCCGGACCATCGCGAGCTCCGCCGCTCTTCGCTTGTGCTGCACCCTCCTCCTAGCGATGTATCCGCGCACCCACGGCGTCACGTCCTCACTGACCTTGATCATGACGAAGAAGAGCAGGCGGTAGATGATGATCATGCTGAACAGCACGGAGAGGTCCAGCCACTTGGAGCGCCTCACGTCGATCTGGAACACGTTCTCCAGGATGTACTCCCCTGGGATCTTGGGCAGCTCGTCGTCCTGGTTGTCGAACACCAGCCCCACCAGGTCGTTTTGGTATTGCCCCTGAAGTGCCCAGTAGTGGAAGCTGATGTATGACATAGGGTACCTCCAGAATGGTTTTGGGATGTCATGCGGGAGCCTGAAGTACCCTGATACCAGCATGAAGATCCCCTGTATCCCTGCGCCGATGATGATGCCCATGAGGAAGTTGGGAATGATGCTGGCGATGGCCATCATCAGGCTCTCGACGACGGTGACGCTGGCGTAGAGGCACAGCACGAAGAAGATGTAGTGGGTGAAGCCCGGGTGGAGGTGCACCATGAAGTAGCACAGCGTCCCTGACAGGAACGTGATGAGGATCAGGAACGGCATCGCCGACAGCGTGTTGCTGATCACGAACGCCAGCACGCCGTAGTGCCCGTTCATCCTCTCCCTCTGGAACACCTTCATGTCTTCCACGAAAGACGGGAACCCTCCGATGGACATGAACGTGACGAAGCCAAAGATGAAAGATGCGCACGCGCCCCGTGCCAGGATGGAGCTGTATTTGGTGCCGACGTTGAGGTAGATACTCCCAATACAGACTGTGACAACAATGTAGATGATAAGCCTCAACCAGTAGTACCCAAAGTCCCTTGACATGTTGACGAACGATCGCTTCGTGAGCGTGAAGGCCTGCATCCCAAAGCTTGCTTGGCTCCCCCCTGCATCCAGGACCGTTCCTTTAACCCGTGCCATCTCATCAACTTTCTGCCGTGCCACGAAATAGTACTGCGAGCGCTGGTAGTAGCTGATTAGCCTTCTGATTGCCTCGGAAGTTGTGGTTCGCTCGAGGGGATCGTCAGGCTTCTCAAATCTCATCTTCATAGATCCTTTCAGAGTGGCCTTCACCTTGTCGAAGTCTGCGTTTATGCAGCGCAGGAAATGATCCGATGGATTGCGCAGCGGTGGGCATGGGAAGCCTGCTTGGGCAAAGAACTCACAAGCCTCAGAAGCCTGCCCAAAGTACACTGTTTTGCCCCCTGAGAGGAGAAACAGTCGATCAAAAAGCTCGAAGACCTCGCTGCTCGGCTGATGGATCGAAGCGATCACAGTTCGACCGTCCCTAGCCAGCCCACGCAGCGTCTGCGTAACGAAGAAAGCTGAAGCACTGTCAAGACCACTGGTGGGCTCATCCAGGAAGAGCAGCCTAGGCCTCATCAGTATCTCTAGGGCAATGCTGACCCTCCTCTTCTCACCACCACTGACCCCCCTCAGGTGCCAATTTCCGATGACCGTGTCGGCGCAGTCCTGGAGCCCCATCTCGACAATGGTGCCCTCGACCAGGGCACGCTTCTCTTCCATGGGCATGTTGTCAGGGAGGCGAAGTCGGGCCGAGTATGAGATAGTCTCCCTCACCGTCAGTGTGCCGATCAAGTTGTCATCTTGCGTCACATAGGCCGCGGCGCCGAAGGAGAGGTTGGCCTTGCGGCCGTTGAGGAGGACGGTGCCGGAGAGGAAGGCGTTGGCGGCGAGGCGCCCCGCGAGGGCGTCGAGCATCGTGGACTTGCCGGAGCCGGAGGGCCCCATGAGCGCGGTGATGGTGCCCGGCTCGGCGTACCCGGTGAGCCCCTCCAGCACGGCCTGCGTCTCCCCGCTGCCGAGCGCCACGGTGACCGTGAGGTCCCGCCACGCGAGCCGCGCCGACACGTCCCCGAGGAACTCCACCGCCGCCTTCTCCCGCCACAGCGTCTCGCTGAGCGGGCTCATCGCCGGCGCCGACGCCACCGCGACCGCCCGTCCCGCCGCCTTCCCCACGTCCTCCAGCTCCGC

1. Gene ID: DN87048_c1_g1

GGGTACTTTTCTGCTTCTCATTTCCCTTCTTGCCCCGAGAACCAGAACCGGGCGTGTACTCATCTTCTTCGTACTCCTCGTCGTCGTCATCGTCCTCCTCTTCATCTTCCTCCTCCTCGTCGTCGTCGTCGTCCACCCACTCCTTCTTCACACCCCCATTGCTCCCTTTCCTGGCCCGAGACTGTGCTAGCGGGACGTCATCCTCATCGTCCTCCTCTTCCTTCTTGACCTTGGGGGCACCCGCGCCGCTCGATTTCTTCCTGTCGCGGGAGAACGCCAGCGGCACGTTGTCGTCGTCGTCGGAGTCCTCCTCCTCGTCCTCCATGTAGGGCGGGTTCAGCCCGGCGGCGGCGGCCCGGGACATTGCGTCGGGCGGCGGCGGCGGCGCTGGGGGGATTGCGCCTGTCGGCCGATGGGGAGGGGACGAGGCAGAGGCAGAGGCGGCGCTCGCGGTGGTGCCTTTGCCTTGTGTGGTTC

1. Gene ID: DN53412_c0_g1

CATCTATCTATCTCCCTGCAGGCCACACCAGAATAGACAAGGGGACACGTCACATTCAGACATAATCAGCACCATGTTGGCTATCAAGCATACCTTGCAAGCGCTTCCATGGCTCCTCTTGTTTGTGCAGCAGGCTGCAGCCAGCGGCAGCTGCGACTGCACTACAGCCACGGACGGGGCGGACAAGCAGGGTGCGACGAAGCTGAAGCTGGTCGCCATCGCGTCCATCCTGACTGCGGGGGCGGCTGGCGTGCTGGTGCCTGTGCTTGGCCGCTCCATGGCCGCGCTGCGCCCTGACGGCGACATCTTCTTCGCTGTCAAGGCGTTTGCGGCTGGCGTCATCCTTGCAACCGGCATGGTGCATATTCTGCCAGCGGCGTTCGACAGCTTCACGTCCCCATGCCTCCACAAAGGTGGCGGCAGTAGGAACGGCTTCCCTTTCGCGGGTCTTGTGGCGATGACTGCAGCAATGGCCACAATGGTCATAGACTCGCTGGCTGCTGGGTACTACAGGCGCTCTCACGTCAGTAAGGCACAGCCTGTTGACAGCATCGACATACCTGACCATGCTGGAGACGAGGAAGGGAGGACCGATCATGTGCATACGCAGGGCCATTCACATGGTGAGGTGGTGATCATCAGCTCACCAGAGGAGGCTTCAATAGCTGACACAATCCGGCACAGGGTGGTGTCTCAGGTTCTAGAGCTGGGAATCTTGGTGCATTCGGTGATCATTGGGGTGTCCTTAGGAGCATCTGTGAGGCCATCCACCATCAGGCCTCTGGTCGGTGCCCTTAGCTTCCATCAGTTCTTTGAAGGCATAGGCTTGGGTGGTTGCATTGTACAGGCTAATTTCAAGGTAAGGGCAACCATCATCATGGCAACGTTTTTCTCCCTGACTGCACCTGTGGGCATTGTGCTAGGGATTGCAATTTCATCTAGCTATAATGTGCATAGCTCTACTGCCTTCATTATCGAGGGAGTCTTCAACTCAGCATCAGCAGGGATTTTGATCTACATGTCCCTAGTTGACCTTCTAGCAACAGATTTCAATAACCCAAAGCTACAGACAAACACAAAGCTTCAGCTGATG

1. Gene ID: DN74651_c1_g4

ACTCGATCGATCACCACCACCATCCACGGAAGCGCCAACCACCACCACCTCCACTCCATCGATCGATCGCCAACTATTCTCGCTGTTTGATTTCCTTCTTCATCGTCTCCGCTTGATCGATCCTGTAGCATATCTTTGAGAGGAAACTGAGCTCGAGGTGCTATCGAACACGATGCCTCCGAACGGGCAGGGCATGCATGGCGGGGCCGCCGGCGTAGTCGCCTTACCGCCGCAGGCGACGCCGGCGCCGGAGTCGTCCAAGATGGACTGCTTCTTGACGTCCGTCTGCACGCCGCTCAACCTCCAGGTAGCTAGTTAGCTCAACACACGCATCATGCATGGTCTATGTCGCGTGCACGTGTGTGCTAGCTTGCTATCTGCGCGCGGAATCGCGGTTTCAAATCATCATGGACTAGTTTTTGACGGCGCGTCTTCTTTTGATGCTGTATGATTGCAGTTCATCGATGTAGCCTACCGCGTCAAGGTGGAGCGCACGGCCTCGGCGGGGGCGGGGAAAGAGCCGCCGGGGAGGATATCGCACTCGGGCGGAGGAGGCGGAGTCATCAGCGGCGTCGGGGTCGTGGAGGAGCGGACGATCCTCAAGGGCATCACGGGCGAGGCGCGGCCCGGTGAGGTGCTGGCGGTGCTGGGCCCGTCGGGGAGCGGCAAGTCGACGCTGCTCTCAATCCTGGGCGGCCGCATCTCCGGCCGCCACACCGGCACCGTGCTGGCCGGCGGGCGCGCGCCGTGCCGCGCCGTGCAGCG

1. Gene ID: DN96918_c1_g1

CATGTGCGTCGGCGCCACGGCGGGGAGGGCGCTCAAGACCGTGCTGCAGGGGATCCTGCTCTCGTCAGAAGAGGAGAAGCTCAACTCCATGGACCTGCTCCGGTACATGGCGCCGGTGGCCGTCGTGCTGCTGGTGCCGGCGACGCTGATCATGGAGCCCGACGCGCTCAGCGCGGCTGCCGCGCTCGCCCGGGCGGATCCCAGCTTCGTGTGGATGCTGCTCCTCAACTCCTCGCTGGCCTATCTGGTGAACCTGACAAACTTCCTGGTCACCAAGCACACCAGCCCGCTCACGCTCCAGGTCCTTGGTAACGCGAAAGGCGCGGTGGCTGTCGTGGTTTCCATCCTCATATTCAGGAACCCGGTGACCGTCATGGGGATGCTGGGCTACGGGGTCACCATCCTCGGCGTGGTCCTGTACGGCGAGGCCAAGAAGAGGAGCAAGTGAGCTCGACCTGGGGTCTCTTGTTTTTGCAACTCCAGTGTTCCAGGAGACACTTGATCTTTGGCCGGATCCCTCCAAGTTCCGGCCTAGTCCTGAATCCTGATTGTGTCACGATGAAGACGGCCAACTCATGTACTGTATTTCATTCAACATATATTCATGCCCCAGGTTGCAACCTGGGGTCCTTGAAGTCTAGGACTTTGTGCGTAAGCCCATAGATAAGAATGTACTATAGAGCAAGTGCCTGATTGATATTCGTATTCAGAGAGATGTACAGATACCATGAGGAACATGATACGCCTATAAAACTATTCAAGATAACATCAGTTACTCTCGTCGTGTAGTACCTGAGTAAAAACACTACTATTACCATAGCAAGTGAGATGAACAAATGGTAATCATGTACGCTTTACTCGCAAATCTGCAGCTGACATACCAGGTCTAGTGTTTTGCAGAACAGAACATCACAGTTTTTAACAATGTCCTGGGCTTTAACACTCGTAGAGCACTCGTTGAACTTCTAAATAAAACAACAAAAGAAAATGCAGGACAGTTTTTTTGTACTAACTTCAGCTGAAGTTGCCTTCTGAGTAGCAAGGGTGTAATCAGGAACAACCAGCAGCATTTCTTGTGGAACTCGGAGCCTCTACCAGATCAAGACACCTAGTCGGTGGCATCCTCTAGAGAAATGGAGGAAAGCTTCTGCGCCAACAGAGTCAACGGAGTCGCATCTCCATTTGCGGCTCCAAGAGAAATCGACGACAGCTTCTGGGTCAAGAGAGTTACTGGAGTCATTTCCCCTATAGCCTGCACATTTGGATGCGAAGCCAAAATGGATCCCTTCACTGGAGTCGCATCCCCTTCAGCTTGGATATTGTCATGCGACGTCAAAATGTTTTTCTTTAGTGGACTCGGAGAACTACTAGGATGGATATCCTGATGCAGGGCAGGAATGTTTTCTTTTGCAGTATCCACGGCCGCAAACACTGGCCAGGCGTGGCCCATGCAAGCTTCACGGTCTACAACTTCCGATGGTTCTACTTCCATTTCTGCAATTCCAAGAGGTATTGAAGGTGGGCAGGCCTGGGGCATGCAAGCTTCATTATCAATCACCTCTGATGGTTCTACTTCCATTGTTGCAACTCCAAGAGGCAGTGAAGGTGATGTCTTACTTTGAGAGGCTTCAGGCTCTATCAACTCTGATGGTTCTACTTCAGTTTGTGCAACATAAGTTCGGGATGGACTTTCCCCACCAGGTGCTGCTGTGGTGTCTACTTCCATTGTTGCAACTCCAAGAGGCAGTGACGCCTTACTTTGAGAGGCTTCAGGCTCTATCACCTCTGATGGTTCTACTTCAGTTTGTGCAACATTAGTTTGGGATGGACTGCTTTCCCCACCAGATGCTGCTGTAGTGTCTACTTCCTTTTTTGCAACTCCAAGAGGCAGTGAAGGTGATGCCTTACTTTGAGAGGCTTCATGCTCTATCACCTCTGATGGTTCTGCTTCAGTTTGTGCAACATTAGTTTGGAATGGACTGCTTTCCCCACCAGGTGCTGACGAAGTTTCTGATTCTTGTTGATCAATGGTACATGAGGACAGCTTAACTGTTGCAAGACTGCCATCACCATTGTCTTGGATAATCTGACGCAAAGCTGGAATGCTCTCCTCTGCAATGTCCACAGCGACCAATGGACCAGTCTCAAGCATGTGTGTTTCGTGCTGTGACACCTTCGATGAATCTACCTCTGTTTCTGCAACCCTGACAGCTTCATTATCAATCACCTTTGATGGTTCTACTGTAGTTTCTGCAACTCTGAGAGTCAGTGAAGGTGATGCCTCAGTTTGAGAAGCTTCTTGCTCCATCACCCTCGATGGTTCTACTTCAGTTTGAGAAGCTTCATGCGCTGTCACCATTGATGGTTCTACTTCAGTTTGCCCAACTTTGAGAGGCAGTGGAGGTGATGCCTTAGTTTCAGAAGCTTCATGCTCCATCACCTTTGATGATTCAACATCAGTTTGTGCAACTCCGAGAGGCAGTAAAGGCAATGCCT

1. Gene ID: DN81110_c0_g1

GCTAGGAATTCTCCACGGCAATCAGAACTCAGAAGGCAATTAATGGAGCTGATTCTGCACACCCATAAAATCAAGTTGCTACGTTACATGCATCTATTGTTTGAACAAGTCTTGTCTTGAAAAAAAATACTACTACATCATGATCTCCACAGAAGTAAGAAATTTTCTGTTAATTCTCGCTGCGTCACCTGCCCGATTCGTTGCGACGTCACTTGGCGGCGACTTTGGCTGCCGGCCTCTTCTTCCCGAGCTTGTGCCTGACCATCCTGACGATCCTCAAGAACCAGTAGGCACTCACCAGCAGCAAGCTCGTGGCCATCGTCTTGATGAGGATGGGGTTGTCGGCCGTCACGGTGCGGTAGGTGACGTACGTCCCACAGACCATTCGTGCCACGGAGAAGGTCGCCGCGAACAGAATATCGACGAGGAGGTTGAGGTCGGTGTCCTTGATGCCGAGCTCCTTGAGCATCTCCCTGAGGTGGAGCAGCGGCGCGGTGATCTCCGTGACGATCAGGCACGCCACCAGCTCCGTCCCACTCCTCTGGTAGGCGAGGCCGGCGCCGAGGCCGACGATGCTGATGAGGTGGTGCAGGGCGTTGTCCAGCCGCGCGTCGCCGCTCAGGTGGCAGCACGCCGCGTCGTAGATCATGTACGACAGCGTCACCGCCAGCGCCCTCATCTGGCTGGGCGAGGATGGGGATGCGACTGGGGAGACGGGCGACGCCCAGTCGTGCACGGAGAGGCAGCCGAGGCCCACGCCGGCGACGGCGTGCATTGTGGAGACGGCCCGGTTGCAGAAGTCGTAGGAGCGGCTGGGGAGCAGCGCCCGCAGCAGCAGGAAGGCCGTGGACCAGAAAGCCACCCCGGACGCCACCCAGCTCAACACCGGCACGCCGTGCTCCTCCGTCATTGTATGTCCCCGGCAGTCGAACGCGCGATCGACCACTCCAAGATCGGCAGGGAGATGTATGGTGGCGCACGCAGAAAGGGAGGGGAGGAAGAATCTATGAGGGAGATGTATGGTGGCTAGGTGCAGAGGCTGTGGGACGGTGGGAGGCAGGCAGCGGGGAAGAGAGCGGGCAGCAGGCAGCGTGGGTGCCCCATCAGGCCATCACCATGTGCAGCAGAGCAGTGCGCAGAATATTGGTGTGCCGCTTCATTGTAGGAGATTTGATGGG

1. Corresponding author. E-mail: [wubin824@sina.com](mailto:wubin824@sina.com) (B. Wu)

   Address: Chengdu University of Technology, 1# Dongsanlu, Erxianqiao, Chengdu 610059, Sichuan, PR China [↑](#footnote-ref-2)
